# Supplementary material for: Exploring the perceptions and experiences of community rehabilitation for Long COVID from the perspectives of Scottish general practitioners’ and people living with Long COVID: a qualitative study
Source: BMJ Open. 2024 May 15;14(5):e082830. doi: 10.1136/bmjopen-2023-082830 (PMC11097876; doi:10.1136/bmjopen-2023-082830)
Supplement: Author's manuscript [file bmjopen-2023-082830.draft_revisions.pdf]

BMJ Open is committed to open peer review. As part of this commitment we make the peer review history of every article we publish publicly available.

When an article is published we post the peer reviewers' comments and the authors' responses online. We also post the versions of the paper that were used during peer review. These are the versions that the peer review comments apply to.

The versions of the paper that follow are the versions that were submitted during the peer review process. They are not the versions of record or the final published versions. They should not be cited or distributed as the published version of this manuscript.

BMJ Open is an open access journal and the full, final, typeset and author-corrected version of record of the manuscript is available on our site with no access controls, subscription charges or pay-per-view fees (http://bmjopen.bmj.com).

If you have any questions on BMJ Open's open peer review process please email info.bmjopen@bmj.com

BMJ Open

Exploring the perceptions and experiences of community rehabilitation for Long COVID from the perspectives of Scottish General Practitioners and people living with Long COVID: a qualitative study

Journal:	*BMJ Open*
Manuscript ID	bmjopen-2023-082830
Article Type:	Original research
Date Submitted by the Author:	05-Dec-2023
Complete List of Authors:	Cooper, K; Robert Gordon University, School of Health Sciences<br>Duncan, Edward; University of Stirling, NMAHP-RU<br>Hart-Winks, Erin; Robert Gordon University, School of Health Sciences<br>Cowie, Julie; Glasgow Caledonian University, NMAHP Research Unit<br>Shim, Joanna; Robert Gordon University, School of Health Sciences<br>Stage, Emma; Robert Gordon University, School of Health Sciences<br>Tooman, Tricia; University of Stirling<br>Alexander, L; Robert Gordon University, School of Health Sciences<br>Love, Alison; Long COVID Scotland<br>Morris, Jacqui H.; University of Dundee, SNHS<br>Ormerod, Jane; Long COVID Scotland<br>Preston, Jenny; NHS Ayrshire and Arran, Occupational Therapy; University of Stirling Department of Nursing and Midwifery, NMAHP Research Unit<br>Swinton, Paul; Robert Gordon University,
Keywords:	Post-Acute COVID-19 Syndrome, REHABILITATION MEDICINE, Primary Health Care, Patient-Centered Care, QUALITATIVE RESEARCH

SCHOLARONE™
Manuscripts

I, the Submitting Author has the right to grant and does grant on behalf of all authors of the Work (as defined in the below author licence), an exclusive licence and/or a non-exclusive licence for contributions from authors who are: i) UK Crown employees; ii) where BMJ has agreed a CC-BY licence shall apply, and/or iii) in accordance with the terms applicable for US Federal Government officers or employees acting as part of their official duties; on a worldwide, perpetual, irrevocable, royalty-free basis to BMJ Publishing Group Ltd ("BMJ") its licensees and where the relevant Journal is co-owned by BMJ to the co-owners of the Journal, to publish the Work in this journal and any other BMJ products and to exploit all rights, as set out in our licence.

The Submitting Author accepts and understands that any supply made under these terms is made by BMJ to the Submitting Author unless you are acting as an employee on behalf of your employer or a postgraduate student of an affiliated institution which is paying any applicable article publishing charge ("APC") for Open Access articles. Where the Submitting Author wishes to make the Work available on an Open Access basis (and intends to pay the relevant APC), the terms of reuse of such Open Access shall be governed by a Creative Commons licence – details of these licences and which Creative Commons licence will apply to this Work are set out in our licence referred to above.

Other than as permitted in any relevant BMJ Author's Self Archiving Policies, I confirm this Work has not been accepted for publication elsewhere, is not being considered for publication elsewhere and does not duplicate material already published. I confirm all authors consent to publication of this Work and authorise the granting of this licence.

For peer review only

TITLE PAGE

TITLE: Exploring the perceptions and experiences of community rehabilitation for Long COVID from the perspectives of Scottish General Practitioners' and people living with Long COVID: a qualitative study.

AUTHORS

Kay Cooper*A, School of Health Sciences, Robert Gordon University, k.cooper@rgu.ac.uk

Edward Duncan*, University of Stirling, edward.duncan@stir.ac.uk

Erin Hart-Winks, School of Health Sciences, Robert Gordon University, e.hart-winks@rgu.ac.uk

Julie Cowie, Glasgow Caledonian University, Julie.Cowie@gcu.ac.uk

Joanna Shim, Robert Gordon University, j.shim1@rgu.ac.uk

Emma Stage, Robert Gordon University, e.stage@rgu.ac.uk

Tricia Tooman, University of Stirling, t.r.tooman@stir.ac.uk

Lyndsay Alexander, Robert Gordon University, l.a.alexander@rgu.ac.uk

Alison Love, Long COVID Scotland, alison.despard@ymail.com

Jacqui Morris, University of Dundee J.Y.Morris@dundee.ac.uk

Jane Ormerod, Long COVID Scotland, longcovidscot@gmail.com

Jenny Preston, Douglas Grant Rehabilitation Centre, Ayrshire Central Hospital, Jenny.Preston@aapct.scot.nhs.uk

Paul Alan Swinton, Robert Gordon University, p.swinton@rgu.ac.uk

*Joint lead authors

ACorresponding author

ABSTRACT

Objectives: To explore the experience of accessing Long COVID community rehabilitation from the perspectives of people with Long COVID and General Practitioners (GPs).

Design: Qualitative descriptive study employing one-to-one semi-structured virtual interviews analysed using the framework method.

Setting: Four NHS Scotland territorial health boards.

Participants: Eleven people with Long COVID (1 male, 10 female; aged 40-65 [mean 53], and 13 GPs (5 male, 8 female).

Results: Four key themes were identified: i) The lived experience of Long COVID; ii) The challenges of an emergent and complex chronic condition; iii) Systemic challenges for Long COVID service delivery, and iv) Perceptions and experiences of Long COVID and its management, including rehabilitation.

Conclusions: There are several patient, GP, and service-level barriers to accessing community rehabilitation for Long COVID. There is a need for greater understanding by the public, GPs, and other potential referrers of the role of community rehabilitation professionals in the management of Long COVID. There is also a need for community rehabilitation services to be well promoted and accessible to the people with Long COVID for whom they may be appropriate. Service providers need to consider availability and accessibility of Long COVID rehabilitation and ensure adequate interprofessional communication and collaboration to enhance the experience for people with Long COVID.

Strengths and limitations of this study:

- This is the first study to explore the issue of accessing Long COVID community rehabilitation from the perspectives of potential service users and referrers in the Scottish context.
- One researcher conducted all interviews, ensuring consistency in their conduct

For peer review only

- Data were analysed and interpreted by multiple researchers, including people with Long COVID
- The small sample size, largely drawn from health boards with a similar approach to Long COVID rehabilitation, limits generalisability

Keywords

Post-Acute COVID-19 Syndrome, Rehabilitation medicine, Primary Health Care, Patient-Centered Care, Qualitative Research

Word count: 7,007

Funding statement

This work was supported by the Chief Scientist Office Scotland, grant number COV/LTE/20/29.

For peer review only

MANUSCRIPT

INTRODUCTION

Long COVID in adults is a multisystemic condition described as signs and symptoms that develop during or after an infection consistent with COVID-19, and continue for more than 12-weeks, which cannot be explained by an alternative diagnosis [1]. While global prevalence remains unclear, the World Health Organisation estimated that as many as 34 million people may have experienced Long COVID by 2022. [2] In the UK, the Office for National Statistics estimated that 1.9 million people (2.9% of the UK population) had self-reported Long COVID as of March 2023 [3]. The effect of Long COVID on individuals varies. More than 200 symptoms have been identified affecting multiple organ systems including thrombotic and cerebrovascular disease, chronic fatigue, and dysautonomia, resulting in significant reduction in quality of life [4].

Healthcare professionals, and general practitioners (GPs) in particular, are faced with complex challenges associated with the diagnosis and management of people with Long COVID (PwLC) as they attempt to disentangle nonspecific symptomology associated with the virus from those related to other conditions [5]. The National Institute for Health and Care Excellence (NICE) rapid guideline on Long-term effects of COVID-19 recommends that individuals have access to rehabilitation that is personalised and multi-disciplinary in nature [1]. Subsequent studies have emphasised the importance of this approach [6-8]. However, uncertainty remains around the effectiveness of rehabilitation, with research ongoing [9 10]. In England, service provision for Long COVID has centred around the development of specialised clinics, usually comprising multidisciplinary teams that include medical, allied health and psychology professionals[11]. In Scotland, Long COVID rehabilitation is delivered in community (non-hospital) settings by generalist allied health professionals, with local variation in models of service delivery [12].

PwLC have previously reported barriers to accessing healthcare and difficulties in navigating interactions with disjointed healthcare services [12-15]. Kingstone et al. [16]

For peer review only

highlighted the importance of "finding the right GP" for PwLC accessing primary care. GPs are the first point of contact for PwLC and have an important role in ensuring that people receive appropriate treatment and are referred to specialist services, including community rehabilitation, when needed. GPs, however, continue to face uncertaintly around providing a Long COVID diagnosis and ongoing management and, furthermore, experience challenges referring PwLC onto appropriate diagnostic and interventional services [15 17].

There is a growing body of research on the lived experience of PwLC [18 19] including their self-reported barriers to accessing healthcare [13 14]. Until recently, however [15], none have focussed on community rehabilitation. There is also a growing body of research on the role of the GP in providing care to PwLC, including the challenges of diagnosis and medical management [17 20]. Brennan et al's scoping review [17] included 19 studies on the management of Long COVID by GPs. They reported five articles that described difficulties with accessing services, particularly those in the communtiy such as follow-up services and Long COVID clinics [17]. Bachmeier et al's qualitative study found that German GPs rarely referred their Long COVID patients to specialist care or rehabilitation [20]. This was partly due to referral restrictions in the German healthcare system and lengthy waiting times for rehabilitation services or post-COVID clinics.

The current study explored PwLC and GPs' perspectives of accessing Long COVID community rehabilitation in the context of four Scottish regional health boards. Rehabilitation in this study is defined as intervention/s aimed at optimising function and reducing disability [21], delivered by any appropriate healthcare professional in a community setting. Community rehabilitation typically takes place in clinics and people's homes. This work was embedded within a larger realist evaluation study called LOCO-RISE, investigating models of service delivery for Long COVID community rehabilitation in Scotland. The initial phase of the realist evaluation (November 2021 – April 2022) focussed on PwLC and service providers' perspectives, experiences, and outcomes of

For peer review only

Long COVID rehabilitation [15]. During this time period, we observed that in some areas, there were low numbers of PwLC receiving community rehabilitation services [15]. We sought approval for an amendment to the study (Ref: 21/WA/0118 A03) to understand potential reasons for these low numbers, in the context of the increasing number of PwLC, recommendations for providing community rehabilitation to PwLC [6 8 22] and knowledge that services existed across Scotland [12]. The study reported here aimed to address two questions: i) what are the perceptions and experiences of PwLC on accessing rehabilitation for Long COVID? and ii) what are GPs' perceptions and experiences of managing PwLC presenting with symptoms of Long COVID that may be suitable for rehabilitation? PwLC were defined as adults (aged 18+) with Long COVID (with or without a positive COVID test) living in the community (i.e., not hospitalised).

METHODS

Study Design

This was a qualitative descriptive study employing semi-structured virtual interviews with a convenience sample of PwLC and GPs in four Scottish health boards. The study followed an a priori protocol (Supplementary File 1) and is reported in keeping with the consolidated criteria for reporting qualitative research (COREQ) [Supplementary File 2] [23].

Participants

The four Scottish health boards were chosen for the larger realist evaluation study based on variation in population and accessibility (using the Scottish Government Urban Rural Classification 2020) [24], Long COVID prevalence, and Long COVID rehabilitation service delivery models. We recruited from these same health boards for this study. At the time of conducting this study, two health boards were offering an integrated Long COVID rehabilitation service (i.e., integrated within existing community rehabilitation pathways), one had recently launched a dedicated Long COVID community rehabilitation service, and one pre-existing dedicated Long COVID service was closed to new referrals

For peer review only

due to an increased referral rate combined with reduction in funding and therefore inability to staff the service.

A convenience sample of PwLC were recruited via social media accounts of the research team and their institutions and by Long COVID Scotland, a volunteer-led charity run by PwLC. Inclusion criteria for PwLC were the following: community-dwelling (i.e., not currently hospitalised); aged 18 or over; experiencing symptoms of Long COVID (with or without a positive COVID-19 test; and experience of accessing or attempting to access healthcare services for possible rehabilitation. Those interested in the study contacted the research team, were sent detailed study information, and provided informed consent (audio recorded) prior to taking part. A second convenience sample of GPs were recruited by email invitation circulated on behalf of the research team by the NHS Research Scotland Primary Care Network. The network coordinator provided eligible GP practices in the four health boards with an electronic letter of invitation and participant information sheet to be shared with GPs. Those interested in taking part contacted the research team and provided informed consent as described above. Inclusion criteria for GPs were the following: GP in a practice within one of the four health boards taking part in the study; and experience of patients with probable Long COVID who may be suitable for rehabilitation. Several recruitment reminders were issued, and we aimed to recruit 12-20 PwLC and 8-20 GPs in total.

Data collection

Interview topic guides [Supplementary File 3] were developed. While not formally pilot tested, the topic guides were refined by the research team, in consultation with people with lived experience of Long COVID and informed by initial findings from our realist evaluation study [15] and the wider literature in the field. During the period June 2022 to January 2023 semi-structured online interviews (Microsoft Teams) and one telephone interview were conducted by one research assistant [EHW], who received training and supervision from KC and ED. No repeat interviews were undertaken. GP participants were either in their workplace or homes when interviewed, while PwLC participants were

For peer review only

all at home while interviewed. The research assistant, a female, was a qualified nurse and had worked in critical care from the start of the coronavirus pandemic (March 2020) until December 2021. She had no prior relationship with the GP practices or participants who all understood that EHW was employed as a study research assistant. The interviews lasted 17 to 47 minutes (mean 23 ±7.8 minutes) and were audio recorded. No other individuals were present during the interviews. No field notes for analysis were taken. Interviews with PwLC were transcribed by an external transcription service. Due to the homogeneity of the GP interviews, these were not transcribed but instead were listened to on multiple occasions by two researchers [25 26]. Neither transcripts nor audio files were returned to participants for comment or correction.

Data analysis

Data were analysed using the framework method (Gale et al. 2013) [27], which proposes a matrix-based format to facilitate the sharing and management of data as a team. This approach is widely used in applied health research and recognised as appropriate for multidisciplinary research teams as it provides a structure to consider data within and across interviews. Familiarisation with the data began by reading and re-reading the transcripts (PwLC) and listening/re-listening to the recordings (GPs), making analytical notes that informed the 'working analytical framework' for each participant group [27]. Although line-by-line coding is common in qualitative research, it is also possible to develop a framework without engaging in explicit coding [28]; due to the small scale and nature of the data we adopted the latter approach. The framework method was used to construct matrices in Microsoft Excel, enabling the data to initially be summarised into broad categories. This was led by EHW, in close consultation with two experienced qualitative researchers (KC, ED). The charted data were then analysed by interpreting within and between participants to identify concepts, which were subsequently grouped into themes, and finally overarching themes consisting of data from both participant groups. This was an iterative process involving multiple researchers (KC, ED, JC, TT, JS, ES), the study team, and subsequent refinement until

For peer review only

there was consensus that the data had been comprehensively analysed. As with our previous study we did not seek participant feedback directly on the findings. We did, however, present the findings in a webinar attended by health professionals and PwLC. Webinar attendees endorsed this study's findings and reflected that the analysis reflected their personal experiences.

Patient and public involvement

Two members of the public with lived experience of Long COVID were core members of the study team (AL, JO). Both contributed throughout the study and were integral to its success, helping with design and identification of important issues to explore. They co-developed study materials and interview topic guides and contributed to analysis and interpretation of study findings.

RESULTS

Participant characteristics

Twenty-five PwLC expressed an interest in taking part in the study. Of these 25, one did not meet the inclusion criteria for this study, but they were invited to take part in the larger realist evaluation. Thirteen PwLC did not pursue interest in participating in the study. Sixteen GPs expressed an interest, of whom three did not proceed to interview due to logistical challenges. Therefore, eleven PwLC (1 male, 10 female; aged 40-65 (mean 53)) and 13 GPs (5 male, 8 female) provided informed consent and took part in an interview. Figure 1 displays the numbers of participants recruited from each of the four health boards and Table 1 describes participants' demographics. No participants who consented withdrew from the study.

Findings

Framework analysis resulted in four overarching themes: i) The lived experience of Long COVID; ii) The challenges of an emergent and complex chronic condition; iii) Systemic

For peer review only

challenges for Long COVID service delivery, and iv) Perceptions and experiences of Long COVID and its management, including rehabilitation.

Table 1 Participant demographics

**GPs**	**n (%)**	**People with Long COVID**	**n (%)**
**Sex**		**Sex**	
**Male**	5 (38)	**Male**	1 (9)
**Female**	8 (62)	**Female**	10 (91)
**Years experience primary care**		**Employment status**	
		**Employed**	4 (36)
**1-10**	5 (38)	**Long-term sick leave**	4 (36)
**11-20**	4 (31)	**Left employment due to Long COVID**	3 (27)
**>20**	2 (23)		
**NR**	1 (8)		
		**Co-morbidities**	
		**Yes***	5 (45)
		**No**	6 (55)

Key: GP=General Practitioner; NR=Not reported;*co-morbidities included respiratory and thyroid conditions, and ME (Myalgic Encephalomyelitis)

Table 2 identifies the data and participant groups that contributed to each of these themes. Throughout the manuscript participants are referred to by a unique, anonymous identifier (health board A-D, followed by PwLC or GP, followed by participant number).

I: The lived experience of Long COVID

PwLC spoke of how their symptoms negatively influenced their quality of life. These included physical symptoms such as breathlessness and mobility issues, as well as cognitive difficulties such as 'brain fog' and problems concentrating. Fatigue was most frequently reported, with PwLC describing themselves as feeling physically and emotionally exhausted, resulting in a reduced capacity to participate in activities. Some PwLC were unable to engage in relationships or felt the need to reduce their level of engagement to preserve energy.

> *I was desperate, I just had no quality of life at all. I couldn't speak to my friends for coughing, couldn't look after my family because I had no energy, couldn't get out the house, I was housebound for months. It was just rubbish.* [APwLC05]

For peer review only

Living with Long COVID had a profound impact on participants, with many describing the experience as significantly life changing. PwLC reported the deterioration in their physical health as leading to a sense of frailty.

This often caused frustration around their inability to participate in activities that were perceived as important, and a need to adapt and find new ways of managing daily life.

> *It is causing me to think now every time we decide to do something, 'Am I going to be able? What are we going to have to do in order to facilitate that?' And I'm not eighty-five. I wasn't ready to have to start thinking about this right now.* [DPwLC05]

The psychological impact was characterised by PwLC as a struggle to come to terms with a new way of being, and a recognition that the internal expectations that one holds for oneself were being challenged.

For peer review only

Table 2: Overview of thematic analysis

Higher order themes	Themes	Subthemes
**I: The lived experience of Long COVID**	**PwLC**<br>Impact of Long COVID on daily life	**PwLC**<br>Psychological impact on Long COVID<br>Physical symptoms of Long COVID<br>Impact of Long COVID on quality of life<br>Impact of Long COVID on ability to work
**II: The challenges of an emergent and complex chronic condition**	**GP**<br>Long COVID as a chronic condition	**GP**<br>Challenges and uncertainty associated with diagnosis<br>Complexities of Long COVID and similarities with chronic conditions (ME/FM/CFS/post-viral)<br>System challenges associated with chronic conditions
	**PwLC**<br>Long COVID as a new and unknown condition	**PwLC**<br>Stigma associated with Long COVID<br>Invisibility of Long COVID<br>Lack of validation<br>Complex symptoms and lack of diagnostic/confirmatory tests<br>Lack of ownership by any given discipline & related difficulty getting referred to secondary care<br>Need for self-advocacy among people with Long COVID
**III: Systemic challenges for Long COVID service delivery**	**GP**<br>Long COVID management in primary care	**GP**<br>Current pathways/lack of pathways<br>Resource issues<br>Training needs<br>Safety netting: need for tests and investigations
	**PwLC**<br>Barriers & facilitators to accessing healthcare support for Long COVID	**PwLC**<br>Lack of referral pathways and access to GP and/or secondary care<br>Lack of available resources with long waiting lists & limited support<br>Need for joined up and person-centred care, especially follow-up on results of tests & investigations<br>Apparent inequities across health boards
**IV: Perceptions and experiences of Long COVID and its management, including rehabilitation**	**GP**<br>Perceptions of Long COVID	**GP**<br>Knowledge & beliefs about Long COVID prevalence<br>Perceptions of symptom presentation<br>Knowledge & beliefs about risk factors

For peer review only

		Knowledge & beliefs about subgroups affected by Long COVID<br>Work-related issues and need for vocational rehabilitation<br>Psychological impact and need for peer support<br>Beliefs and values relating to Long COVID
	**PwLC**<br>Healthcare professionals' knowledge & attitudes	**PwLC**<br>Experiences of support available<br>Importance of validation from primary care<br>Perceptions of HCPs lack of knowledge and understanding of Long COVID<br>Role of self-management in Long COVID<br>Role of pharmacology in Long COVID
	**GP**<br>Long COVID Rehabilitation	**GP**<br>Role of medical staff in Long COVID Rehabilitation<br>Role of allied health professionals in Long COVID Rehabilitation<br>Rehabilitation needs
	**PwLC**<br>Experience of Long COVID services	**PwLC**<br>Experience of support from allied health professionals : education & symptom management<br>Perceptions of usefulness of online resources (mixed views of social media)<br>Views on private healthcare<br>Perceptions and experience of 3rd sector support<br>Perceptions of peer support<br>Perceptions and experience of occupational health support

Key: PwLC=People with Long COVID; GP=General Practitioners; ME= Myalgic Encephalomyelitis/Encephalopathy; FM=Fibromyalgia; CFS=chronic fatigue syndrome

For peer review only

This was described as a sense of losing or reshaping of one’s identity, with many acknowledging that they no longer felt like the person they used to be.

> *I can't remember the last time when I had a day where I was thinking I feel like my old self…I think that last bit is the longest lasting effect of it and kind of the most debilitating, just feeling like a different and frankly lesser person.* [DPwLC06]

PwLC associated their physical health deterioration with a decline in mental health, with some PwLC experiencing negative feelings such as anxiety and depression. Participants also expressed a need to seek pharmacological and psychological support for their emotional wellbeing.

> *I also had - although it is something I suffer from, but I take Citalopram for anxiety- a very bad anxiety period as a result of COVID because to be honest I thought I was going to die, so that triggered a lot of other problems* [DPwLC05]

Living with Long COVID impacted some participants’ ability to engage in employment. Many attempted to return to work following the acute infection, with some returning to employment on a phased return. There was a perceived lack of support for return to work however, with some expressing a need for vocational rehabilitation to support return to work after a prolonged absence. Others spoke of the challenges associated with presenting at work whilst not able to fully function, which they attributed to a worsening of their physical symptoms.

> *Fortunately, I've had it, been off, but then went back to work. I've not stayed off. Maybe that’s a problem as well, I've not stayed off long term and listened to my body. I've went back and really threw myself back into my workplace and then suffered in my days off.* [CPwLC01]

The inability to work due to Long COVID created feelings of anxiety associated with the financial implications of losing income, and participants reported a lack of support and practical resources that could address their financial anxiety.

For peer review only

It is a worry for the future. I'm [age] *and I've got a bit to go until retirement. I feel like I'm in limbo, it's like Groundhog Day, and it's kind of scary what's going to happen with my work. I'm going to have to address that at some point because I can't see there there's an end in sight with everything. Yeah, it's another hurdle I'm going to have to address next year at some point.* [BPwLC07]

II: The challenges of an emergent and complex chronic condition

Due to the symptoms experienced by PwLC in this study, many had presented to primary care seeking confirmatory tests and onward referral and had felt frustrated when they encountered GPs who were reluctant to provide a diagnosis. GPs however spoke of the challenges associated with providing a diagnosis in the absence of a specific diagnostic test, and the limited treatment options that were available.

I don't know if we will move away from handing out the diagnosis of Long COVID. Because to be honest, I don't often suggest it to the patient as a diagnosis because our options for management are so minimal. So, I tend to, if the patient thinks they've got it, work through that with them. [AGP01]

GPs also drew similarities between Long COVID and existing chronic conditions, such as chronic fatigue syndrome/ME and Fibromyalgia, and a sense of familiarity around the uncertainties associated with diagnosis and management of these conditions, conveying a need for a service for long term conditions inclusive of Long COVID.

I think we are talking about Long COVID now because everyone is looking at COVID, which is great. But I think it's not the only kind of post-[illness] *treatment type problem that we have nowhere to send people. And I think it's a problem with the health system.* [BGP01]

People with Long COVID recognised the uncertainties associated with Long COVID. Many felt they were not heard or believed by healthcare professionals and the wider public however and expressed a desire for their symptoms to be validated, listened to, and supported. There was a sense of social stigma associated with living with an invisible

For peer review only

illness, which was often attributed to a lack of understanding about how Long COVID affects a person, and a feeling that they were being treated like a malingerer, and 'making up their suffering.'

> *There's definitely been a lot more media which is very helpful…there was a middle part where people were quite scathing because in the first part I remember friends saying 'I don't know anybody else that's got Long COVID', and that in itself felt a judgement, but then we hit a middle part where loads of folk were getting COVID but they'd been vaccinated or their bodies just dealt with it differently and they weren't ill, so then there was a huge period of judgement came out, 'well, so-and-so had it and they're fine'.* [BPwLC06]

Many PwLC felt dissatisfied that symptoms were not being taken seriously enough to gain access to secondary care and had an awareness of inconsistencies across health boards in relation to Long COVID services, suggesting a need for a single person to take ownership of Long COVID in their area.

> *"There needs to be at least one thing for each trust. Even if there's only one place in the whole of* [Health Board], *and obviously I know that I'm from* [place] *and that would mean it would be here, I just think there should be at least one that people aren't having to travel that long a distance to get to. Just somebody that has studied Long COVID, understands the complexities of it*...[BPwLC05]

The disconnection between patients' expectations and their experiences led some to the realisation that they needed to take control of their own health. One participant stated, *"You're almost left to fend for yourself"* [BPwLC07], whilst others highlighted the importance of advocating for themselves through researching symptoms, obtaining information from online resources, and using exercise to self-manage.

> *Oh, I've turned to Twitter…there is kind of a few people on Twitter that I follow that have been really good and kind of published research papers…I go back to the GP and ask about stuff, so I'm just having to kind of search for it myself…So*

For peer review only

you're not only having to deal with the illness you have to kind of then navigate kind of like, where am I going to get help from? [DPwLC02]

III: Systemic challenges for Long COVID service delivery

Healthcare system-related challenges included the lack of clear pathways to access, siloed specialised services, limited resources (long waiting times for appointments in primary and secondary care), and the lack of holistic care. PwLC described these challenges as contributing to communication breakdowns, a lack of accountability over chronic conditions, and the need for self-advocacy amongst people with Long COVID.

Every NHS trust should have at least one Long Covid clinic. It needs to have specialist people. All the therapists and all the scans and everything I've had have been done as standalone things, whereas at least if it was something centralised in a COVID clinic you would feel like you were having every part of your body tested and checked with Long COVID in mind……. It's the piecemeal part of it that's not looking at it in a holistic way. [BPwLC05]

GPs reported a lack of pathways and processes for managing patients with Long COVID, including identifying and tracking patients along the care trajectory and establishing consistent approaches to management. The nature of the emergent condition made it challenging to identify appropriate services to refer patients on to.

It's less easy to identify one individual you could refer them to so without an over-arching service it's hard to know where to direct the patients. [AGP02]

GPs reported that patients seldom met the criteria for existing rehabilitation programmes (e.g., cardiac/respiratory). They also reported that they often carried out tests and investigations to exclude other differential diagnoses as a means of 'safety-netting', but some were mindful that this could delay rehabilitation and recovery.

For peer review only

> *There is a risk that we hold up rehabilitative inputs until we fully investigated things and we are entirely assured that there's nothing going on. So, we should perhaps be blending things a little better.* [BGP02]

Both participant groups felt that limited resource was one of the main barriers to Long COVID management. People with Long COVID experienced difficulty accessing both primary and secondary care.

> *I didn't have the energy to argue with the receptionist at the GP surgery, and that's the honest truth, I just didn't have it in me to phone and try and explain it all again. So, I lost nineteen pounds in four weeks because I just couldn't eat because I felt sick. But even that wasn't worth the battle I would have to get past a GP receptionist.* [APwLC05]

GPs were aware of the significant pressure secondary care were facing which often resulted in long waiting times for specialties. As a result, patients often re-presented to primary care for ongoing issues leaving GPs with limited options for referrals and treatments.

> *We were getting a bit frustrated referring patients to secondary care for help. There wasn't much coming through. They were already dealing with backlog enough and they're getting piled up with these other things happening. I understand their limitations, entirely…I don't think that any I have referred* [to respiratory or cardiovascular] *have been seen yet. …* [CGP03]

Variation in models of access for rehabilitation led PwLC to question the equity of care across health boards as they were aware of Long COVID services provided in other parts of Scotland and in England. Some PwLC who were able to afford it sought private health care and experienced improvements in their condition.

> *I've taken it upon myself to see a physio privately who is helping me because that's the hardest thing is this pacing business…Because I do too much one day, then I can't do anything the following day, maybe even two days…It's been very*

> *helpful because she, she's guiding me because what I would I think is manageable and doable is very different to what she says is manageable and doable.* [DPwLC01]

People with Long COVID emphasised the need for a more joined-up service to improve communication and coordination of care.

> *I should have had the results in six weeks, but I've not had them, I don't know who to contact to get them, because it was through the neurologist rather than my GP.* [APwLC03]

They also felt that specialists focused only on specific aspects of their health but not the condition as a whole.

> *I just feel sometimes having that one person, like I know a lot of people have a consultant that they go to and that's the person that they speak to, or the centre that they go to, for support. There's a group of people perhaps that they deal with, but they get it. They're a familiar face and that makes sense. But you feel a bit of a pariah, to be honest, with Long COVID.* [BPwLC06]

GPs acknowledged the importance of a more holistic approach to Long COVID care including psychological as well as physical support. They suggested the need for integrated multidisciplinary management that provides support for a complex range of symptoms.

IV: Perceptions and experiences of Long COVID and its management, including rehabilitation

This theme comprised data on GPs knowledge, attitudes, and perceptions of Long COVID, as well as participants' perceptions and experiences of Long COVID services.

GPs knowledge, attitudes, and perceptions of Long COVID

In health boards where Long COVID management was integrated into existing community services with less clear pathways to rehabilitation (health boards A & B),

For peer review only

there was a perceived lack of demand by GPs for Long COVID-specific services. GPs in this study reported low numbers (1-6 per week) of people presenting to their practices for support with symptoms of Long COVID.

> *Maybe 10-12* [Long COVID patients have presented] *in total. But I don't know whether they're all coming to us. They might be just suffering in silence.* [CGP03]

Patients were commonly reported to be of working age and were perceived to be fit and healthy prior to coronavirus infection. Some GPs also reported that females were more commonly presenting with symptoms associated with Long COVID than males.

Some GPs attributed the low patient numbers to natural resolution and the ability to self-manage on the basis that patients were not reconsulting or requesting further sick lines.

> *I think with time most of their symptoms do seem to ease and pass*. [AGP01]

However, some GPs and most PwLC believed that people with Long COVID were not presenting to primary care because they believed there was no support available for Long COVID.

> *A lot of patients don't necessarily consult because probably they are seeing things in the media and things, you know aware that there aren't particular treatments. So, they just think it's par for the course that they feel like that.* [AGP02]

> *"I stopped contacting the GP because I just feel I'm wasting their time"* [APwLC03]

PwLC expressed some concerns associated with GPs being under pressure and attributed their reduced attendance at GP practices to feeling like they were an additional burden on the healthcare system. They spoke of withholding information related to their symptoms due to an awareness that GPs were time pressured, and expressed concerns associated with ensuring appointments were productive.

> *I know there are things that I haven't raised with a GP because I'm aware that they're time pressured, I've raised about ten symptoms already in my*

For peer review only

consultation with them and I know I've got another three sitting on my list, but I can't bring that into the situation…and I'm potentially sitting on stuff that I should have discussed [BPwLC06]

PwLC also noted their concerns over the lack of specialist knowledge and expressed a sense of apprehension when GPs were unable to demonstrate an appropriate level of knowledge concerning the condition.

But the last time I went to the GP they said, 'we find people with Long COVID know more about it than we do.' And I thought that doesn't really fill you with great enthusiasm. [DPwLC04]

Most GPs acknowledged that Long COVID has impacted people living with the condition and conveyed a need for education associated with recovery and returning to work. They spoke of patients feeling pressured to return to work without appropriate support in place to assist their workplace integration. The need for psychological support was also expressed by both GPs and PwLC. Specifically, peer support was identified as a useful resource for people with Long COVID, where they can be supported by others experiencing similar symptoms.

It's just reassuring to know that you're not alone in this. Misery loves company, and it's good to know that there are other people who have this, because otherwise it would become kind of depressing. And it helps put things in perspective, that you know that as bad as you feel someone else is probably feeling worse. [DPwLC06]

A Minority of GPs did not perceive the need for a specialised Long COVID service. Some GPs referred to a scepticism among their colleagues about Long COVID in general:

It's not only for people, it's also for the medical professionals to believe as well that this is a problem. I think there is still some scepticism among medical professionals as well, still, about this being accepted and treated. [CGP03]

For peer review only

One GP participant described themselves as being slightly cynical about Long COVID and wary of the need for rehabilitation, suggesting that patients should *"wait it out"* and they will get better, likening Long COVID and its impact to 'flu.

> "*I think it's exactly like flu. The same applies in flu. You get lots of people that get it. Most people are not terribly well with it, few people get flu without knowing they've had it. Some people recover quickly, some take a longer time to recover and some die*" [AGP04]

Perceptions and experiences of rehabilitation and other Long COVID services

A range of perceptions regarding Long COVID rehabilitation were held by both participant groups. Some PwLC lacked knowledge of the potential role of rehabilitation professionals in supporting people with Long COVID. This view was shared by some GPs who reported a limited understanding of the role of rehabilitation in the management of Long COVID.

> *Physio, I can't really see much of a role. But that could be my lack of knowledge about it because with the patients I have spoken to it's not really so much of a physical thing, it's not like a particular joint pain as such that they would benefit from a physio. It's more the kind of cognitive aspect, maybe an OT, but I don't know what they would add.* [BGP05]

Some PwLC had enough knowledge of rehabilitation to request referral or refer themselves to rehabilitation services where this was an option. In most cases these participants were on waiting lists and had been for some time. Although we did not record participants' job titles (for those in employment), several participants in this category disclosed during the interviews that they were healthcare professionals, with prior knowledge of, or colleagues working in, rehabilitation services. A third group of PwLC reported a need for Long COVID rehabilitation services in their health board area, suggesting a lack of services and/or their promotion.

For peer review only

The small number of participants who had received rehabilitation for Long COVID (n=3) were generally positive about their experience, reporting benefits from specific professions (e.g., physiotherapy, speech and language therapy) and interventions (e.g., breathing exercises), with information and advice on Long COVID and symptom management, particularly the use of pacing, being highly valued.

> *The biggest help was speaking to Speech and Language... she gave me lots of information that was very interesting, and lots about the biology of what's going on with my* [laryngeal] *spasms.* [DPwLC04]

> *Just having somebody to help you manage what that should look like, what is too much, because you can read about pacing, you can chat about it online with other people with Long COVID, but trying to get a model that fits for you as an individual is actually really hard without support.* [DPwLC06]

This contrasted with the views of PwLC of generic self-management booklets, which were commonly reported as lacking person-centredness.

> *When I did refer myself to the* [specialist] *team, I got a booklet, a massive booklet through the post, that says this that and the other. But it's such an individual, highly differentiated set of symptoms that any one person can have, just none of it was particularly relevant to me.* [DPwLC01]

One participant had sought informal rehabilitation from a personal trainer but felt that was not helpful as it was too intense, and the trainer did not have the requisite Long COVID knowledge to support patients effectively to avoid rehabilitation being 'too intense.'

> *"Just with having this discussion it's like a wee light bulb moment that I'm having, that I'm thinking I've tried the PT* [personal training]*, it was too intense, threw the towel in. See all that weights and lunges and dah, dah, dah, I thought I haven't got the energy for it. So maybe physio, as you're saying, if they know all*

For peer review only

about the disease, they know about what they would recommend and maybe be a bit more gentle" [DPwLC01]

Despite the lack of knowledge of the potential role of rehabilitation demonstrated by some GPs, most reported that they would engage with a dedicated Long COVID rehabilitation service, as it would provide an onward referral route for patients, particularly as they commonly reported being limited by secondary care referral criteria. Several GPs felt that a multidisciplinary team approach could be beneficial for their patients and could provide the validation that patients needed.

I think they feel quite isolated actually and I think it would be useful even if objectively…there's not a huge improvement. I think psychologically it would be really important for them. Someone to believe them, to see what's happening, and just thinking someone's looking out for them. [BGP01]

Some also believed that earlier pulmonary rehabilitation could contribute to better functional recovery.

PwLC reported accessing a range of other Long COVID services and sources of support. For some, a helpful source of advice and support came from occupational health services when they were returning to work after a period of Long COVID-related absence. Some PwLC had accessed support from a charity, with some finding it useful and others finding it too generic for their needs. Some participants found support from peers with similar experience of Long COVID was helpful and provided validation. For some this positive peer support was accessed via social media. Others reported negative experiences of social media including finding social media platforms unsafe and unhelpful for sharing lived experiences. The lack of monitoring in online forums contributed to PwLC feeling vulnerable to receiving incorrect information and negativity from others. Many PwLC reported that they resorted to online information, with some taking this information to their GP consultation. Finally, as reported above, some turned to private healthcare in

For peer review only

response to access issues and long waiting times, including psychological therapy, GP, medical specialties, and physiotherapy.

DISCUSSION

We explored the issue of access to community rehabilitation for Long COVID from the perspectives of PwLC and GPs in four Scottish health boards. PwLC described a range of symptoms affecting their physical and mental health and having a profoundly negative impact on their daily life, employment, and relationships. We found a tension between PwLC seeking diagnosis and onward referral and GPs lacking diagnostic tests and limited options for managing Long COVID. This tension could manifest itself in PwLC perceiving themselves as socially stigmatised by their ‘invisible’ condition. We also identified several systemic challenges for Long COVID service delivery which related to access, siloed services, limited resources, and a perceived lack of holistic care, causing frustration for both GPs and PwLC. We found that although a minority of GPs expressed scepticism about Long COVID and the need for rehabilitation and other services for this patient group, there was general agreement between PwLC and GPs on the need for accessible, person-centred services and support. Regarding community rehabilitation, we found that: i) some PwLC and some GPs lacked knowledge on the potential role of community rehabilitation in the management of Long COVID; ii) having prior knowledge of rehabilitation or being a healthcare professional appeared to facilitate access to community rehabilitation, and iii) PwLC who had received rehabilitation generally found it beneficial. Due to the lack of knowledge and difficulty accessing rehabilitation however, PwLC had accessed a range of other services and sources of support, with varying success.

The relationship between Long COVID and reduction in physical and mental health outcomes and quality of life is becoming increasingly known [29 30]. A large Scottish population cohort study found that almost 50% of PwLC had not recovered 6-18 months after symptomatic infection, and that PwLC experienced a wide range of symptoms affecting daily activities and quality of life [31]. This study adds to that growing

For peer review only

evidence-base and demonstrates the influence of the condition on a sample of PwLC living in Scotland almost 3-years on from the start of the pandemic. The persisting prevalence and impact of Long COVID on people's lives further emphasises the need for support and services such as community rehabilitation to be available, in keeping with recommendations [1 6 8].

These recommendations are that PwLC should have access to personalised and multidisciplinary rehabilitation; such rehabilitation is reportedly available throughout Scotland [12] delivered by a variety of service models. Our previous research, however, observed low numbers of PwLC receiving community rehabilitation in some areas of Scotland [15]. This study has shed some light on the potential reasons for the mismatch between recommendations, reported service availability and numbers of PwLC accessing community rehabilitation.

Lack of GP knowledge regarding community rehabilitation and its potential role in Long COVID may, in part, be attributed to the nature of Long COVID as a new condition that health professionals are still learning how to manage. Previous research has reported a lack of GP understanding of the role of rehabilitation professionals in the management of conditions commonly encountered in primary care; for example, the role of physiotherapists in osteoarthritis management [32]. Furthermore, international public understanding of the role of occupational therapy has been reported as limited [33]. Therefore, in the context of a new condition with an evolving evidence-base, it is perhaps not surprising that GPs and PwLC may have limited understanding of what rehabilitation professionals can offer to this patient population. Furthermore, the reluctance to promote the availability of services in some areas, due to pre-existing resourcing and anticipated demand-capacity issues may have further limited access to Long COVID rehabilitation [15]. Issues with promotion of services, clarity of pathways and interdisciplinary communication between GPs and rehabilitation professionals have been reported previously[32 34]. The findings of this study suggest that further improvements in communication and collaborative working may be required to enhance

For peer review only

access to community rehabilitation for PwLC [32]. Indeed, the finding that prior knowledge of rehabilitation, and being a healthcare professional facilitated access to community rehabilitation for PwLC, is further evidence that successfully navigating the referral system is challenging.

The findings of PwLC who had managed to access community rehabilitation being satisfied with it, and GPs wanting a Long COVID service to refer patients to are in keeping with previous research [17]. The challenge not only lies in availability of such services, but clearly in PwLC and GPs awareness of the benefits of these services, their active promotion, and clear and timely accessibility of rehabilitation services. There is therefore a need to overcome the systemic challenges to accessing timely rehabilitation reported in this study. Considering the ongoing nature of living with Long COVID, these challenges are likely to continue beyond the time and resource constraints of Government funding provision for existing Long COVID rehabilitation within rehabilitation services that were already historically underfunded and considered 'Cinderella services' [15].

Strengths and limitations

This was the first study to explore the issue of accessing community rehabilitation in the Scottish context and included the perspectives of those referring and potentially being referred to community rehabilitation. One researcher conducted all interviews to ensure consistency and multiple researchers were involved in analysing and interpreting the data, including people with lived experience of Long COVID. There are, however, some limitations. As this study was an extension of our original study, we had insufficient resources to enable larger participant samples. We realised from the outset that we would be unable to describe achieving data saturation. Although we recruited to target for the GP sample, there was under-representation from the two health boards with dedicated Long COVID services (one newly launched and one halted). Therefore, the data largely represents the views of GPs from health boards where Long COVID services were integrated into existing community rehabilitation services; it is possible that GPs

For peer review only

views of dedicated Long COVID services may be different. We recruited a small, mostly female sample of PwLC, and mostly from health boards with integrated or a halted dedicated Long COVID service. Their views on accessing Long COVID services may therefore have been biased. Recruitment of PwLC was likely limited by our reliance on social media and one Long COVID charity; however, both mechanisms had the potential to reach many people. Despite these challenges, both the PwLC and GP participant groups provided consistent understandings of the challenges they faced when dealing with Long COVID. Therefore, while data saturation is not claimed, it does appear that we achieved adequate data sufficiency to be confident that the findings reflect key issues within each participant group.

Implications for practice and research

There is a need for greater understanding by the public, GPs, and other potential referrers of the role of community rehabilitation professionals in the management of Long COVID. There is an equally important need for community rehabilitation services to be well promoted and accessible to the PwLC for whom they may be appropriate. Long COVID is still a prevalent condition whose impact on individuals can be profound. The need for community rehabilitation for PwLC is likely to persist. Service providers should therefore consider availability and accessibility of Long COVID rehabilitation and ensure adequate interprofessional communication and collaboration to enhance the experience for PwLC.

CONCLUSION

We have provided further understanding of the barriers to accessing Long COVID community rehabilitation by exploring the perceptions and experiences of key stakeholders in the referral process. These findings can be used by those (re)designing community rehabilitation services for people with Long COVID and potentially for other conditions. There remains a need for greater public and GP awareness of the role of rehabilitation professionals in Long COVID.

For peer review only

AUTHOR CONTRIBUTIONS

KC, ED, JM, LA, JP, JO, AL, PS contributed to the study's conception and design. EHW undertook data collection. ED, KC, EHW, TT, JC, JS, ES undertook analyses and drafted the first version of the manuscript. KC led future iterations of the manuscript. All authors read and commented on the manuscript and approved the final version of it. The corresponding author attests that all authors meet authorship criteria and that nobody meeting the criteria have been omitted. ED and KC have joint responsibility for the research conduct of the study, had access to the data, and controlled the decision to publish.

COMPETING INTERESTS

All authors have completed the ICMJE uniform disclosure form at www.icmje.org/coi_disclosure.pdf and declare: all authors had financial support from the Chief Scientist Office Scotland (grant number COV/LTE/20/29) for the submitted work; ED has received research grants from NIHR and Scottish Government for the following studies: Caring for Long COVID in primary care and DBI COVID study respectively. JM has received funding for LOCOMOTION (Long COVID multidisciplinary consortium: optimising treatments and services across the NHS). No other relationships or activities that could appear to have influenced the submitted work are declared by any of the authors.

FUNDING STATEMENT

This work was supported by the Chief Scientist Office Scotland, grant number COV/LTE/20/29

DATA AVAILABILITY

All data produced in the present study are available upon reasonable request. Robert Gordon University holds the copyright for the full interview transcripts and may grant data sharing permission on request.

For peer review only

688 **ETHICS APPROVAL**

689 Ethical approval was granted from the Wales Research Ethics Committee 6 [21/WA/0118
690 A03], and each of the four Health Boards granted R&D management approval.

691 **ACKNOWLEDGEMENTS**

692 We are grateful to all our participants who gave of their time to take part in interviews.
693 We would like to thank Amanda Cardy, Northeast coordinator NRS Primary Care Network
694 and local principal investigators Lynne Frew, Lynn Morrison, and Gail Thomson-Patel for
695 support with participant recruitment.

For peer review only

REFERENCES

1. NICE. COVID-19 rapid guideline: managing the longterm effects of COVID-19, 2022.
2. World Health Organization. With the international public health emergency ending, WHO/Europe launches its transition plan for COVID-19. 2023 [Available from: https://www.who.int/europe/news/item/12-06-2023-with-the-international-public-health-emergency-ending--who-europe-launches-its-transition-plan-for-covid-19 accessed 5 October 2023.
3. Office for National Statistics (ONS). Prevalence of ongoing symptoms following coronavirus (COVID-19) infection in the UK: 30 March 2023. 2023 [Available from: https://www.ons.gov.uk/peoplepopulationandcommunity/healthandsocialcare/conditionsanddiseases/bulletins/prevalenceofongoingsymptomsfollowingcoronaviruscovid19infectionintheuk/30march2023 accessed 5 October 2023.
4. Davis HE, McCorkell L, Vogel JM, et al. Long COVID: major findings, mechanisms and recommendations. *Nat Rev Microbiol* 2023;21(3):133-46. doi: 10.1038/s41579-022-00846-2 [published Online First: 20230113]
5. O'Hare AM, Vig EK, Iwashyna TJ, et al. Complexity and Challenges of the Clinical Diagnosis and Management of Long COVID. *JAMA Netw Open* 2022;5(11):e2240332. doi: 10.1001/jamanetworkopen.2022.40332 [published Online First: 20221101]
6. Heeney A, Connolly SP, Dillon R, et al. Post-COVID care delivery: The experience from an Irish tertiary centre's post-COVID clinic. *PLoS One* 2023;18(8):e0289245. doi: 10.1371/journal.pone.0289245 [published Online First: 20230811]
7. DeMars J, Brown DA, Angelidis I, et al. What is Safe Long COVID Rehabilitation? *J Occup Rehabil* 2023;33(2):227-30. doi: 10.1007/s10926-022-10075-2
8. Decary S, De Groote W, Arienti C, et al. Scoping review of rehabilitation care models for post COVID-19 condition. *Bull World Health Organ* 2022;100(11):676-88. doi: 10.2471/BLT.22.288105 [published Online First: 20221003]
9. Daynes E, Baldwin M, Greening NJ, et al. The effect of COVID rehabilitation for ongoing symptoms Post HOSPitalisation with COVID-19 (PHOSP-R): protocol for a randomised parallel group controlled trial on behalf of the PHOSP consortium. *Trials* 2023;24(1):61. doi: 10.1186/s13063-023-07093-7 [published Online First: 20230126]
10. Murray E, Goodfellow H, Bindman J, et al. Development, deployment and evaluation of digitally enabled, remote, supported rehabilitation for people with long COVID-19 (Living With COVID-19 Recovery): protocol for a mixed-methods study. *BMJ Open* 2022;12(2):e057408. doi: 10.1136/bmjopen-2021-057408 [published Online First: 20220207]
11. Dean E. What happens inside a long covid clinic? *BMJ* 2023;382:p1791. doi: 10.1136/bmj.p1791
12. Duncan E, Cooper K, Cowie J, et al. A national survey of community rehabilitation service provision for people with long Covid in Scotland. *F1000Res* 2020;9:1416. doi: 10.12688/f1000research.27894.2 [published Online First: 20201207]
13. Baz SA, Fang C, Carpentieri JD, et al. 'I don't know what to do or where to go'. Experiences of accessing healthcare support from the perspectives of people living with Long Covid and healthcare professionals: A qualitative study in Bradford, UK. *Health Expect* 2023;26(1):542-54. doi: 10.1111/hex.13687 [published Online First: 20221213]
14. Ladds E, Rushforth A, Wieringa S, et al. Persistent symptoms after Covid-19: qualitative study of 114 "long Covid" patients and draft quality principles for services. *BMC Health Serv Res* 2020;20(1):1144. doi: 10.1186/s12913-020-06001-y [published Online First: 20201220]
15. Duncan E, Alexander L, Cowie J, et al. Perceptions and experiences of different Long COVID community rehabilitation service models from the perspectives of people living with Long COVID and healthcare professionals. MedRxiv 20230930 [Pre-Print] September 28 2023. https://doi.org/10.1101/2023.08.23.23293876.

For peer review only

16. Kingstone T, Taylor AK, O'Donnell CA, et al. Finding the 'right' GP: a qualitative study of the experiences of people with long-COVID. *BJGP Open* 2020;4(5) doi: 10.3399/bjgpopen20X101143 [published Online First: 20201215]
17. Brennan A, Broughan J, McCombe G, et al. Enhancing the management of long COVID in general practice: a scoping review. *BJGP Open* 2022;6(3) doi: 10.3399/BJGPO.2021.0178 [published Online First: 20220928]
18. Thomas C, Faghy MA, Owen R, et al. Lived experience of patients with Long COVID: a qualitative study in the UK. *BMJ Open* 2023;13(4):e068481. doi: 10.1136/bmjopen-2022-068481 [published Online First: 20230426]
19. Skilbeck L, Spanton C, Paton M. Patients' lived experience and reflections on long COVID: an interpretive phenomenological analysis within an integrated adult primary care psychology NHS service. *J Patient Rep Outcomes* 2023;7(1):30. doi: 10.1186/s41687-023-00570-2 [published Online First: 20230320]
20. Bachmeier BE, Holzle S, Gasser M, et al. How do German General Practitioners Manage Long-/Post-COVID? A Qualitative Study in Primary Care. *Viruses* 2023;15(4) doi: 10.3390/v15041016 [published Online First: 20230420]
21. World Health Organization. Rehabilitation. 2023 [Available from: https://www.who.int/news-room/fact-sheets/detail/rehabilitation accessed 28 September 2023.
22. NICE. COVID-19 rapid guideline: managing the longterm effects of COVID-19., 2022.
23. Tong A, Sainsbury P, Craig J. Consolidated criteria for reporting qualitative research (COREQ): a 32-item checklist for interviews and focus groups. *Int J Qual Health Care* 2007;19(6):349-57. doi: 10.1093/intqhc/mzm042 [published Online First: 20070914]
24. Scottish Government. Scottish Government Urban Rural Classification 2020. 2022 [Available from: https://www.gov.scot/publications/scottish-government-urban-rural-classification-2020/ accessed 05 October 2023.
25. Halcomb EJ, Davidson PM. Is verbatim transcription of interview data always necessary? *Appl Nurs Res* 2006;19(1):38-42. doi: 10.1016/j.apnr.2005.06.001
26. Wellard S, McKenna L. Turning tapes into text: issues surrounding the transcription of interviews. *Contemp Nurse* 2001;11(2-3):180-6. doi: 10.5172/conu.11.2-3.180
27. Gale NK, Heath G, Cameron E, et al. Using the framework method for the analysis of qualitative data in multi-disciplinary health research. *BMC Med Res Methodol* 2013;13:117. doi: 10.1186/1471-2288-13-117 [published Online First: 20130918]
28. Goldsmith LJ. Using Framework Analysis in Applied Qualitative Research. 2021
29. Kennelly CE, Nguyen ATP, Sheikhan NY, et al. The lived experience of long COVID: A qualitative study of mental health, quality of life, and coping. *PLoS One* 2023;18(10):e0292630. doi: 10.1371/journal.pone.0292630 [published Online First: 20231013]
30. Owen R, Ashton RE, Skipper L, et al. Long COVID quality of life and healthcare experiences in the UK: a mixed method online survey. *Qual Life Res* 2023 doi: 10.1007/s11136-023-03513-y [published Online First: 20230922]
31. Hastie CE, Lowe DJ, McAuley A, et al. Outcomes among confirmed cases and a matched comparison group in the Long-COVID in Scotland study. *Nat Commun* 2022;13(1):5663. doi: 10.1038/s41467-022-33415-5 [published Online First: 20221012]
32. Okwera A BscPt M, May S Ma GDPFMP. Views of general practitioners toward physiotherapy management of osteoarthritis-a qualitative study. *Physiother Theory Pract* 2019;35(10):940-46. doi: 10.1080/09593985.2018.1459987 [published Online First: 20180416]
33. Rahja M, Laver K. What does the Australian public know about occupational therapy for older people? A population survey. *Aust Occup Ther J* 2019;66(4):511-18. doi: 10.1111/1440-1630.12578 [published Online First: 20190410]
34. Igwesi-Chidobe CN, Bishop A, Humphreys K, et al. Implementing patient direct access to musculoskeletal physiotherapy in primary care: views of patients, general practitioners,

795 physiotherapists and clinical commissioners in England. *Physiotherapy* 2021;111:31-39. doi: 10.1016/j.physio.2020.07.002 [published Online First: 20200711]

For peer review only

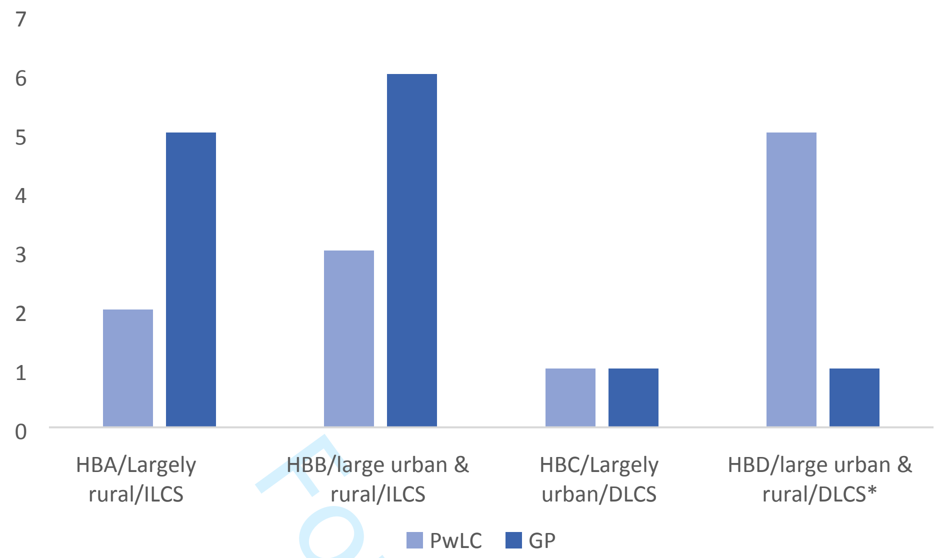

Key: HB=Health Board; ILCS=Integrated Long COVID Service; DLCS=Dedicated Long COVID Service; *=Dedicated Long COVID Service temporarily halted due to staffing & funding; PwLC=People with Long COVID; GP=General Practitioners

Figure 1: Number of GPs and People with Long COVID recruited from each health board

For peer review only

IRAS ID: 295672

Protocol

1.0 Title

Evaluating long-covid rehabilitation in Scotland

2.0 Summary

Studies estimate that 10–35% of people with COVID-19 experience disabling clinical symptoms following the acute phase of their illness (long-covid). Given the prevalence of COVID-19 in Scotland, there is assumed to be a significant number of people experiencing long-covid, and they are starting to be referred to rehabilitation specialists. Community rehabilitation, delivered by physiotherapists, occupational therapists and other health professionals, is a well-established approach to enabling people to maximise their quality of life and recovery. However, community rehabilitation for people with long-covid is in its infancy. There are considerable differences in how community rehabilitation for people with long-covid is currently delivered across Scotland. We want to assess which models of community rehabilitation are most appropriate, in which circumstances. We will evaluate the delivery and outcomes of four different models of community rehabilitation for people with long-covid currently being delivered in Scotland. This will help us to identify which models are more suitable, for which patient groups and in which contexts. We will then hold online workshops with community rehabilitation managers, service leads and others from across Scotland. During the workshops, we will present our findings and support participants to develop evidence-based action plans to improve their local long-covid community rehabilitation services

3.0 Background

3.1 Introduction

This study is an in-depth exploration of models being used to deliver rehabilitation for longer-term effects of COVID-19 in Scotland. Understanding and evaluating how service models are developing, in parallel with evolving knowledge of the impact of COVID-19 on patients, will inform the development of new and responsive patient-centred models that can effectively meet patient and service need. This work is vital. As evidence emerges on the epidemiology and consequences of COVID-19, its impact on individuals and society, and potential burden on health services (1), strategies are required to address the long-term effects of COVID-19. The Scottish Government's Framework for Supporting People through Recovery and Rehabilitation during and after the COVID-19 Pandemic (2) states that "everyone with rehabilitation needs…will be able to access the care and support they need to live well, on their own terms." It also highlights the important role of digital solutions. This research will develop implementable recommendations for post-COVID-19 rehabilitation, congruent with the principles of the Scottish Rehabilitation Framework and Digital Health and Care Strategy (3), and that meet patients' needs.

For peer review only - http://bmjopen.bmj.com/site/about/guidelines.xhtml

3.2: Long-covid

Long-term effects of COVID-19, or "long-covid" has received much recent media attention with action groups calling for more support (4). With no agreed definition, we will use that proposed by Greenhalgh et al (5) of symptoms extending 3-weeks beyond onset (post-acute COVID-19) and 12-weeks beyond onset (chronic COVID-19). Studies on long-term consequences of COVID-19 infection are ongoing; however, it is estimated that 10 – 35% of patients may experience post-acute COVID-19 (5-7). Based on Scottish Government figures for positive COVID-19 tests and survival to date (28th February 2021) this equates to between 19,367 and 67,784 people in Scotland with post-acute COVID-19, with around 2,000 remaining significantly unwell at 12-weeks, commonly due to organ damage (7). Long-covid also affects people with milder symptoms and those who were not hospitalised (6). Rehabilitation therefore needs to be accessible to those presenting in community settings as well as being discharged from hospital.

Reported symptoms vary widely and commonly include respiratory, cardiopulmonary, neurological, musculoskeletal and mental wellbeing sequelae, as well as fatigue and loss of taste and smell (5,6,8). As described above, presentation and severity are variable, and it is important to note that many patients with long-covid will have co-existing conditions that will impact on symptoms and require dual management. Rehabilitation therefore needs to be multidisciplinary, comprehensive, and tailored to patients' needs, in order to maximise function, quality of life and participation in society (9).

3.3: Long-covid rehabilitation

Rehabilitation for long-covid is in its infancy, reflected in a recent living systematic review update finding predominantly expert opinion, indicating that high-quality research is required (10). Research on rehabilitation for other respiratory conditions *may* be generalisable to the COVID-19 population (11), but this is yet to be determined. Several UK expert bodies and published expert opinion recommend a stepped, needs-based rehabilitation approach incorporating information provision, self-management support and specialist services as required. They also recommend that rehabilitation should be individualised and progressive and the use of digital solutions optimised. *How* rehabilitation can be optimally delivered is not known but vital to determine, given that rehabilitation services need to cope with additional COVID-19 demand whilst continuing to provide rehabilitation for other, often vulnerable, patient populations (12). By investigating different ***models of community rehabilitation*** currently implemented in Scotland to evaluate what works, where, with whom and how, we will develop recommendations on *how* rehabilitation can be best delivered across Scotland to meet the needs of patients and services, thereby developing new responsive models of rehabilitation.

4.0 Aims & research questions

The overarching aim of this research is to provide responsive evidence-based

For peer review only - http://bmjopen.bmj.com/site/about/guidelines.xhtml

recommendations to the NHS about how to most effectively deliver community rehabilitation for people with long-covid. We will do this by evaluating the implementation and impact of different emerging models of long-covid rehabilitation in different contexts in Scotland. We will address the following specific research questions:

1. How is rehabilitation for long-covid being delivered in 4 Scottish health boards?
2. What are the barriers and facilitators to implementing long-covid rehabilitation from managerial, rehabilitation staff, and patients' perspectives and experiences?
3. What are the perceptions and experiences of community-dwelling adults with long-covid on accessing rehabilitation for long-covid?
4. What are GPs perceptions and experiences of managing community dwelling adults presenting with symptoms of long-covid that may be suitable for rehabilitation?
5. What are the outcomes of different community rehabilitation models for people experiencing long-covid?
6. What are the costs associated with long-covid rehabilitation in the 4 selected cases?
7. In what ways are the selected long-covid community rehabilitation models successful (or not)?
8. How can we use the knowledge from 1-5 above to inform the development of responsive patient-centred models of rehabilitation that can be adapted to suit the needs of patients and services across Scotland?

5.0 Methodology & methods

We will conduct a mixed-methods case study realist evaluation (RE) of different models of community rehabilitation, in different contextual settings, for people experiencing long-covid in order to understand what works, for whom, in what circumstances and how. The study is informed by the RE framework (13), which is used to study the process and context of 'implementation' and to identify the 'mechanisms of action' that lead to successful (or unsuccessful) delivery. It will also be used to investigate how interventions are being implemented in each case study area. Understanding the local contexts and mechanisms of action will enable us to explain observed outcomes in the different models of community rehabilitation delivery for people with long-covid. Realist Evaluation is a highly appropriate method to use to study the delivery and impact of emerging models of care for long-covid. Realist evaluation emerged in response to the need for knowledge that extends beyond that obtained by controlled trial design (is the intervention effective?) to answering questions of what works for whom and in what circumstances. It is 'realist' in the sense that it does not deny the relevance of robust measurement of outcomes, but also seeks to take account of the context in which interventions are delivered and to identify the mechanisms that make programmes work (or not) to produce the observed outcomes. It is not the interventions themselves that work but the ability and willingness of participants to engage with them.

For peer review only - http://bmjopen.bmj.com/site/about/guidelines.xhtml

IRAS ID: 295672

Setting

Rehabilitation services in four Scottish Health Boards: NHS Ayrshire & Arran, NHS Grampian, NHS Lanarkshire, NHS Tayside. The boards have been selected to represent a diversity of models of rehabilitation in terms of mode of delivery (digital/face-to-face/blended/self-management) and whether they provide a new long-covid service or integrate long-covid rehabilitation into existing services. This information was gained by a Scottish-wide survey of long-covid rehabilitation conducted by the study team (14). The selected NHS boards also represent a range of geographical areas in relation to urban/rural classification, to allow for transferability of findings across Scotland, and boards with sufficient covid-19 cases to make recruitment viable.

The study consists of 2 work packages (WP):

WP1: Case study RE to measure implementation and outcomes of different service models of community rehabilitation for people experiencing long-covid. Within WP1 we will conduct:

I. Process evaluation addressing research questions 1-4

Data will be collected at 4 time-points over a 12-month period across each case to understand local contexts and decisions regarding delivery of community rehabilitation for people with long-covid. Data collection rounds 1 to 4 will describe the long-covid models of community rehabilitation and track local decisions on how services are implementing and developing their long-covid rehabilitation service. Data collected during rounds 1 to 4 will elicit rationales for service delivery decision making and how implementation is supposed to work. Data collection will be obtained from online focus groups (with people who have received long-covid community rehabilitation), and online individual interviews (6-10 per case per round) with managers/service leads/consultants/ and rehabilitation staff with potential to deliver long-covid community rehabilitation. Data from Round 1 will specifically be used to elicit rationales regarding local decisions, identify 'folk theories' surrounding how community rehabilitation delivery is supposed to work, and identify hypothesised mechanisms of (successful) change/implementation. This will establish the locally hypothesised context-mechanism-outcome (CMO) configurations which will then be tested using data gathered in Rounds 2-4.

Participants

1. People who have received long-covid rehabilitation (patients), managers/service leads, and professionals delivering long-covid rehabilitation will be recruited. See table 1 for inclusion/exclusion criteria.
2. Community dwelling adults with long-covid (i.e., not currently receiving community rehabilitation) and GPs in each of the four health boards.

Recruitment

Patients will be sent a study pack in one of the following ways: (i) with their long-covid appointment if it is mailed out to them; in-person from their rehabilitation professional when they receive their first session (either in their home or in a community rehabilitation setting); by mail if their initial session is held virtually. The study pack will consist of: i) letter of invitation, ii) participant information sheet, iii) baseline measures. Patients will therefore be identified by administrative or clinical staff, and will opt-in to the study by completing the

For peer review only - http://bmjopen.bmj.com/site/about/guidelines.xhtml

IRAS ID: 295672

baseline measures (paper-based or online) and returning them to the research team. Participants will opt-in to taking part in a focus group, and if they wish to do so will provide contact details for the study team to contact them.

Table 1: Inclusion/exclusion criteria participants

	**Inclusion**	**Exclusion**
**Patients**	-Aged 18+<br>-COVID-19 diagnosis (with or without positive covid-19 test) >3-weeks previously<br>-Referred due to presenting with symptoms thought to be due to long-covid (including but not limited to: respiratory, fatigue, musculoskeletal, mental health)<br>-Suitable for rehabilitation (determined by rehabilitation staff)<br>-Able to understand written & spoken English<br>-Willing to take part in study	-Aged <18<br>-Requiring specialist (inpatient) management of specific complications<br>-Unable or unwilling to provide informed consent<br>-Other life-limiting illness with life expectancy <6-months e.g. disseminated malignancy
**Managers/service leads**	Managers/service leads of community rehabilitation services providing long-covid rehabilitation	Managers/service leads of inpatient services
**Rehabilitation professionals**	Healthcare professionals delivering long-covid rehabilitation including but not limited to, physiotherapists, occupational therapists, speech and language therapists, psychologists, medical doctors. Where relevant (i.e. involved in delivering long-covid rehabilitation) healthcare support workers will be included	-Student healthcare professionals<br>-Healthcare professionals providing in-patient rehabilitation
**Community dwelling adults with long-covid**	Aged 18+<br>Self-identify as having experience of long-covid and accessing/attempting to access healthcare services for possible rehabilitation	-Aged <18<br>-Unable or unwilling to provide informed consent<br>-Other life-limiting illness with life expectancy <6-months e.g., disseminated malignancy
**GPs**	GP based in any of the four participating health boards with experience of patients with probable long-covid who	

For peer review only - http://bmjopen.bmj.com/site/about/guidelines.xhtml

	may be suitable for rehabilitation	

Rehabilitation professionals and Managers/service leads will be recruited by email with a letter of invitation and participant information sheet attached. The email will come from a relevant gatekeeper e.g. team lead/manager for rehabilitation professionals; sector lead/allied health professions lead for service leads/managers. Participants will opt-in to the study by contacting the research team by email or telephone.

Community dwelling adults with long-covid will be recruited via social media using RGU School of health sciences and NMAHP research unit University of Stirling Facebook and Twitter accounts, via long-COVID Scotland action group, and The Alliance. Following these routes, we anticipate snowball sampling will occur.
GPs will be recruited by email invitation circulated on our behalf by the NRS Primary Care Network**.**
We plan to recruit these two groups of participants for one round of data collection only, anticipating that referral to long-COVID services will pick up in the coming months. However, should this assumption be incorrect, and referrals to services and this study remain low, we will include these two groups in subsequent rounds of data collection also.

Informed consent
Patients returning baseline measures will indicate their consent to taking part in the quantitative data collection by answering a question on the baseline measures paper or electronic form. Participants will self-select to take part in the focus group by leaving their name and contact details at the end of the survey or e-mailing the research team directly. The research fellow (RF) will then contact the participant by telephone or Microsoft Teams to discuss further and if relevant make arrangements for focus group participation. Consent to take part in the focus group will be provided verbally (audio recorded) during a separate telephone or Microsoft Teams call with the RF. A consent form with standard statements will be read to participants and they will have to agree to each statement in order to take part. Rehabilitation professionals and service leads/managers will provide verbal consent prior to commencing their interviews in the same manner.

Community-dwelling adults with long-covid will contact the research team using the details provided in invitation materials. Individuals will then be provided with study information and following discussion with the RF will provide verbal consent to be interviewed as described above.

GPs will likewise contact the research team following invitation emails sent by the NRS Primary Care Research Network, and will likewise provide verbal informed consent.

Data collection

Patients: One online focus group will be conducted in each case study site during each round of data collection (n=4 focus groups of 8-10 participants each). Patients will be purposively sampled from those opting-in to a focus

For peer review only - http://bmjopen.bmj.com/site/about/guidelines.xhtml

group, based on sampling criteria including age, gender, ethnicity, deprivation, and whether they completed rehabilitation or not. In the event that sufficient diversity is not achieved by this method, rehabilitation services will be asked to conduct additional targeted recruitment on our behalf. Focus groups will take place via Microsoft Teams and will each be conducted by 2 trained researchers using a topic guide. Focus groups will last no longer than 2-hours, will include adequate rest breaks, and will be recorded via Teams. To address issues of digital exclusion, a sample of participants who are interested in taking part but unable (or unwilling) to join an online focus group will be invited to provide their views via telephone interview or a short paper-based survey.

Rehabilitation professionals and service leads/managers will take part in a 1-1 interview, conducted via Teams, supported by a topic guide and recorded in the same manner as the focus groups. From those volunteering to take part we will purposively recruit 3-5 professionals and 2 managers per site to be theoretically representative of staff at each site. We anticipate that the same staff will take part in each of the 4 rounds of data collection, unless there are changes in staffing, in which case additional recruitment will be conducted.

Community dwelling adults and GPs will take part in a single 1-1 interview, conducted by Teams, supported by a topic guide and recorded in the same manner as the focus groups. We will aim to recruit 3-5 community dwelling adults and 2-5 GPs at each site.

Data processing

Following data collection, the MP3 recordings will be downloaded by the RF and saved on secure servers at Robert Gordon University (RGU) and Stirling University (UoS) only accessible to the research team. The transcripts will be transcribed by GDPR-compliant transcribers and stored on the same secure servers.

Data analysis

Data will be analysed to articulate the context and mechanisms of action and relate these to observed (quantitative and qualitative) outcomes (CMO configurations). Each data source will be analysed individually in the first instance to reach separate conclusions. Data from meetings and interviews will be analysed using the Framework Approach (15) aided by NVivo (V12) software. For all qualitative datasets, 10% of transcripts will be coded independently by two RFs and the resulting codes discussed, refined and agreed as a final coding frame to be systematically applied to all transcripts. Following familiarisation with the data, a thematic framework will be developed and applied across the data set. Data will then be tabulated and conceptual maps used to make links between themes. Data will be used to construct case summaries at the level of the different modes of delivery and across different sites. All data for each mode of delivery and for each delivery site will be collected together and consistencies/ inconsistencies searched for. The aim of analysis at this stage is to identify the core barriers and facilitators and mechanisms of action within each mode of delivery, the detailed explanations for them and interactions between them.

For peer review only - http://bmjopen.bmj.com/site/about/guidelines.xhtml

IRAS ID: 295672

II. Outcome evaluation and cost-consequence analysis addressing outcomes 5-7

Outcomes data will be collected before and after people receive community rehabilitation in each location/model of service delivery to establish whether improvements are observable. Recruitment of up to 100 people per case study site will be conducted, from month 3 to month 18 of the study, by the staff delivering community rehabilitation in each local site. It is anticipated that each staff member (approximately 4-6 per team) will collect data on between 15-20 people over the 16-month period. The following patient demographic and clinical data will be collected by each team member for each consenting patient:

- Age
- Sex
- Ethnicity
- Occupation
- Social deprivation status in deciles (gathered using home postcode entered into https://simd.scot/#/simd2020/BTTTFTT/14/-2.7960/55.9548/
- Key presenting problems (e.g. musculoskeletal, fatigue, breathlessness, mental health)
- Co-morbidities (e.g. diabetes, hypertension, COPD)
- Smoking status
- Height & Weight (to estimate BMI)
- Duration of rehabilitation (date of first appointment to discharge)
- Number and duration of sessions
- Number and type of professionals seen

Patient outcomes will be collected at baseline immediately prior to first appointment, at the end of the last session of rehabilitation, and at 3-month follow-up. Baseline measures will be provided to patients with their appointment letter or at their first rehabilitation session (in person or by post if session is virtual), and they can complete the paper copy and return to the study team in freepost envelope or they can complete the measures online (Jisc online surveys) as a web address will also be provided. Each participant will have a unique ID allocated by the site administration or clinical staff, which will be documented on the measures. Patients will be instructed to input this unique ID if they complete the measures online. Reminders will be sent by administration/clinical staff to follow-up individuals who do not submit completed measures. Regular communication between the study RFs and case study sites will facilitate reminders and sending discharge ad follow-up measures to participants.

Outcome measures

In keeping with the PHOSP study[1], we include the following outcomes:

[1]http://www.isrctn.com/ISRCTN10980107

For peer review only - http://bmjopen.bmj.com/site/about/guidelines.xhtml

IRAS ID: 295672

Primary Outcome

Quality of Life: EQ-5D-5L utility index and visual analogue scale (VAS).

Secondary Outcomes

Mental Health

- Generalised Anxiety Disorder Assessment (GAD-7)
- Patient Health Questionnaire (PHQ-9)
- Post-Traumatic Stress Disorder Checklist for DSM-5 (PCL-5)

Clinical symptoms

- Dyspnoea12 Questionnaire
- FACIT – Fatigue Questionnaire

Generic

- Nottingham Extended Activities of Daily Living Scale
- General Practice Physical Activity Scale

Covid Specific

Post-COVID-19 Functional Status (PCFS)

Service-related data

In addition to the data collected from consenting patients, rehabilitation staff will collect the demographic and clinical data listed above (age, ethnicity, occupation etc.) for all long-covid referrals during the study period. This will allow us to determine the extent to which our sample is representative of the long-covid population in the four study sites.

Data analysis

Analysis of quantitative data will be address research aims: 3 (What are the outcomes of different community rehabilitation models for people experiencing long-covid?) and 4 (What are the costs associated with long-covid rehabilitation in the 4 selected cases?) Descriptive statistics will be tabulated for all outcomes at each time point, showing means and SDs. To describe representative changes in each outcome measured, generalized linear mixed models will be conducted. Mixed effects models will be used to account for the repeated measures nature of the data and appropriately address missing data without requirement for listwise deletion. Random intercepts and slopes will be included to quantify inter-individual variability in baseline measures and response to rehabilitation as well as general trends. Where suggested by the data and model checking processes, autoregressive models and non-normal link functions will be included to obtain more accurate parameter estimates and their associated standard errors. Addition of fixed effects into models will be included to investigate systematic differences associated with demographic factors (e.g. age, ethnicity, social deprivation status) and study sites where appropriate statistical power exists. Standard model building (e.g. modelling the effects of time [within-person fluctuation to within-person change over time], modelling the effects of predictors [time-invariant predictors to time-varying predictors in models of

For peer review only - http://bmjopen.bmj.com/site/about/guidelines.xhtml

within-person fluctuation and change]) and model checking (e.g. likelihood ratio tests and residual analyses) processes will be used throughout, with all analyses performed using the lme4 package in the statistical environment R.
The primary outcome is general health status measured via the EQ-5D-5L utility index and VAS. The EQ-5D-5L has been selected to differentiate smaller changes in health status and is less prone to ceiling effects (16). Based on NICE recommendations, quality-adjusted life-years will be calculated using tariffs specific to the EQ-5D-5L and will be mapped for reference-case analyses using the function developed by van Hout (17).
The costs associated with long-covid rehabilitation will be quantified through mean values for all outcomes presented for each study site with uncertainty estimated through boot-strapping procedures. Costs incurred at each site from the perspective of the NHS will be presented and will include estimated costs of the differing modes of delivery (digital, self-management and face to face). Unit costs will be obtained and calculated from Public Health Scotland's Cost Book for outpatient allied health professional clinics (R044X), summary of expenditure on community staff and supplies (R550), and community staff WTE and pay (SFR 8.1). ***Statistical Power:*** Estimates of statistical power were based on the primary outcomes and previous research of the minimum important difference for people with COPD. Multilevel mixed effects models were simulated in R using population parameters obtained from (16) and a correlation of 0.5 between repeated measures. With a two-sided alpha = 0.05, power to identify a common minimum important difference across the 4 study sites at both post-intervention and 3-month follow-up was estimated as 0.54, 0.74 and 0.87, for sample sizes of 60, 80 and 100, respectively.

III. Synthesis of process, outcomes, and cost consequence analyses addressing research question 8

Realist data synthesis will be undertaken. The findings from individual data sources (including participant outcome measures and data from interviews) will be presented in matrices that bring together key issues from the different analyses, to facilitate drawing overall conclusions about the mechanisms of change within each context, which components and mechanisms are most important, and how these might explain observed outcomes or unanticipated outcomes. A concurrent triangulation mixed methods design will be used (18), whereby both quantitative and qualitative data are considered simultaneously. Initially data from each source will be analysed separately (to reach separate conclusions) and then there will be cross-method analysis to reveal meta-inferences that will inform conclusions related to implementation models, context, mechanisms and outcomes (19).

WP2: "Dissemination for Implementation" workshops addressing research question 6. Two "Dissemination for Implementation" virtual workshops will be held: an interim one at month 12 and a final one at month 23. These online events will consist of information sharing and also an 'action planning' component for participants to reflect on how they can integrate study findings into their ongoing service delivery. These events will also act as 'data gathering' opportunities for the study, hence their inclusion as an objective: gathering data on potential opposition and barriers to implementation, how services make sense

For peer review only - http://bmjopen.bmj.com/site/about/guidelines.xhtml

of and use our study findings and apply these to their own services. We expect the key people who lead and manage community rehabilitation services to attend and to leave with an action plan, and will use a targeted approach to promoting the workshops among the intended audience.

The workshops will be delivered according to a theoretically driven structure detailing: actions; theorised mechanisms of change; and expected outputs (See additional documentation). We have successfully used this theory driven workshop structure following both a Department of Health funded national consensus study (20) and an NIHR funded implementation study relating to implementation of a pelvic organ prolapse intervention (21). That workshop resulted in the study findings being adopted at a national level and the recommendations of the study and learning from the implementation day being implemented locally.

Workshop discussions will be audio-recorded (with permission) and/or notes will be taken by research team members on how participants translate study findings in relation to their local circumstances, any opposition or barriers, what level of local action planning is reached or reasons for non-action planning. These data will be qualitatively analysed using the Framework Approach, with specific attention to understanding levels of opposition and action, the discussion of barriers and whether the study findings influence the development of solutions to these barriers, whether study identified 'mechanisms' of change are adopted into local action plans and service delivery options, and which models of delivery seem more favourable for wider adoption and implementation across NHS Health Boards in Scotland.

6.0 Project Management

The study will be sponsored by RGU. It will be overseen by a Study Steering Committee (SSC) comprising of a Chair (independent academic researcher), one further independent academic researcher, a rehabilitation expert, two consumer representatives and the co-chief investigators (CIs). The SSC will meet quarterly. The CIs, co-applicants, and RFs will meet monthly as a Project Management Group (PMG) to consider the overall progress of the study and deal with any arising problems. Two consumer representatives will join the PMG quarterly. In addition to this, smaller sub-groups of members of the PMG will meet as required to ensure that individual elements of the study are progressing. Finally, KC and ED as Co-CIs will meet weekly with the study RFs about the day to day management of study activity. A full risk management plan will be developed prior to study commencement. This will be reviewed quarterly, during our PMG meeting. The project team will adhere to General Data Protection Regulation (GDPR) standards of data protection and data storage.

7.0 Ethical issues

The study will collect data from patient completed outcome measures, anonymous data on long covid rehabilitation service usage, and qualitative data from staff and people who have received long covid rehabilitation.

For peer review only - http://bmjopen.bmj.com/site/about/guidelines.xhtml

The populations being studied are adults (aged > 18 years) and NHS rehabilitation professionals. Potential participants will be either experiencing symptoms of long-covid, or be delivering a rehabilitation service within the context of a pandemic. Both situational contexts highlight the importance of clear participant information sharing about the nature and expectation of study participation and clear signposting that participation is entirely voluntary and can be withdrawn at any point without reason. Considerable consideration has been given to the data collection procedures to ensure that it is not burdensome on individuals or services. We have worked in close collaboration with the included services and with service users to co-construct research tools and agree recruitment and participation methods that are appropriate and proportional.

One of the key ethical concerns in this study is maintaining patient privacy, data management and data security. All study data will be stored on password protected and encrypted university servers (Sharepoint). Data will be re-identifiable in that participants will be allocated a code and coded data will be processed and analysed. The code sheet containing participants' identifiable details will be stored separately and only accessed where necessary (e.g. sending invites for interviews) by nominated members of the study team.

We considered at length and sought expert advice regarding participants potentially reporting clinically worrying scores on the outcome measures, especially GAD-7, PHQ-9 and PCL-5. As the research team will not access participants' scores immediately, we are not in a position to intervene in a timely manner. Therefore, we have provided advice to participants about seeking help from their GP should they be concerned about their mental health after completing the measures (see participant information sheet), and we have also provided contact details for generic mental health support for all participants (also participant information sheet) as well as long-covid support. Focus group participants will also be provided with contact details for generic mental health support and long-covid support.

Having reviewed each of our study actions, we believe that risk of harm from this study is low. Notwithstanding this fact, the research team have established a study protocol to clarify communication and reporting channels if any element of the study methods proves to have significant unforeseen negative consequences. The protocol involves communicating initially with the relevant study site and forming an action plan to address the issue that has arisen. Where the unintended consequence could be relevant to other study sites, the PIs (KC, ED) will inform the pilot site leads of the situation and any suggested mitigating action that could be taken.

For peer review only - http://bmjopen.bmj.com/site/about/guidelines.xhtml

IRAS ID: 295672

8.0 References

(1) Barker-Davies et al. The Stanford Hall consensus statement for post-COVID-19 rehabilitation. *Br J Sports Med* 2020;54:949–959.

(2) Scottish Government. Framework for Supporting People through Recovery and Rehabilitation; during and after the COVID-19 Pandemic. The Scottish Government, August 2020. Available from: file:///C:/Users/shskc/Downloads/framework-supporting-people-through-recovery-rehabilitation-during-covid-19-pandemic%20(2).pdf [Accessed 2nd November 2020].

(3) Scottish Government. Scotland's Digital Heath & Care Strategy. The Scottish Government, April 2018. Available from: https://www.gov.scot/publications/scotlands-digital-health-care-strategy-enabling-connecting-empowering/ [Accessed 2nd November 2020].

(4) Long Covid Support [online] available from: https://www.longcovid.org/ [Accessed 2nd November 2020].

(5) Greenhalgh T et al. Management of post-acute covid-19 in primary care. *BMJ* 2020;370:m3026.

(6) Tenrforde MW et al. Symptom Duration and Risk Factors for Delayed Return to Usual Health Among Outpatients with COVID -19 in a Multistate Health Care Systems Network – United States, March – June 2020. *Morbidity and Mortality Weekly Report* 2020;69(30):993-998.

(7) Greenhalgh T et al. "Long Covid": evidence, recommendations and priority research questions. Written evidence (COV0050) House of Lords Enquiry 2020.

(8) Maxwell E. Living with Covid 19. A dynamic review of the evidence around ongoing Covid 19 symptoms (often called Long Covid). NIHR Centre for Engagement and Dissemination, September 2020. Available from: https://evidence.nihr.ac.uk/themedreview/living-with-covid19/ [Accessed 2nd November 2020.

(9) Sheehy LM. Considerations for Postacute Rehabilitation for Survivors of COVID-19. *JMIR Public Health Surveill* 2020;6(2): e19462.

(10) Andrenelli E et al. Systematic rapid living review on rehabilitation needs due to COVID-19: update to May 31st 2020. *Eur J Phys Rehabil Med* 2020; 56(4):508-514.

For peer review only - http://bmjopen.bmj.com/site/about/guidelines.xhtml

IRAS ID: 295672

(11) Goodwin V et al (2021). Rehabilitation to enable recovery from COVID-19: a rapid systematic review. *Physiother 2021 (in press)* https://doi.org/10.1016/j.physio.2021.01.007

(12) Guttenbruner C et al. Why rehabilitation must have priority during and after the COVID-19 pandemic: A position statement of the global rehabilitation alliance. *J Rehabil Med* 2020;52: jrm00081.

(13) Pawson R, Tilley N. Realistic evaluation: Sage; 1997.

(14) long-covid survey

(15) Framework analysis

(13) Pawson R, Tilley N. Realistic evaluation: Sage; 1997.

(14) long-covid survey

(15) Framework analysis

(16) Nolan 2015. The EQ-5D-5: health status questionnaire in COPD: Validity responsiveness and minimum important difference.

(17) van Hout B et al. Interim scoring for the EQ-5D-5L: mapping the EQ-5D-5L to EQ-5D-3L value sets. *Value Health*. 2012 Jul-Aug;15(5):708-15.

(18) Creswell JW. Research Design: Qualitative, quantitative and mixed methods approaches. 2009 London: Sage.

(19) Teddlie CB, Tashakkori A. Foundations of Mixed Methods Research: Integrating Quantitative and Qualitative Approaches in the Social and Behavioral Sciences. 2009 London: Sage.

(20) Duncan, E et al. Consensus on items and quantities of clinical equipment required to deal with a mass casualties big bang incident: a national Delphi study. *BMC Emergency Med* 2020; 14(1): 5.

(21) Maxwell, M et al. PROPEL: implementation of an evidence based pelvic floor muscle training intervention for women with pelvic organ prolapse: a realist evaluation and outcomes study protocol. *BMC Health Services Research* 2017; 17(1):1-10.

For peer review only - http://bmjopen.bmj.com/site/about/guidelines.xhtml

IRAS ID: 295672

Appendix 1: Study Plan

**Year1**												
**Month:**	**1**	**2**	**3**	**4**	**5**	**6**	**7**	**8**	**9**	**10**	**11**	**12**
	Site preparation & recruitment		Qualitative Round 1					Qualitative Round 2				
				Qualitative data processing & analysis								
			Quantitative data collection, processing & analysis									
				Steering Committee				Steering Committee				Steering Committee
												Workshop 1
	PMG	PMG	PMG	PMG	PMG	PMG	PMG	PMG	PMG	PMG	PMG	PMG
**Year 2**												
**Month:**	**1**	**2**	**3**	**4**	**5**	**6**	**7**	**8**	**9**	**10**	**11**	**12**
	Qualitative Round 3					Qualitative Round 4						
	Qualitative data processing & analysis											
	Quantitative data collection, processing & analysis							Realist synthesis				Reporting
												Outputs
				Steering Committee				Steering Committee				Steering Committee
											Workshop 2	
	PMG	PMG	PMG	PMG	PMG	PMG	PMG	PMG	PMG	PMG	PMG	PMG

PMG=Project Management Group

For peer review only - http://bmjopen.bmj.com/site/about/guidelines.xhtml

IRAS ID: 295672

Version Control

Date	Version	Changes
23.03.21	1.0	N/A
07.03.22	2.0	1. Added objective 3 & 4 to explore community dwelling adults & GPs perceptions & experiences of long-covid rehabilitation<br>2. Updates participants, recruitment, consent, data collection for these additional objectives<br>3. Updated numbering of remaining objectives

For peer review only

For peer review only - http://bmjopen.bmj.com/site/about/guidelines.xhtml

Consolidated criteria for reporting qualitative studies (COREQ): 32-item checklist

Please indicate in which section each item has been reported in your manuscript. If you do not feel an item applies to your manuscript, please enter N/A.

For further information about the COREQ guidelines, please see Tong *et al.*, 2017: https://doi.org/10.1093/intqhc/mzm042

No.	Item	Description	Section #
**Domain 1: Research team and reflexivity**			
Personal characteristics			
1.	Interviewer/facilitator	Which author/s conducted the interview or focus group?	
2.	Credentials	What were the researcher's credentials? *E.g. PhD, MD*	
3.	Occupation	What was their occupation at the time of the study?	
4.	Gender	Was the researcher male or female?	
5.	Experience and training	What experience or training did the researcher have?	
Relationship with participants			
6.	Relationship established	Was a relationship established prior to study commencement?	
7.	Participant knowledge of the interviewer	What did the participants know about the researcher? *E.g. Personal goals, reasons for doing the research*	
8.	Interviewer characteristics	What characteristics were reported about the interviewer/facilitator? *E.g. Bias, assumptions, reasons and interests in the research topic*	
**Domain 2: Study design**			
Theoretical framework			
9.	Methodological orientation and theory	What methodological orientation was stated to underpin the study? *E.g. grounded theory, discourse analysis, ethnography, phenomenology, content analysis*	
Participant selection			
10.	Sampling	How were participants selected? *E.g. purposive, convenience, consecutive, snowball*	
11.	Method of approach	How were participants approached? *E.g. face-to-face, telephone, mail, email*	
12.	Sample size	How many participants were in the study?	
13.	Non-participation	How many people refused to participate or dropped out? What were the reasons for this?	
Setting			
14.	Setting of data collection	Where was the data collected? *E.g. home, clinic, workplace*	
15.	Presence of non-participants	Was anyone else present besides the participants and researchers?	

16.	Description of sample	What are the important characteristics of the sample? *E.g. demographic data, date*	
Data collection			
17.	Interview guide	Were questions, prompts, guides provided by the authors? Was it pilot tested?	
18.	Repeat interviews	Were repeat interviews carried out? If yes, how many?	
19.	Audio/visual recording	Did the research use audio or visual recording to collect the data?	
20.	Field notes	Were field notes made during and/or after the interview or focus group?	
21.	Duration	What was the duration of the interviews or focus group?	
22.	Data saturation	Was data saturation discussed?	
23.	Transcripts returned	Were transcripts returned to participants for comment and/or correction?	
**Domain 3: analysis and findings**			
Data analysis			
24.	Number of data coders	How many data coders coded the data?	
25.	Description of the coding tree	Did authors provide a description of the coding tree?	
26.	Derivation of themes	Were themes identified in advance or derived from the data?	
27.	Software	What software, if applicable, was used to manage the data?	
28.	Participant checking	Did participants provide feedback on the findings?	
Reporting			
29.	Quotations presented	Were participant quotations presented to illustrate the themes / findings? Was each quotation identified? *E.g. Participant number*	
30.	Data and findings consistent	Was there consistency between the data presented and the findings?	
31.	Clarity of major themes	Were major themes clearly presented in the findings?	
32.	Clarity of minor themes	Is there a description of diverse cases or discussion of minor themes?	

When submitting your manuscript via the online submission form, please upload the completed checklist as a Figure/supplementary file.

If you would like this checklist to be included alongside your article, we ask that you upload the completed checklist to an online repository and include the guideline type, name of the repository, DOI and license in the *Data availability* section of your manuscript.

Developed from: Allison Tong, Peter Sainsbury, Jonathan Craig, Consolidated criteria for reporting qualitative research (COREQ): a 32-item checklist for interviews and focus groups, International Journal for Quality in Health Care, Volume 19, Issue 6, December 2007, Pages 349–357, https://doi.org/10.1093/intqhc/mzm042

For peer review only

LOng COvid Rehabilitation
In Scotland: an Evaluation

IRAS ID: 295672

Study Title: Evaluating long-COVID rehabilitation in Scotland

Topic Guide for People with Long COVID

Aim: To explore community dwelling adults' perception and experiences of long-COVID and of attempting to access physical rehabilitation services

The interviews have a general purpose to obtain feedback from people with experience of long-COVID. The interviews will function in an open way to facilitate discussion of issues that are important to the individuals concerned. Therefore, the topic guide is deliberately broad and more specific areas for questioning will be driven by the interests of those taking part

General introduction

The researcher will introduce the purpose of the interview. The interviewee will provide their demographics (sex, age, employment status).

Interview questions

- Can you tell me a bit about your experience of long-COVID?
  - To put at ease, get some general background information, find out main symptoms/duration/progression

- Can you tell me about your experience of accessing healthcare for support with long-COVID?
  - Explore if/what/when/what happened/what helped or not
  - Explore if offered/accessed/tried to access rehabilitation services (e.g., community rehabilitation/physio/OT/other AHPs) and what the outcome was – if not tried to access why not
  - Explore if any barriers to accessing rehabilitation services
  - Explore if think rehabilitation services could be helpful for them or others with long-covid
  - Explore if they consider themselves to have unmet needs regarding management of long-COVID

- Is there anything else you would like to tell me about your experience of long-COVID or accessing healthcare services that you think would be useful to our study?

LOng COvid Rehabilitation
In Scotland: an Evaluation

IRAS ID: 295672

Topic Guide for GPs

Aim: To explore GPs experiences of managing people with long-covid, in particular people who have symptoms that may benefit from rehabilitation from AHPs (e.g., physiotherapists, occupational therapists, dietitians, speech and language therapists), and their experiences of long-covid rehabilitation services in their area

The interviews have a general purpose to obtain feedback from those involved in managing people with long-covid. The interviews will function in an open way to facilitate discussion of issues that are important to the individuals concerned. Therefore, the topic guide is deliberately broad and more specific areas for questioning will be driven by the interests of those taking part

General introduction

The researcher will introduce the purpose of the interview. The GP will introduce their role, years' experience as GP, and general experience of long-COVID.

Interview questions

- Do you have many patients presenting with long-COVID?
  - Explore numbers/how being "diagnosed"/presenting problems
- How are you managing patients with long-COVID?
  - Explore whether individualised or whether any trends for what patients need
  - Explore self-management/onward referral (where to)
  - Explore if perceived need for rehabilitation (e.g., for reduced exercise tolerance, breathlessness, diet, swallowing etc.)
    - If yes next question
    - If no explore why not?
- What are your perceptions of the value of services such as community rehabilitation for people presenting with long-covid symptoms?
- What community rehabilitation services are available for long-COVID patients in your practice to be referred to?
  - Explore whether aware of CRTs or individual professions (e.g., physio/OT) who may be taking referrals
  - Are there any barriers to referring patients to community rehabilitation?
- Anything else you would like to say about managing patients with long-COVID that might be of use to this study?

RGU ABERDEEN nmahp-ru Nursing, Midwifery and Allied Health Professions Research Unit University of Dundee

For peer review only

BMJ Open

Exploring the perceptions and experiences of community rehabilitation for Long COVID from the perspectives of Scottish General Practitioners and people living with Long COVID: a qualitative study

Journal:	*BMJ Open*
Manuscript ID	bmjopen-2023-082830.R1
Article Type:	Original research
Date Submitted by the Author:	15-Mar-2024
Complete List of Authors:	Cooper, Kay; Robert Gordon University, School of Health Sciences<br>Duncan, Edward; University of Stirling, NMAHP-RU<br>Hart-Winks, Erin; Robert Gordon University, School of Health Sciences<br>Cowie, Julie; Glasgow Caledonian University, NMAHP Research Unit<br>Shim, Joanna; Robert Gordon University, School of Health Sciences<br>Stage, Emma; Robert Gordon University, School of Health Sciences<br>Tooman, Tricia; University of Stirling<br>Alexander, L; Robert Gordon University, School of Health Sciences<br>Love, Alison; Long COVID Scotland<br>Morris, Jacqui H.; University of Dundee, SNHS<br>Ormerod, Jane; Long COVID Scotland<br>Preston, Jenny; NHS Ayrshire and Arran, Occupational Therapy; University of Stirling Department of Nursing and Midwifery, NMAHP Research Unit<br>Swinton, Paul; Robert Gordon University,
**Primary Subject Heading**:	Rehabilitation medicine
Secondary Subject Heading:	General practice / Family practice, Health services research, Qualitative research
Keywords:	Post-Acute COVID-19 Syndrome, REHABILITATION MEDICINE, Primary Health Care, Patient-Centered Care, QUALITATIVE RESEARCH

SCHOLARONE™
Manuscripts

I, the Submitting Author has the right to grant and does grant on behalf of all authors of the Work (as defined in the below author licence), an exclusive licence and/or a non-exclusive licence for contributions from authors who are: i) UK Crown employees; ii) where BMJ has agreed a CC-BY licence shall apply, and/or iii) in accordance with the terms applicable for US Federal Government officers or employees acting as part of their official duties; on a worldwide, perpetual, irrevocable, royalty-free basis to BMJ Publishing Group Ltd ("BMJ") its licensees and where the relevant Journal is co-owned by BMJ to the co-owners of the Journal, to publish the Work in this journal and any other BMJ products and to exploit all rights, as set out in our licence.

The Submitting Author accepts and understands that any supply made under these terms is made by BMJ to the Submitting Author unless you are acting as an employee on behalf of your employer or a postgraduate student of an affiliated institution which is paying any applicable article publishing charge ("APC") for Open Access articles. Where the Submitting Author wishes to make the Work available on an Open Access basis (and intends to pay the relevant APC), the terms of reuse of such Open Access shall be governed by a Creative Commons licence – details of these licences and which Creative Commons licence will apply to this Work are set out in our licence referred to above.

Other than as permitted in any relevant BMJ Author's Self Archiving Policies, I confirm this Work has not been accepted for publication elsewhere, is not being considered for publication elsewhere and does not duplicate material already published. I confirm all authors consent to publication of this Work and authorise the granting of this licence.

For peer review only

TITLE PAGE

TITLE: Exploring the perceptions and experiences of community rehabilitation for Long COVID from the perspectives of Scottish General Practitioners' and people living with Long COVID: a qualitative study.

AUTHORS

Kay Cooper*A, School of Health Sciences, Robert Gordon University, k.cooper@rgu.ac.uk

Edward Duncan*, University of Stirling, edward.duncan@stir.ac.uk

Erin Hart-Winks, School of Health Sciences, Robert Gordon University, e.hart-winks@rgu.ac.uk

Julie Cowie, Glasgow Caledonian University, Julie.Cowie@gcu.ac.uk

Joanna Shim, Robert Gordon University, j.shim1@rgu.ac.uk

Emma Stage, Robert Gordon University, e.stage@rgu.ac.uk

Tricia Tooman, University of Stirling, t.r.tooman@stir.ac.uk

Lyndsay Alexander, Robert Gordon University, l.a.alexander@rgu.ac.uk

Alison Love, Long COVID Scotland, alison.despard@ymail.com

Jacqui Morris, University of Dundee J.Y.Morris@dundee.ac.uk

Jane Ormerod, Long COVID Scotland, longcovidscot@gmail.com

Jenny Preston, Douglas Grant Rehabilitation Centre, Ayrshire Central Hospital, Jenny.Preston@aapct.scot.nhs.uk

Paul Alan Swinton, Robert Gordon University, p.swinton@rgu.ac.uk

*Joint lead authors

ACorresponding author

ABSTRACT

Objectives: To explore the experience of accessing Long COVID community rehabilitation from the perspectives of people with Long COVID and General Practitioners (GPs).

Design: Qualitative descriptive study employing one-to-one semi-structured virtual interviews analysed using the framework method.

Setting: Four NHS Scotland territorial health boards.

Participants: Eleven people with Long COVID (1 male, 10 female; aged 40-65 [mean 53], and 13 GPs (5 male, 8 female).

Results: Four key themes were identified: I) The lived experience of Long COVID, describing the negative impact of Long COVID on participants' health and quality of life; II) The challenges of an emergent and complex chronic condition, including uncertainties related to diagnosis and management; III) Systemic challenges for Long COVID service delivery, including lack of clear pathways for access and referral, siloed services, limited resource and a perceived lack of holistic care, and IV) Perceptions and experiences of Long COVID and its management, including rehabilitation. In this theme, lack of knowledge by GPs and people with Long COVID on the potential role of community rehabilitation for Long COVID was identified. Having prior knowledge of rehabilitation or being a healthcare professional appeared to facilitate access to community rehabilitation. Finally, people with Long COVID wo had received rehabilitation had generally found it beneficial.

Conclusions: There are several patient, GP, and service-level barriers to accessing community rehabilitation for Long COVID. There is a need for greater understanding by the public, GPs, and other potential referrers of the role of community rehabilitation professionals in the management of Long COVID. There is also a need for community rehabilitation services to be well promoted and accessible to the people with Long COVID

For peer review only

for whom they may be appropriate. The findings of this study can be used by those (re) designing community rehabilitation services for people with Long COVID.

Strengths and limitations of this study:

- The issue of accessing Long COVID community rehabilitation was explored from the perspectives of both potential referrers to and recipients of community rehabilitation. One researcher conducted all interviews, ensuring consistency in their conduct.
- Data were analysed and interpreted by multiple researchers, including people with Long COVID.
- The study is limited by the small and predominantly female sample , largely drawn from health boards adopting a similar approach to Long COVID rehabilitation.

Keywords

Post-Acute COVID-19 Syndrome, Rehabilitation medicine, Primary Health Care, Patient-Centered Care, Qualitative Research

Word count: 5,968 (including participant quotes)

Funding statement

This work was supported by the Chief Scientist Office Scotland, grant number COV/LTE/20/29.

For peer review only

MANUSCRIPT

INTRODUCTION

Long COVID (LC) in adults is a multisystemic condition described as signs and symptoms that develop during or after an infection consistent with COVID-19, and continue for more than 12-weeks, which cannot be explained by an alternative diagnosis[1]. While global prevalence remains unclear, the World Health Organisation estimated that 34 million people may have experienced LC by 2022.[2] In the UK, 1.9 million people (2.9% of the population) had self-reported LC as of March 2023.[3]

Rehabilitation for people with LC is recommended in practice guidelines[1, 4] and research[5-7], with UK guidelines recommending personalised and multidisciplinary services.[1] In England, service provision for LC has involved the development of specialised clinics, usually comprising multidisciplinary teams of medical, allied health and psychology professionals.[8] In Scotland, LC rehabilitation is delivered in community (non-hospital) settings by generalist allied health professionals, with local variation in models of service delivery.[9]

People with LC have reported barriers to accessing healthcare and difficulties in navigating disjointed healthcare services.[9-12] GPs (synonymous with Family Physicians/Doctors) are the first point of contact for people with LC and have an important role in ensuring that people receive appropriate treatment[13] and referral to specialist services, including community rehabilitation, when needed.

There is a growing body of research on the lived experience of people with LC[12-15] including barriers to accessing healthcar.e[10-11] Until recently, however,[12] none had focussed on community rehabilitation. There is also a growing body of research on the role of the GP in providing care to people wih LC, including the challenges of diagnosis, medical management, and difficulty accessing community services such as rehabilitation.[16-17]

For peer review only

The current study aimed to add to the growing body of knowledge by exploring people with LC and GPs' perspectives of accessing LC community rehabilitation in the Scottish context, due to the approach to LC in this setting being distinct from other parts of the UK.[9] Rehabilitation in this study is defined as intervention/s aimed at optimising function and reducing disability,[18] delivered by any appropriate healthcare professional in a community setting; typically in clinics (outptaient/ambulatory) and people's homes.

This work was embedded within a larger realist evaluation of LC community rehabilitation in Scotland, which identified low numbers of people with LC receiving community rehabilitation services.[12] We sought approval for an amendment to the study (Ref: 21/WA/0118 A03) to understand potential reasons for these low numbers, in the context of the increasing number of people with LC, recommendations for providing community rehabilitation[1, 4-7] and reports that services existed across Scotland.[9]

This study aimed to address two questions: i) what are the perceptions and experiences of people with LC on accessing rehabilitation for LC? and ii) what are GPs' perceptions and experiences of managing people with LC presenting with symptoms of LC that may be suitable for rehabilitation?

METHODS

Study Design

This was a qualitative descriptive study employing semi-structured interviews with a convenience sample of people with LC and GPs in four of the 14 Scottish territorial health boards. The study followed an a priori protocol (Supplementary File 1) and is reported in keeping with the consolidated criteria for reporting qualitative research (COREQ) [Supplementary File 2].[19]

Participants

For peer review only

The four Scottish health boards were chosen for the realist evaluation study based on variation in population and accessibility (Scottish Government Urban Rural Classification 2020),[20] LC prevalence and rehabilitation service delivery models. Two health boards were offering an integrated LC rehabilitation service (i.e., integrated within existing community rehabilitation pathways), one had recently launched a dedicated LC community rehabilitation service, and one pre-existing dedicated LC service was closed to new referrals due to an increased referral rate combined with reduction in funding and therefore inability to staff the service (personal communication).

A convenience sample of people with LC residing in the four health boards were recruited via social media accounts (Facebook; X [formerly Twitter]) of the research team and their institutions and by Long COVID Scotland, a volunteer-led charity run by people with LC. Inclusion criteria for people with LC were: community-dwelling (i.e., not currently hospitalised); aged 18 or over; experiencing symptoms of LC (with or without a positive COVID-19 test), and experience of accessing or attempting to access healthcare services for possible rehabilitation. Those interested in the study contacted the research team, were sent detailed study information, and provided informed consent (audio recorded) prior to taking part.

A convenience sample of GPs were recruited by email invitation circulated to eligible GP practices by the NHS Research Scotland Primary Care Network coordinator. Inclusion criteria were: GP in a practice within one of the four eligible health boards, and experience of patients with probable LC who may be suitable for rehabilitation. Several recruitment reminders were issued. We aimed to recruit 12-20 people with LC and 8-20 GPs.

Data collection

Interview topic guides [Supplementary File 3] were developed. While not formally pilot tested, they were refined by the research team in consultation with people with lived

For peer review only

experience of LC, and informed by initial findings from our realist evaluation study[12] and the wider literature in the field.

During the period June 2022 to January 2023 semi-structured online interviews (Microsoft Teams) and one telephone interview were conducted by one research assistant [EHW], who received training and supervision from KC and ED. Online interviews have been utilised for some time.[21] Whilst they became a necessity during the COVID-19 pandemic[22] their potential is increasingly recognised.[23] In this study they facilitated participation across a large geographical area and flexibility for participants, whilst avoiding unnecessary social contact for people with LC. No repeat interviews were undertaken. GPs were either in their workplace or homes when interviewed; people with LC were all at home. The female research assistant was a qualified nurse and had worked in critical care from the start of the coronavirus pandemic (March 2020) until December 2021. She had no prior relationship with the GP practices or participants who all understood that she was employed as a research assistant.

Interviews lasted 17 to 47 minutes (mean 23 ±7.8 minutes) and were audio recorded. No other individuals were present during the interviews. No field notes were taken. Interviews with people with LC were transcribed by an external transcription service. Due to the homogeneity of the GP interviews, these were not transcribed but instead were listened to on multiple occasions by two researchers, a method informed by the wider literature.[24-25] Neither transcripts nor audio files were returned to participants for comment or correction.

Data analysis

Data were analysed using the framework method (Gale et al. 2013),[26] which proposes a matrix-based format to facilitate the sharing and management of data as a team, and is widely used in applied health research. Familiarisation with the data began by reading and re-reading the transcripts (People with LC) and listening/re-listening to the

For peer review only

recordings (GPs), making analytical notes that informed the 'working analytical framework' for each participant group.[26] Although line-by-line coding is common in qualitative research, it is also possible to develop a framework without engaging in explicit coding;[27] due to the small scale and nature of the data we adopted the latter approach.

The framework method was used to construct matrices in Microsoft Excel, enabling the data to initially be summarised into broad categories. This was led by EHW, in close consultation with two experienced qualitative researchers (KC, ED). The charted data were then analysed by interpreting within and between participants to identify concepts, which were subsequently grouped into themes, and finally overarching themes consisting of data from both participant groups. This was an iterative process involving multiple researchers (KC, ED, JC, TT, JS, ES) and the wider study team, with subsequent refinement until there was consensus that the data had been comprehensively analysed.

As with our previous study[12] we did not seek participant feedback directly on the findings. However, we presented the findings in a webinar attended by health professionals and people with LC, who endorsed the findings, reporting that the analysis reflected their personal experiences.

Patient and public involvement

Two members of the public with lived experience of LC were core members of the study team (AL, JO). Both contributed throughout the study and were integral to its success, helping with design and identification of important issues to explore. They co-developed study materials and interview topic guides and contributed to analysis and interpretation of findings.

RESULTS

Participant characteristics

For peer review only

Twenty-five people with LC expressed an interest in taking part in the study. Of these 25, one did not meet the inclusion criteria for this study but were invited to take part in the realist evaluation. Thirteen people with LC decided against taking part (reasons unknown). Sixteen GPs expressed an interest, of whom three did not proceed to interview due to logistical challenges. Therefore, eleven people with LC (1 male, 10 female; aged 40-65 [mean 53]) and 13 GPs (5 male, 8 female) provided informed consent and took part in an interview. Figure 1 displays the numbers of participants recruited from each of the four health boards and Table 1 describes participants' demographics. No participants who consented withdrew from the study.

For peer review only

Findings

Framework analysis resulted in four overarching themes: i) The lived experience of Long COVID; ii) The challenges of an emergent and complex chronic condition; iii) Systemic challenges for Long COVID service delivery, and iv) Perceptions and experiences of Long COVID and its management, including rehabilitation. Table 2 identifies the data and participant groups that contributed to each of these themes. Throughout the manuscript participants are referred to by a unique, anonymous identifier (health board A-D, followed by PwLC [person with LC] or GP, followed by participant number).

Table 1 Participant demographics

**GPs**	**n (%)**	**People with Long COVID**	**n (%)**
**Sex**		**Sex**	
**Male**	5 (38)	**Male**	1 (9)
**Female**	8 (62)	**Female**	10 (91)
**Years experience General Practice**		**Employment status**	
		**Employed**	4 (36)
**1-10**	4 (31)	**Long-term sick leave**	4 (36)
**11-20**	4 (31)	**Left employment due to Long COVID**	3 (27)
**>20**	3 (23)		
**NR**	2 (15)		
		**Co-morbidities**	
		**Yes***	5 (45)
		**No**	6 (55)

Key: GP=General Practitioner; NR=Not reported;*co-morbidities included respiratory and thyroid conditions, and ME (Myalgic Encephalomyelitis)

I: The lived experience of Long COVID

People with LC described the negative impact of a range of physical and psychological symptoms on their quality of life and physical and social participation, including employment. Many described the impact as life changing and a source of great frustration. Fatigue was common, as was poor mental health, with symptoms of anxiety and depression described by some participants, with both pharmacological and psychological support being sought.

> *I was desperate, I just had no quality of life at all. I couldn't speak to my friends for coughing, couldn't look after my family because I had no energy, couldn't get out the house, I was housebound for months. It was just rubbish.* [APwLC05]

Employment was a common source of concern. Although some participants had attempted a phased return to work, there was generally a perceived lack of support for returning to work after a lengthy LC-related absence, with a need for vocational rehabilitation and practical support to alleviate financial anxiety identified. Presenteeism was also identified as an issue for some people with LC who were at work but not able to fully function due to their symptoms.

> *Fortunately, I've had it, been off, but then went back to work. I've not stayed off. Maybe that's a problem as well, I've not stayed off long term and listened to my body. I've went back and really threw myself back into my workplace and then suffered in my days off.* [CPwLC01]

II: The challenges of an emergent and complex chronic condition

Many participants had presented to primary care seeking confirmatory tests and onward referral due to their complex symptoms and experienced frustration when GPs were reluctant to diagnose LC. GPs, however, spoke of the challenges associated with providing a diagnosis in the absence of a diagnostic test, and the limited treatment options available to them.

For peer review only

247 **Table 2: Overview of thematic analysis**

Overarching Themes	Themes	Concepts
**I: The lived experience of Long COVID**	**People with LC**<br>Impact of LC on daily life	**People with LC**<br>Psychological impact of LC<br>Physical symptoms of LC<br>Impact of LC on quality of life<br>Impact of LC on ability to work
**II: The challenges of an emergent and complex chronic condition**	**GP**<br>Long COVID as a chronic condition	**GP**<br>Challenges and uncertainty associated with diagnosis<br>Complexities of LC and similarities with chronic conditions (ME/FM/CFS/post-viral)<br>System challenges associated with chronic conditions
	**People with LC**<br>Long COVID as a new and unknown condition	**People with LC**<br>Stigma associated with LC<br>Invisibility of LC<br>Lack of validation<br>Complex symptoms and lack of diagnostic/confirmatory tests<br>Lack of ownership by any given discipline & related difficulty getting referred to secondary care<br>Need for self-advocacy among people with LC
**III: Systemic challenges for Long COVID service delivery**	**GP**<br>Long COVID management in primary care	**GP**<br>Current pathways/lack of pathways<br>Resource issues<br>Training needs<br>Safety netting: need for tests and investigations
	**People with LC**<br>Barriers & facilitators to accessing healthcare support for LC	**People with LC**<br>Lack of referral pathways and access to GP and/or secondary care<br>Lack of available resources with long waiting lists & limited support<br>Need for joined up and person-centred care, especially follow-up on results of tests & investigations<br>Apparent inequities across health boards
**IV: Perceptions and experiences of Long COVID and its management, including rehabilitation**	**GP**<br>Perceptions of LC	**GP**<br>Knowledge & beliefs about LC prevalence<br>Perceptions of symptom presentation<br>Knowledge & beliefs about risk factors

For peer review only

		Knowledge & beliefs about subgroups affected by LC<br>Work-related issues and need for vocational rehabilitation<br>Psychological impact and need for peer support<br>Beliefs and values relating to LC
	**People with LC**<br>Healthcare professionals' knowledge & attitudes	**People with LC**<br>Experiences of support available<br>Importance of validation from primary care<br>Perceptions of HCPs lack of knowledge and understanding of LC<br>Role of self-management in LC<br>Role of pharmacology in LC
	**GP**<br>Long COVID Rehabilitation	**GP**<br>Role of medical staff in LC Rehabilitation<br>Role of allied health professionals in LC Rehabilitation<br>Rehabilitation needs
	**People with LC**<br>Experience of LC services	**People with LC**<br>Experience of support from allied health professionals : education & symptom management<br>Perceptions of usefulness of online resources (mixed views of social media)<br>Views on private healthcare<br>Perceptions and experience of 3rd sector support<br>Perceptions of peer support<br>Perceptions and experience of occupational health support

Key: LC=Long COVID; GP=General Practitioners; ME= Myalgic Encephalomyelitis/Encephalopathy; FM=Fibromyalgia; CFS=chronic fatigue syndrome

For peer review only

I don't know if we will move away from handing out the diagnosis of Long COVID. Because to be honest, I don't often suggest it to the patient as a diagnosis because our options for management are so minimal. So, I tend to, if the patient thinks they've got it, work through that with them. [AGP01]

GPs drew similarities between LC and conditions such as chronic fatigue syndrome/ME and Fibromyalgia, particularly the uncertainties associated with diagnosis and management, conveying the need for a long term conditions service inclusive of LC.

I think we are talking about Long COVID now because everyone is looking at COVID, which is great. But I think it's not the only kind of post-[illness] *treatment type problem that we have nowhere to send people. And I think it's a problem with the health system.* [BGP01]

People with LC also recognised the uncertainties associated with LC. However, many felt they were not heard or believed by healthcare professionals and the wider public and expressed a desire for their symptoms to be validated. Participants reported living with an invisible illness, often attributed to a lack of understanding about how LC affects a person, and a feeling that they were being treated like a malingerer.

There's definitely been a lot more media which is very helpful…there was a middle part where people were quite scathing because in the first part I remember friends saying 'I don't know anybody else that's got Long COVID', and that in itself felt a judgement, but then we hit a middle part where loads of folk were getting COVID but they'd been vaccinated or their bodies just dealt with it differently and they weren't ill, so then there was a huge period of judgement came out, 'well, so-and-so had it and they're fine'. [BPwLC06]

Many People with LC felt their symptoms were not taken seriously enough to gain access to secondary care. Some participants' approach to this was to take control of their own health, learning to advocate for themselves and researching symptoms and self-management interventions online.

For peer review only

Oh, I've turned to Twitter…there is kind of a few people on Twitter that I follow that have been really good and kind of published research papers…I go back to the GP and ask about stuff, so I'm just having to kind of search for it myself…So you're not only having to deal with the illness you have to kind of then navigate kind of like, where am I going to get help from? [DPwLC02]

III: Systemic challenges for Long COVID service delivery

Healthcare system-related challenges included the lack of clear pathways for accessing services, siloed specialised services, limited resources (resulting in long waiting times), and the lack of holistic care, with limited resources identified as one of the main barriers. People with LC described these challenges as contributing to communication breakdowns, a lack of accountability over chronic conditions, and the need for self-advocacy amongst people with LC.

"I feel that if there'd been a more joined up approach, somebody would have been like "wait a minute, she can't be on that for four months on that dose ...because my doctor at the time had a consult thing online. So all I would do is type in what my symptoms still were and the chemist would deliver drugs. I didn't physically talk to anybody. So, that was obviously a huge failing, and I think doctors really realised that should never have happened but they had such shortages and still have such shortages" [BPwLC05]

GPs reported a lack of pathways and processes for managing patients with LC, including identifying and tracking patients along the care trajectory and establishing consistent management approaches. The nature of the emergent condition made it challenging to identify appropriate services to refer patients on to. GPs reported that patients seldom met the criteria for existing rehabilitation programmes (e.g., cardiac/respiratory). They also reported that they often carried out tests and investigations to exclude other differential diagnoses as a means of 'safety-netting', but some were mindful that this could delay rehabilitation and recovery.

For peer review only

There is a risk that we hold up rehabilitative inputs until we fully investigated things and we are entirely assured that there's nothing going on. So, we should perhaps be blending things a little better. [BGP02]

People with LC experienced difficulty accessing both primary and secondary care. GPs were aware of the significant pressure secondary care were facing which often resulted in long waiting times for specialties. As a result, patients often re-presented to primary care for ongoing issues leaving GPs with limited options.

I didn't have the energy to argue with the receptionist at the GP surgery, and that's the honest truth, I just didn't have it in me to phone and try and explain it all again. So, I lost nineteen pounds in four weeks because I just couldn't eat because I felt sick. But even that wasn't worth the battle I would have to get past a GP receptionist. [APwLC05]

We were getting a bit frustrated referring patients to secondary care for help. There wasn't much coming through. They were already dealing with backlog enough and they're getting piled up with these other things happening. I understand their limitations, entirely…I don't think that any I have referred [to respiratory or cardiovascular] *have been seen yet. …* [CGP03]

People with LC reported an awareness of variation in access to LC rehabilitation, and the apparent inequity compared to LC clinics in England. Some participants sought private healthcare and reported subsequent improvements in their condition. People with LC emphasised the need for a more joined-up service to improve communication and coordination of care. They wanted healthcare professionals to focus on the condition as a whole and not just specific symptoms.

I just feel sometimes having that one person, like I know a lot of people have a consultant that they go to and that's the person that they speak to, or the centre that they go to, for support. There's a group of people perhaps that they deal

For peer review only

> *with, but they get it. They're a familiar face and that makes sense. But you feel a bit of a pariah, to be honest, with Long COVID.* [BPwLC06]

> GPs also acknowledged the importance of a more holistic approach to LC care including psychological and physical support. They suggested the need for integrated multidisciplinary management providing support for a complex range of symptoms. *If there was a secondary care service set up to see a certain number of patients per day then presumably they would probably allow further, probably allow longer appointments and would be more of a multi-disciplinary approach so that patients would have a bit more time to kind of unpick everything that's going on because there definitely is, probably a kind of you know, whole sort of bio-psycho-social thing going on here [AGP02]*

IV: Perceptions and experiences of Long COVID and its management, including rehabilitation

GPs knowledge, attitudes, and perceptions of Long COVID

In health boards where LC management was integrated into existing community services with less clear pathways to rehabilitation (health boards A & B), there was a perceived lack of demand by GPs for LC-specific services. GPs reported low numbers (1-6 per week) presenting for support with symptoms of LC.

> *Maybe 10-12* [Long COVID patients have presented] *in total. But I don't know whether they're all coming to us. They might be just suffering in silence.* [CGP03]

Patients were commonly reported to be of working age and were perceived to be fit and healthy prior to coronavirus infection. Some GPs also reported that females were more commonly presenting with symptoms associated with LC than males.

Some GPs attributed the low patient numbers to natural resolution and the ability to self-manage on the basis that patients were not reconsulting or requesting further sick lines.

For peer review only

> *"Most people are pretty sensible and they know, they are educated and I am finding that patients that are coming with long covid symptoms, or being quite certain that they have long covid they have educated themselves about it so, quite often actually they would come and they would sometimes know much more than the doctor about it because they have done their own research about it and they will likely know that there is nothing else"* [BGP05]

However, some GPs and most people with LC believed that people were not presenting to primary care because they believed there was no support available.

> *A lot of patients don't necessarily consult because probably they are seeing things in the media and things, you know aware that there aren't particular treatments. So, they just think it's par for the course that they feel like that.* [AGP02]

> *"I stopped contacting the GP because I just feel I'm wasting their time"* [APwLC03]

People with LC recognised that GPs were under pressure and some attributed their reduced attendance at GP practices to feeling like they were an additional burden on the healthcare system. They spoke of withholding information related to their symptoms due to an awareness that GPs were time pressured, and expressed concerns associated with ensuring appointments were productive.

> *I know there are things that I haven't raised with a GP because I'm aware that they're time pressured, I've raised about ten symptoms already in my consultation with them and I know I've got another three sitting on my list, but I can't bring that into the situation…and I'm potentially sitting on stuff that I should have discussed* [BPwLC06]

People with LC also reported concerns over GPs apparent lack of specialist LC knowledge.

For peer review only

But the last time I went to the GP they said, 'we find people with Long COVID know more about it than we do.' And I thought that doesn't really fill you with great enthusiasm. [DPwLC04]

Most GPs acknowledged that LC has impacted people living with the condition and conveyed a need for education associated with recovery and returning to work. They reported that patients felt pressured to return to work without appropriate support in place to assist workplace integration. The need for psychological support was also expressed by both GPs and People with LC. Peer support was identified as a useful resource for people with LC, where they can be supported by others experiencing similar symptoms.

It's just reassuring to know that you're not alone in this. Misery loves company, and it's good to know that there are other people who have this, because otherwise it would become kind of depressing. And it helps put things in perspective, that you know that as bad as you feel someone else is probably feeling worse. [DPwLC06]

A Minority of GPs did not perceive the need for a specialised LC service. Some GPs referred to a scepticism among their colleagues about LC in general:

It's not only for people, it's also for the medical professionals to believe as well that this is a problem. I think there is still some scepticism among medical professionals as well, still, about this being accepted and treated. [CGP03]

One GP described themselves as being slightly cynical about LC and wary of the need for rehabilitation, suggesting that patients should *"wait it out"* and they will get better, likening LC and its impact to 'flu.

"I think it's exactly like flu. The same applies in flu. You get lots of people that get it. Most people are not terribly well with it, few people get flu without knowing they've had it. Some people recover quickly, some take a longer time to recover and some die" [AGP04]

For peer review only

Perceptions and experiences of rehabilitation and other Long COVID services

A range of perceptions regarding LC rehabilitation was held by both participant groups. Some people with LC lacked knowledge of the potential role of rehabilitation professionals in supporting people with LC. This view was shared by some GPs who reported a limited understanding of the role of rehabilitation in the management of LC.

> *Physio, I can't really see much of a role. But that could be my lack of knowledge about it because with the patients I have spoken to it's not really so much of a physical thing, it's not like a particular joint pain as such that they would benefit from a physio. It's more the kind of cognitive aspect, maybe an OT, but I don't know what they would add.* [BGP05]

Some People with LC had enough knowledge of rehabilitation to request referral or refer themselves to rehabilitation services where this was an option. In most cases these participants were on waiting lists and had been for some time. Although we did not record participants' job titles (for those in employment), several participants in this category disclosed during the interviews that they were healthcare professionals, with prior knowledge of, or colleagues working in, rehabilitation services. A third group of People with LC reported a need for LC rehabilitation services in their health board area, suggesting a lack of services and/or their promotion.

The three participants who had received rehabilitation for LC were generally positive about their experience, reporting benefits from specific professions (e.g., physiotherapy, speech and language therapy) and interventions (e.g., breathing exercises), with information and advice on LC and symptom management, particularly the use of pacing, being highly valued.

> *The biggest help was speaking to Speech and Language... she gave me lots of information that was very interesting, and lots about the biology of what's going on with my* [laryngeal] *spasms.* [DPwLC04]

For peer review only

For peer review only - http://bmjopen.bmj.com/site/about/guidelines.xhtml

> *Just having somebody to help you manage what that should look like, what is too much, because you can read about pacing, you can chat about it online with other people with Long COVID, but trying to get a model that fits for you as an individual is actually really hard without support.* [DPwLC06]

This contrasted with the views of People with LC of generic self-management booklets, which were commonly reported as lacking person-centredness.

> *When I did refer myself to the* [specialist] *team, I got a booklet, a massive booklet through the post, that says this that and the other. But it's such an individual, highly differentiated set of symptoms that any one person can have, just none of it was particularly relevant to me.* [DPwLC01]

One participant had sought informal rehabilitation from a personal trainer but felt that it was not helpful due to its intensity, feeling that the trainer did not have the requisite LC knowledge to support individuals effectively.

> *"Just with having this discussion it's like a wee light bulb moment that I'm having, that I'm thinking I've tried the PT* [personal training]*, it was too intense, threw the towel in."* [DPwLC01]

Despite the lack of knowledge of the potential role of rehabilitation demonstrated by some GPs, most reported that they would engage with a dedicated LC rehabilitation service, as it would provide an onward referral route for patients, particularly as they commonly reported being limited by secondary care referral criteria. Several GPs felt that a multidisciplinary team approach could be beneficial for their patients and could provide the validation that patients needed. Some also believed that earlier pulmonary rehabilitation could contribute to better functional recovery.

> *I think they feel quite isolated actually and I think it would be useful even if objectively…there's not a huge improvement. I think psychologically it would be really important for them. Someone to believe them, to see what's happening, and just thinking someone's looking out for them.* [BGP01]

For peer review only

People with LC reported accessing a range of other LC services and sources of support. For some, occupational health services were helpful in supporting return to work. Support from a charity was reported to be useful for some, whilst others found it too generic for their needs. Some participants found support from peers with similar experience of LC was helpful and provided validation.

Some people with LC accessed positive peer support via social media. However, others reported negative experiences and safety concerns regarding social media. The lack of monitoring in online forums contributed to some people feeling vulnerable to receiving incorrect information and negativity from others.

Many people with LC reported resorting to online information, with some taking this information to their GP consultation. Finally, as reported above, some turned to private healthcare in response to access issues and long waiting times, including psychological therapy, GP, medical specialties, and physiotherapy.

DISCUSSION

We explored the issue of access to community rehabilitation for LC from the perspectives of people with LC and GPs in four Scottish health boards. We identified several systemic challenges for LC service delivery which related to access, siloed services, limited resources, and a perceived lack of holistic care, causing frustration for both GPs and people with LC. Similar challenges have been reported in the international qualitative literature from the perspectives of people with LC[28-30] and healthcare professionals.[31] To our knowledge, this is the first study to focus on community rehabilitation. Although a minority of GPs expressed scepticism about LC and the need for rehabilitation and other services for this patient group, people with LC and most GPs agreed on the need for accessible, person-centred services and support, in keeping with previous research recommending collaborative support mechanisms[14] and improved care coordination for people with LC.[29]

For peer review only

Regarding community rehabilitation specifically, we found that: i) some people with LC and some GPs lacked knowledge on the potential role of community rehabilitation in the management of LC; ii) having prior knowledge of rehabilitation or being a healthcare professional appeared to facilitate access to community rehabilitation, and iii) people with LC who had received rehabilitation generally found it beneficial. Due to the lack of knowledge and difficulty accessing rehabilitation however, people with LC had accessed a range of other services and sources of support, with varying success.

The negative impact of LC on the health and quality of life of our study participants is in keeping with other studies[32] and demonstrates the influence of LC on a sample living in Scotland almost 3-years on from the start of the pandemic. The persisting prevalence and impact of LC on people's lives further emphasises the need for support and services such as community rehabilitation to be available, in keeping with recommendations and research findings.[1,4-7,33-34]

People with LC should have access to personalised and multidisciplinary rehabilitation[1] and such rehabilitation is reportedly available throughout Scotland[9] delivered by a variety of service models. This study highlights some potential reasons for the mismatch between recommendations, reported service availability and low numbers of people with LC accessing community rehabilitation.[12]

Lack of GP knowledge regarding community rehabilitation and its potential role in LC may, in part, be attributed to the nature of LC as a new condition that health professionals are still learning how to manage. Previous research has reported a lack of GP understanding of the role of rehabilitation professionals in the management of conditions commonly encountered in primary care; for example, physiotherapists role in in osteoarthritis management.[35] Internationally, public understanding of the role of occupational therapy has been reported as limited.[36] Therefore, in the context of a new condition with an evolving evidence-base, it is perhaps not surprising that GPs and people with LC may have limited understanding of what rehabilitation professionals can offer to this patient population. The reluctance to promote the availability of services in

For peer review only - http://bmjopen.bmj.com/site/about/guidelines.xhtml

some areas, due to pre-existing resourcing and anticipated demand-capacity issues may also have limited access to LC rehabilitation.[12]

Issues with promotion of services, clarity of pathways and interdisciplinary communication between GPs and rehabilitation professionals have been reported previously.[35, 37] The findings of this study suggest that further improvements in communication and collaborative working[35] may be required to enhance access to community rehabilitation for people with LC. Indeed, the finding that prior knowledge of rehabilitation, and being a healthcare professional facilitated access to community rehabilitation is further evidence that successfully navigating the referral system is challenging.

The findings of people with LC who had managed to access community rehabilitation being satisfied with it, and GPs wanting a LC service to refer patients to are in keeping with previous research.[16] The challenge not only lies in availability of such services, but clearly in people with LC and GPs awareness of the benefits of these services, their active promotion, and clear and timely accessibility of rehabilitation services. There is therefore a need to overcome the systemic challenges to accessing timely rehabilitation reported in this study. Considering the ongoing nature of living with LC, these challenges are likely to continue beyond the time and resource constraints of Government funding provision for existing LC rehabilitation within rehabilitation services that were already historically underfunded and considered 'Cinderella services'.[12]

Lack of capacity in the UKs rehabilitation services is not a new phenomenon,[38] but it has arguably been further highlighted since COVID-19[39] Worldwide, there is a renewed focus on the importance of rehabilitation, and the size of the unmet need.[18] This study, whilst focussed on LC, provides additional evidence of the need for increasing capacity in community rehabilitation services.[39]

Strengths and limitations

For peer review only

This study explored the issue of accessing LC community rehabilitation in the Scottish context and included the perspectives of those referring and potentially being referred. One researcher conducted all interviews to ensure consistency and multiple researchers were involved in analysing and interpreting the data, including people with lived experience of LC. There are, however, some limitations.

The sample was one of convenience which limits generalization, and although we recruited to target for the GP sample, there was under-representation from the two health boards with dedicated LC services. Therefore, the data largely represents the views of GPs from health boards where LC services were integrated into existing community rehabilitation services; it is possible that GPs views of dedicated LC services may be different.

We recruited a mostly female sample of people with LC, and mostly from health boards with integrated or a halted dedicated LC service. Their views on accessing LC services may therefore have been biased. Although LC has been reported as more common in females,[40] the under-representation of males in this study, as with others in the field,[14] is a limitation that needs to be addressed in future research. Our sample size was small, but similar to previous studies exploring LC and its management.[14, 31, 33] Recruitment of people with LC was likely limited by our reliance on social media and one LC charity; however, both mechanisms had the potential to reach many people.

Despite these challenges, both participant groups provided consistent understandings of the challenges they faced when dealing with LC. Therefore, while data saturation is not claimed, it does appear that we achieved adequate data sufficiency to be confident that the findings reflect key issues within each participant group.[41]

We conducted interviews online with one by telephone, which may have affected rapport and depth of interaction, but provided flexibility for participants and the research team. The GP data was not transcribed, which is commonly seen as a routine step in qualitative

For peer review only

studies. Finally, participants did not check the transcripts or findings, but healthcare professionals and people with LC did comment on the findings at an open workshop.

Implications for practice and research

There is a need for greater understanding by the public, GPs, and other potential referrers of the role of community rehabilitation professionals in the management of LC. There is an equally important need for community rehabilitation services to be well promoted and accessible to the people with LC for whom they may be appropriate. Long COVID is still a prevalent condition whose impact on individuals can be profound. The need for community rehabilitation for people with LC is likely to persist. Service providers should therefore consider availability and accessibility of LC rehabilitation and ensure adequate interprofessional communication and collaboration to enhance the experience for people with LC.

CONCLUSION

We have provided further understanding of the barriers to accessing LC community rehabilitation in the Scottish context by exploring the perceptions and experiences of key stakeholders in the referral process. These findings can be used by those (re)designing community rehabilitation services for people with LC and potentially for other long-term conditions. There remains a need for greater public and GP awareness of the role of rehabilitation professionals in LC.

AUTHOR CONTRIBUTIONS

KC, ED, JM, LA, JP, JO, AL, PS contributed to the study’s conception and design. EHW undertook data collection. ED, KC, EHW, TT, JC, JS, ES undertook analyses and drafted the first version of the manuscript. KC led future iterations of the manuscript. All authors read and commented on the manuscript and approved the final version of it. The corresponding author attests that all authors meet authorship criteria and that nobody meeting the criteria have been omitted. ED and KC have joint responsibility for the

For peer review only

research conduct of the study, had access to the data, and controlled the decision to publish.

COMPETING INTERESTS

All authors have completed the ICMJE uniform disclosure form at www.icmje.org/coi_disclosure.pdf and declare: all authors had financial support from the Chief Scientist Office Scotland (grant number COV/LTE/20/29) for the submitted work; ED has received research grants from NIHR and Scottish Government for the following studies: Caring for Long COVID in primary care and DBI COVID study respectively. JM has received funding for LOCOMOTION (Long COVID multidisciplinary consortium: optimising treatments and services across the NHS). No other relationships or activities that could appear to have influenced the submitted work are declared by any of the authors.

FUNDING STATEMENT

This work was supported by the Chief Scientist Office Scotland, grant number COV/LTE/20/29

DATA AVAILABILITY

All data produced in the present study are available upon reasonable request. Robert Gordon University holds the copyright for the full interview transcripts and may grant data sharing permission on request.

ETHICS APPROVAL

Ethical approval was granted from the Wales Research Ethics Committee 6 [21/WA/0118 A03], and each of the four Health Boards granted R&D management approval.

ACKNOWLEDGEMENTS

We are grateful to all our participants who gave of their time to take part in interviews. We would like to thank Amanda Cardy, Northeast coordinator NRS Primary Care Network

For peer review only

617 and local principal investigators Lynne Frew, Lynn Morrison, and Gail Thomson-Patel for support with participant recruitment.

For peer review only

REFERENCES

1. NICE. COVID-19 rapid guideline: managing the long term effects of COVID-19, 2022.

2. World Health Organization. With the international public health emergency ending, WHO/Europe launches its transition plan for COVID-19. 2023 [Available from: https://www.who.int/europe/news/item/12-06-2023-with-the-international-public-health-emergency-ending--who-europe-launches-its-transition-plan-for-covid-19 accessed 5 October 2023.

3. Office for National Statistics (ONS). Prevalence of ongoing symptoms following coronavirus (COVID-19) infection in the UK: 30 March 2023. 2023 [Available from: https://www.ons.gov.uk/peoplepopulationandcommunity/healthandsocialcare/conditionsanddiseases/bulletins/prevalenceofongoingsymptomsfollowingcoronaviruscovid19infectionintheuk/30march2023 accessed 5 October 2023.

4. World Health Organization. Clinical management of COVID-19: living guideline, 13 January 2023. Geneva: World Health Organization, 2023.

5. Heeney A, Connolly SP, Dillon R, et al. Post-COVID care delivery: The experience from an Irish tertiary centre's post-COVID clinic. *PLoS One* 2023;18(8):e0289245. doi: 10.1371/journal.pone.0289245 [published Online First: 20230811]

6. DeMars J, Brown DA, Angelidis I, et al. What is Safe Long COVID Rehabilitation? *J Occup Rehabil* 2023;33(2):227-30. doi: 10.1007/s10926-022-10075-2

7. Decary S, De Groote W, Arienti C, et al. Scoping review of rehabilitation care models for post COVID-19 condition. *Bull World Health Organ* 2022;100(11):676-88. doi: 10.2471/BLT.22.288105 [published Online First: 20221003]

8. Dean E. What happens inside a long covid clinic? *BMJ* 2023;382:p1791. doi: 10.1136/bmj.p1791

9. Duncan E, Cooper K, Cowie J, et al. A national survey of community rehabilitation service provision for people with long Covid in Scotland. *F1000Res* 2020;9:1416. doi: 10.12688/f1000research.27894.2 [published Online First: 20201207]

10. Baz SA, Fang C, Carpentieri JD, et al. 'I don't know what to do or where to go'. Experiences of accessing healthcare support from the perspectives of people living with Long Covid and healthcare professionals: A qualitative study in Bradford, UK. *Health Expect* 2023;26(1):542-54. doi: 10.1111/hex.13687 [published Online First: 20221213]

11. Ladds E, Rushforth A, Wieringa S, et al. Persistent symptoms after Covid-19: qualitative study of 114 "long Covid" patients and draft quality principles for services. *BMC Health Serv Res* 2020;20(1):1144. doi: 10.1186/s12913-020-06001-y [published Online First: 20201220]

12. Duncan E, Alexander L, Cowie J, et al. Investigating Scottish Long COVID community rehabilitation service models from the perspectives of people living with Long COVID and healthcare professionals: a qualitative descriptive study. *BMJ Open* 2023;13(12):e078740. doi: 10.1136/bmjopen-2023-078740

13. Kingstone T, Taylor AK, O'Donnell CA, et al. Finding the 'right' GP: a qualitative study of the experiences of people with long-COVID. *BJGP Open* 2020;4(5) doi: 10.3399/bjgpopen20X101143 [published Online First: 20201215]

For peer review only

14. Thomas C, Faghy MA, Owen R, et al. Lived experience of patients with Long COVID: a qualitative study in the UK. *BMJ Open* 2023;13(4):e068481. doi: 10.1136/bmjopen-2022-068481 [published Online First: 20230426]

15. Skilbeck L, Spanton C, Paton M. Patients' lived experience and reflections on long COVID: an interpretive phenomenological analysis within an integrated adult primary care psychology NHS service. *J Patient Rep Outcomes* 2023;7(1):30. doi: 10.1186/s41687-023-00570-2 [published Online First: 20230320]

16. Brennan A, Broughan J, McCombe G, et al. Enhancing the management of long COVID in general practice: a scoping review. *BJGP Open* 2022;6(3) doi: 10.3399/BJGPO.2021.0178 [published Online First: 20220928]

17. Bachmeier BE, Holzle S, Gasser M, et al. How do German General Practitioners Manage Long-/Post-COVID? A Qualitative Study in Primary Care. *Viruses* 2023;15(4) doi: 10.3390/v15041016 [published Online First: 20230420]

18. World Health Organization. Rehabilitation. 2023 [Available from: https://www.who.int/news-room/fact-sheets/detail/rehabilitation accessed 28 September 2023.

19. Tong A, Sainsbury P, Craig J. Consolidated criteria for reporting qualitative research (COREQ): a 32-item checklist for interviews and focus groups. *Int J Qual Health Care* 2007;19(6):349-57. doi: 10.1093/intqhc/mzm042 [published Online First: 20070914]

20. Scottish Government. Scottish Government Urban Rural Classification 2020. 2022 [Available from: https://www.gov.scot/publications/scottish-government-urban-rural-classification-2020/ accessed 05 October 2023.

21. Krouwel M, Jolly K, Greenfield S. Comparing Skype (video calling) and in-person qualitative interview modes in a study of people with irritable bowel syndrome – an exploratory comparative analysis. *BMC Medical Research Methodology* 2019;19(1):219. doi: 10.1186/s12874-019-0867-9

22. Sah LK, Singh DR, Sah RK. Conducting Qualitative Interviews using Virtual Communication Tools amid COVID-19 Pandemic: A Learning Opportunity for Future Research. *JNMA J Nepal Med Assoc* 2020;58(232):1103-06. doi: 10.31729/jnma.5738 [published Online First: 20201231]

23. Keen S, Lomeli-Rodriguez M, Joffe H. From Challenge to Opportunity: Virtual Qualitative Research During COVID-19 and Beyond. *Int J Qual Methods* 2022;21:16094069221105075. doi: 10.1177/16094069221105075 [published Online First: 20220604]

24. Halcomb EJ, Davidson PM. Is verbatim transcription of interview data always necessary? *Appl Nurs Res* 2006;19(1):38-42. doi: 10.1016/j.apnr.2005.06.001

25. Hill Z, Tawiah-Agyemang C, Kirkwood B, et al. Are verbatim transcripts necessary in applied qualitative research: experiences from two community-based intervention trials in Ghana. *Emerg Themes Epidemiol* 2022;19(1):5. doi: 10.1186/s12982-022-00115-w [published Online First: 20220628]

26. Gale NK, Heath G, Cameron E, et al. Using the framework method for the analysis of qualitative data in multi-disciplinary health research. *BMC Med Res Methodol* 2013;13:117. doi: 10.1186/1471-2288-13-117 [published Online First: 20130918]

27. Goldsmith LJ. Using Framework Analysis in Applied Qualitative Research. 2021

For peer review only

28. Brehon K, Miciak M, Hung P, et al. "None of us are lying": an interpretive description of the search for legitimacy and the journey to access quality health services by individuals living with Long COVID. *BMC Health Serv Res* 2023;23(1):1396. doi: 10.1186/s12913-023-10288-y [published Online First: 20231212]

29. MacEwan SR, Rahurkar S, Tarver WL, et al. Patient Experiences Navigating Care Coordination For Long COVID: A Qualitative Study. *Journal of General Internal Medicine* 2024 doi: 10.1007/s11606-024-08622-z

30. Schmachtenberg T, Königs G, Dragaqina A, et al. “There is no one who helps you with it”: experiences of people with long COVID regarding medical care, therapeutic measures, and barriers in the German healthcare system: results of a qualitative study with four focus groups. *BMC Health Services Research* 2023;23(1):1160. doi: 10.1186/s12913-023-10170-x

31. Horlick S, Krysa JA, Brehon K, et al. Exploring Rehabilitation Provider Experiences of Providing Health Services for People Living with Long COVID in Alberta. *International Journal of Environmental Research and Public Health* 2023; 20(24).

32. Rofail D, Somersan-Karakaya S, Choi JY, et al. Thematic analysis to explore patients' experiences with long COVID-19: a conceptual model of symptoms and impacts on daily lives. *BMJ Open* 2024;14(1):e076992. doi: 10.1136/bmjopen-2023-076992 [published Online First: 20240117]

33. Al-Jabr H, Windle K, Clifton A, et al. Patient experiences of the Long COVID–Optimal Health Programme: a qualitative interview study in community settings. *BJGP Open* 2024:BJGPO.2023.0137. doi: 10.3399/bjgpo.2023.0137

34. Raunkiaer M, Joergensen DS, Rasmussen A, et al. Experiences of improvement of everyday life following a rehabilitation programme for people with long-term cognitive effects of COVID-19: Qualitative study. *J Clin Nurs* 2024;33(1):137-48. doi: 10.1111/jocn.16739 [published Online First: 20230524]

35. Okwera A BscPt M, May S Ma GDPFMP. Views of general practitioners toward physiotherapy management of osteoarthritis-a qualitative study. *Physiother Theory Pract* 2019;35(10):940-46. doi: 10.1080/09593985.2018.1459987 [published Online First: 20180416]

36. Rahja M, Laver K. What does the Australian public know about occupational therapy for older people? A population survey. *Aust Occup Ther J* 2019;66(4):511-18. doi: 10.1111/1440-1630.12578 [published Online First: 20190410]

37. Igwesi-Chidobe CN, Bishop A, Humphreys K, et al. Implementing patient direct access to musculoskeletal physiotherapy in primary care: views of patients, general practitioners, physiotherapists and clinical commissioners in England. *Physiotherapy* 2021;111:31-39. doi: 10.1016/j.physio.2020.07.002 [published Online First: 20200711]

38. NIHR Evidence; Rehabilitation: how can services meet demand?; September 2021; doi: 10.3310/collection_47413 [accessed 29 Feburary 2024.

39. Wade DT. The future of rehabilitation in the United Kingdom National Health Service: Using the COVID-19 crisis to promote change, increasing efficiency and effectiveness. *Clinical Rehabilitation* 2021;35(4):471-80. doi: 10.1177/0269215520971145

For peer review only

40. Sylvester SV, Rusu R, Chan B, et al. Sex differences in sequelae from COVID-19 infection and in long COVID syndrome: a review. *Current Medical Research and Opinion* 2022;38(8):1391-99. doi: 10.1080/03007995.2022.2081454

41. Malterud K, Siersma VD, Guassora AD. Sample Size in Qualitative Interview Studies: Guided by Information Power. *Qual Health Res* 2016;26(13):1753-60. doi: 10.1177/1049732315617444 [published Online First: 20160710]

Figure Legend:

Figure 1: Number of GPs and People with Long COVID recruited from each health board

For peer review only

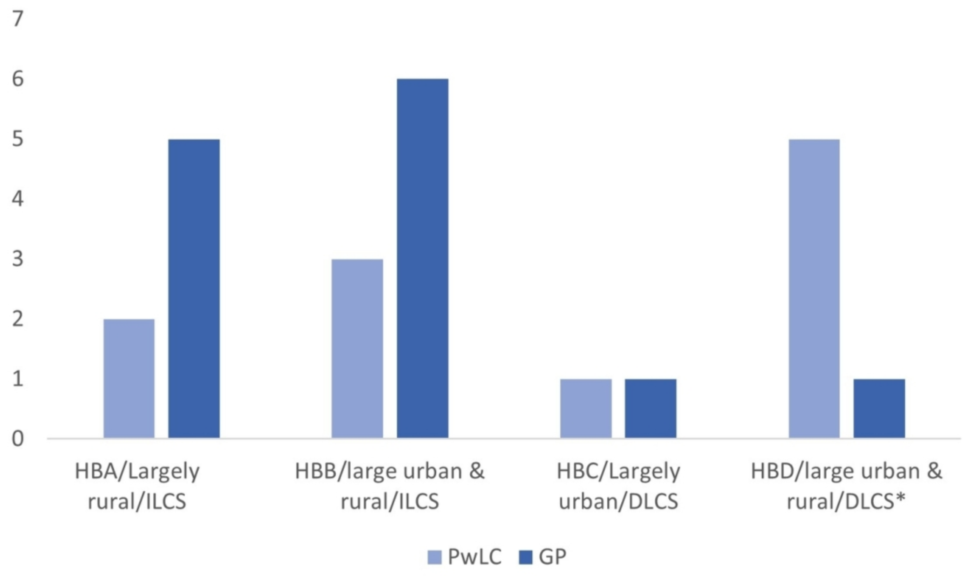

Figure 1: Number of GPs and People with Long COVID recruited from each health board

126x77mm (300 x 300 DPI)

IRAS ID: 295672

Protocol

1.0 Title

Evaluating long-covid rehabilitation in Scotland

2.0 Summary

Studies estimate that 10–35% of people with COVID-19 experience disabling clinical symptoms following the acute phase of their illness (long-covid). Given the prevalence of COVID-19 in Scotland, there is assumed to be a significant number of people experiencing long-covid, and they are starting to be referred to rehabilitation specialists. Community rehabilitation, delivered by physiotherapists, occupational therapists and other health professionals, is a well-established approach to enabling people to maximise their quality of life and recovery. However, community rehabilitation for people with long-covid is in its infancy. There are considerable differences in how community rehabilitation for people with long-covid is currently delivered across Scotland. We want to assess which models of community rehabilitation are most appropriate, in which circumstances. We will evaluate the delivery and outcomes of four different models of community rehabilitation for people with long-covid currently being delivered in Scotland. This will help us to identify which models are more suitable, for which patient groups and in which contexts. We will then hold online workshops with community rehabilitation managers, service leads and others from across Scotland. During the workshops, we will present our findings and support participants to develop evidence-based action plans to improve their local long-covid community rehabilitation services

3.0 Background

3.1 Introduction

This study is an in-depth exploration of models being used to deliver rehabilitation for longer-term effects of COVID-19 in Scotland. Understanding and evaluating how service models are developing, in parallel with evolving knowledge of the impact of COVID-19 on patients, will inform the development of new and responsive patient-centred models that can effectively meet patient and service need. This work is vital. As evidence emerges on the epidemiology and consequences of COVID-19, its impact on individuals and society, and potential burden on health services (1), strategies are required to address the long-term effects of COVID-19. The Scottish Government's Framework for Supporting People through Recovery and Rehabilitation during and after the COVID-19 Pandemic (2) states that "everyone with rehabilitation needs…will be able to access the care and support they need to live well, on their own terms." It also highlights the important role of digital solutions. This research will develop implementable recommendations for post-COVID-19 rehabilitation, congruent with the principles of the Scottish Rehabilitation Framework and Digital Health and Care Strategy (3), and that meet patients' needs.

For peer review only - http://bmjopen.bmj.com/site/about/guidelines.xhtml

3.2: Long-covid

Long-term effects of COVID-19, or "long-covid" has received much recent media attention with action groups calling for more support (4). With no agreed definition, we will use that proposed by Greenhalgh et al (5) of symptoms extending 3-weeks beyond onset (post-acute COVID-19) and 12-weeks beyond onset (chronic COVID-19). Studies on long-term consequences of COVID-19 infection are ongoing; however, it is estimated that 10 – 35% of patients may experience post-acute COVID-19 (5-7). Based on Scottish Government figures for positive COVID-19 tests and survival to date (28th February 2021) this equates to between 19,367 and 67,784 people in Scotland with post-acute COVID-19, with around 2,000 remaining significantly unwell at 12-weeks, commonly due to organ damage (7). Long-covid also affects people with milder symptoms and those who were not hospitalised (6). Rehabilitation therefore needs to be accessible to those presenting in community settings as well as being discharged from hospital.

Reported symptoms vary widely and commonly include respiratory, cardiopulmonary, neurological, musculoskeletal and mental wellbeing sequelae, as well as fatigue and loss of taste and smell (5,6,8). As described above, presentation and severity are variable, and it is important to note that many patients with long-covid will have co-existing conditions that will impact on symptoms and require dual management. Rehabilitation therefore needs to be multidisciplinary, comprehensive, and tailored to patients' needs, in order to maximise function, quality of life and participation in society (9).

3.3: Long-covid rehabilitation

Rehabilitation for long-covid is in its infancy, reflected in a recent living systematic review update finding predominantly expert opinion, indicating that high-quality research is required (10). Research on rehabilitation for other respiratory conditions *may* be generalisable to the COVID-19 population (11), but this is yet to be determined. Several UK expert bodies and published expert opinion recommend a stepped, needs-based rehabilitation approach incorporating information provision, self-management support and specialist services as required. They also recommend that rehabilitation should be individualised and progressive and the use of digital solutions optimised. *How* rehabilitation can be optimally delivered is not known but vital to determine, given that rehabilitation services need to cope with additional COVID-19 demand whilst continuing to provide rehabilitation for other, often vulnerable, patient populations (12). By investigating different ***models of community rehabilitation*** currently implemented in Scotland to evaluate what works, where, with whom and how, we will develop recommendations on *how* rehabilitation can be best delivered across Scotland to meet the needs of patients and services, thereby developing new responsive models of rehabilitation.

4.0 Aims & research questions

The overarching aim of this research is to provide responsive evidence-based

For peer review only - http://bmjopen.bmj.com/site/about/guidelines.xhtml

recommendations to the NHS about how to most effectively deliver community rehabilitation for people with long-covid. We will do this by evaluating the implementation and impact of different emerging models of long-covid rehabilitation in different contexts in Scotland. We will address the following specific research questions:

1. How is rehabilitation for long-covid being delivered in 4 Scottish health boards?
2. What are the barriers and facilitators to implementing long-covid rehabilitation from managerial, rehabilitation staff, and patients' perspectives and experiences?
3. What are the perceptions and experiences of community-dwelling adults with long-covid on accessing rehabilitation for long-covid?
4. What are GPs perceptions and experiences of managing community dwelling adults presenting with symptoms of long-covid that may be suitable for rehabilitation?
5. What are the outcomes of different community rehabilitation models for people experiencing long-covid?
6. What are the costs associated with long-covid rehabilitation in the 4 selected cases?
7. In what ways are the selected long-covid community rehabilitation models successful (or not)?
8. How can we use the knowledge from 1-5 above to inform the development of responsive patient-centred models of rehabilitation that can be adapted to suit the needs of patients and services across Scotland?

5.0 Methodology & methods

We will conduct a mixed-methods case study realist evaluation (RE) of different models of community rehabilitation, in different contextual settings, for people experiencing long-covid in order to understand what works, for whom, in what circumstances and how. The study is informed by the RE framework (13), which is used to study the process and context of 'implementation' and to identify the 'mechanisms of action' that lead to successful (or unsuccessful) delivery. It will also be used to investigate how interventions are being implemented in each case study area. Understanding the local contexts and mechanisms of action will enable us to explain observed outcomes in the different models of community rehabilitation delivery for people with long-covid. Realist Evaluation is a highly appropriate method to use to study the delivery and impact of emerging models of care for long-covid. Realist evaluation emerged in response to the need for knowledge that extends beyond that obtained by controlled trial design (is the intervention effective?) to answering questions of what works for whom and in what circumstances. It is 'realist' in the sense that it does not deny the relevance of robust measurement of outcomes, but also seeks to take account of the context in which interventions are delivered and to identify the mechanisms that make programmes work (or not) to produce the observed outcomes. It is not the interventions themselves that work but the ability and willingness of participants to engage with them.

For peer review only - http://bmjopen.bmj.com/site/about/guidelines.xhtml

IRAS ID: 295672

Setting

Rehabilitation services in four Scottish Health Boards: NHS Ayrshire & Arran, NHS Grampian, NHS Lanarkshire, NHS Tayside. The boards have been selected to represent a diversity of models of rehabilitation in terms of mode of delivery (digital/face-to-face/blended/self-management) and whether they provide a new long-covid service or integrate long-covid rehabilitation into existing services. This information was gained by a Scottish-wide survey of long-covid rehabilitation conducted by the study team (14). The selected NHS boards also represent a range of geographical areas in relation to urban/rural classification, to allow for transferability of findings across Scotland, and boards with sufficient covid-19 cases to make recruitment viable.

The study consists of 2 work packages (WP):

WP1: Case study RE to measure implementation and outcomes of different service models of community rehabilitation for people experiencing long-covid. Within WP1 we will conduct:

I. Process evaluation addressing research questions 1-4

Data will be collected at 4 time-points over a 12-month period across each case to understand local contexts and decisions regarding delivery of community rehabilitation for people with long-covid. Data collection rounds 1 to 4 will describe the long-covid models of community rehabilitation and track local decisions on how services are implementing and developing their long-covid rehabilitation service. Data collected during rounds 1 to 4 will elicit rationales for service delivery decision making and how implementation is supposed to work. Data collection will be obtained from online focus groups (with people who have received long-covid community rehabilitation), and online individual interviews (6-10 per case per round) with managers/service leads/consultants/ and rehabilitation staff with potential to deliver long-covid community rehabilitation. Data from Round 1 will specifically be used to elicit rationales regarding local decisions, identify 'folk theories' surrounding how community rehabilitation delivery is supposed to work, and identify hypothesised mechanisms of (successful) change/implementation. This will establish the locally hypothesised context-mechanism-outcome (CMO) configurations which will then be tested using data gathered in Rounds 2-4.

Participants

1. People who have received long-covid rehabilitation (patients), managers/service leads, and professionals delivering long-covid rehabilitation will be recruited. See table 1 for inclusion/exclusion criteria.
2. Community dwelling adults with long-covid (i.e., not currently receiving community rehabilitation) and GPs in each of the four health boards.

Recruitment

Patients will be sent a study pack in one of the following ways: (i) with their long-covid appointment if it is mailed out to them; in-person from their rehabilitation professional when they receive their first session (either in their home or in a community rehabilitation setting); by mail if their initial session is held virtually. The study pack will consist of: i) letter of invitation, ii) participant information sheet, iii) baseline measures. Patients will therefore be identified by administrative or clinical staff, and will opt-in to the study by completing the

For peer review only - http://bmjopen.bmj.com/site/about/guidelines.xhtml

baseline measures (paper-based or online) and returning them to the research team. Participants will opt-in to taking part in a focus group, and if they wish to do so will provide contact details for the study team to contact them.

Table 1: Inclusion/exclusion criteria participants

	**Inclusion**	**Exclusion**
**Patients**	-Aged 18+<br>-COVID-19 diagnosis (with or without positive covid-19 test) >3-weeks previously<br>-Referred due to presenting with symptoms thought to be due to long-covid (including but not limited to: respiratory, fatigue, musculoskeletal, mental health)<br>-Suitable for rehabilitation (determined by rehabilitation staff)<br>-Able to understand written & spoken English<br>-Willing to take part in study	-Aged <18<br>-Requiring specialist (inpatient) management of specific complications<br>-Unable or unwilling to provide informed consent<br>-Other life-limiting illness with life expectancy <6-months e.g. disseminated malignancy
**Managers/service leads**	Managers/service leads of community rehabilitation services providing long-covid rehabilitation	Managers/service leads of inpatient services
**Rehabilitation professionals**	Healthcare professionals delivering long-covid rehabilitation including but not limited to, physiotherapists, occupational therapists, speech and language therapists, psychologists, medical doctors. Where relevant (i.e. involved in delivering long-covid rehabilitation) healthcare support workers will be included	-Student healthcare professionals<br>-Healthcare professionals providing in-patient rehabilitation
**Community dwelling adults with long-covid**	Aged 18+<br>Self-identify as having experience of long-covid and accessing/attempting to access healthcare services for possible rehabilitation	-Aged <18<br>-Unable or unwilling to provide informed consent<br>-Other life-limiting illness with life expectancy <6-months e.g., disseminated malignancy
**GPs**	GP based in any of the four participating health boards with experience of patients with probable long-covid who	

For peer review only

For peer review only - http://bmjopen.bmj.com/site/about/guidelines.xhtml

IRAS ID: 295672

	may be suitable for rehabilitation	

Rehabilitation professionals and Managers/service leads will be recruited by email with a letter of invitation and participant information sheet attached. The email will come from a relevant gatekeeper e.g. team lead/manager for rehabilitation professionals; sector lead/allied health professions lead for service leads/managers. Participants will opt-in to the study by contacting the research team by email or telephone.

Community dwelling adults with long-covid will be recruited via social media using RGU School of health sciences and NMAHP research unit University of Stirling Facebook and Twitter accounts, via long-COVID Scotland action group, and The Alliance. Following these routes, we anticipate snowball sampling will occur.

GPs will be recruited by email invitation circulated on our behalf by the NRS Primary Care Network**.**

We plan to recruit these two groups of participants for one round of data collection only, anticipating that referral to long-COVID services will pick up in the coming months. However, should this assumption be incorrect, and referrals to services and this study remain low, we will include these two groups in subsequent rounds of data collection also.

Informed consent

Patients returning baseline measures will indicate their consent to taking part in the quantitative data collection by answering a question on the baseline measures paper or electronic form. Participants will self-select to take part in the focus group by leaving their name and contact details at the end of the survey or e-mailing the research team directly. The research fellow (RF) will then contact the participant by telephone or Microsoft Teams to discuss further and if relevant make arrangements for focus group participation. Consent to take part in the focus group will be provided verbally (audio recorded) during a separate telephone or Microsoft Teams call with the RF. A consent form with standard statements will be read to participants and they will have to agree to each statement in order to take part. Rehabilitation professionals and service leads/managers will provide verbal consent prior to commencing their interviews in the same manner.

Community-dwelling adults with long-covid will contact the research team using the details provided in invitation materials. Individuals will then be provided with study information and following discussion with the RF will provide verbal consent to be interviewed as described above.

GPs will likewise contact the research team following invitation emails sent by the NRS Primary Care Research Network, and will likewise provide verbal informed consent.

Data collection

Patients: One online focus group will be conducted in each case study site during each round of data collection (n=4 focus groups of 8-10 participants each). Patients will be purposively sampled from those opting-in to a focus

For peer review only - http://bmjopen.bmj.com/site/about/guidelines.xhtml

group, based on sampling criteria including age, gender, ethnicity, deprivation, and whether they completed rehabilitation or not. In the event that sufficient diversity is not achieved by this method, rehabilitation services will be asked to conduct additional targeted recruitment on our behalf. Focus groups will take place via Microsoft Teams and will each be conducted by 2 trained researchers using a topic guide. Focus groups will last no longer than 2-hours, will include adequate rest breaks, and will be recorded via Teams. To address issues of digital exclusion, a sample of participants who are interested in taking part but unable (or unwilling) to join an online focus group will be invited to provide their views via telephone interview or a short paper-based survey.

Rehabilitation professionals and service leads/managers will take part in a 1-1 interview, conducted via Teams, supported by a topic guide and recorded in the same manner as the focus groups. From those volunteering to take part we will purposively recruit 3-5 professionals and 2 managers per site to be theoretically representative of staff at each site. We anticipate that the same staff will take part in each of the 4 rounds of data collection, unless there are changes in staffing, in which case additional recruitment will be conducted.

Community dwelling adults and GPs will take part in a single 1-1 interview, conducted by Teams, supported by a topic guide and recorded in the same manner as the focus groups. We will aim to recruit 3-5 community dwelling adults and 2-5 GPs at each site.

Data processing

Following data collection, the MP3 recordings will be downloaded by the RF and saved on secure servers at Robert Gordon University (RGU) and Stirling University (UoS) only accessible to the research team. The transcripts will be transcribed by GDPR-compliant transcribers and stored on the same secure servers.

Data analysis

Data will be analysed to articulate the context and mechanisms of action and relate these to observed (quantitative and qualitative) outcomes (CMO configurations). Each data source will be analysed individually in the first instance to reach separate conclusions. Data from meetings and interviews will be analysed using the Framework Approach (15) aided by NVivo (V12) software. For all qualitative datasets, 10% of transcripts will be coded independently by two RFs and the resulting codes discussed, refined and agreed as a final coding frame to be systematically applied to all transcripts. Following familiarisation with the data, a thematic framework will be developed and applied across the data set. Data will then be tabulated and conceptual maps used to make links between themes. Data will be used to construct case summaries at the level of the different modes of delivery and across different sites. All data for each mode of delivery and for each delivery site will be collected together and consistencies/ inconsistencies searched for. The aim of analysis at this stage is to identify the core barriers and facilitators and mechanisms of action within each mode of delivery, the detailed explanations for them and interactions between them.

For peer review only - http://bmjopen.bmj.com/site/about/guidelines.xhtml

IRAS ID: 295672

II. Outcome evaluation and cost-consequence analysis addressing outcomes 5-7

Outcomes data will be collected before and after people receive community rehabilitation in each location/model of service delivery to establish whether improvements are observable. Recruitment of up to 100 people per case study site will be conducted, from month 3 to month 18 of the study, by the staff delivering community rehabilitation in each local site. It is anticipated that each staff member (approximately 4-6 per team) will collect data on between 15-20 people over the 16-month period. The following patient demographic and clinical data will be collected by each team member for each consenting patient:

- Age
- Sex
- Ethnicity
- Occupation
- Social deprivation status in deciles (gathered using home postcode entered into https://simd.scot/#/simd2020/BTTTFTT/14/-2.7960/55.9548/
- Key presenting problems (e.g. musculoskeletal, fatigue, breathlessness, mental health)
- Co-morbidities (e.g. diabetes, hypertension, COPD)
- Smoking status
- Height & Weight (to estimate BMI)
- Duration of rehabilitation (date of first appointment to discharge)
- Number and duration of sessions
- Number and type of professionals seen

Patient outcomes will be collected at baseline immediately prior to first appointment, at the end of the last session of rehabilitation, and at 3-month follow-up. Baseline measures will be provided to patients with their appointment letter or at their first rehabilitation session (in person or by post if session is virtual), and they can complete the paper copy and return to the study team in freepost envelope or they can complete the measures online (Jisc online surveys) as a web address will also be provided. Each participant will have a unique ID allocated by the site administration or clinical staff, which will be documented on the measures. Patients will be instructed to input this unique ID if they complete the measures online. Reminders will be sent by administration/clinical staff to follow-up individuals who do not submit completed measures. Regular communication between the study RFs and case study sites will facilitate reminders and sending discharge ad follow-up measures to participants.

Outcome measures

In keeping with the PHOSP study[1], we include the following outcomes:

[1]http://www.isrctn.com/ISRCTN10980107

For peer review only - http://bmjopen.bmj.com/site/about/guidelines.xhtml

IRAS ID: 295672

Primary Outcome

Quality of Life: EQ-5D-5L utility index and visual analogue scale (VAS).

Secondary Outcomes

Mental Health

- Generalised Anxiety Disorder Assessment (GAD-7)
- Patient Health Questionnaire (PHQ-9)
- Post-Traumatic Stress Disorder Checklist for DSM-5 (PCL-5)

Clinical symptoms

- Dyspnoea12 Questionnaire
- FACIT – Fatigue Questionnaire

Generic

- Nottingham Extended Activities of Daily Living Scale
- General Practice Physical Activity Scale

Covid Specific

Post-COVID-19 Functional Status (PCFS)

Service-related data

In addition to the data collected from consenting patients, rehabilitation staff will collect the demographic and clinical data listed above (age, ethnicity, occupation etc.) for all long-covid referrals during the study period. This will allow us to determine the extent to which our sample is representative of the long-covid population in the four study sites.

Data analysis

Analysis of quantitative data will be address research aims: 3 (What are the outcomes of different community rehabilitation models for people experiencing long-covid?) and 4 (What are the costs associated with long-covid rehabilitation in the 4 selected cases?) Descriptive statistics will be tabulated for all outcomes at each time point, showing means and SDs. To describe representative changes in each outcome measured, generalized linear mixed models will be conducted. Mixed effects models will be used to account for the repeated measures nature of the data and appropriately address missing data without requirement for listwise deletion. Random intercepts and slopes will be included to quantify inter-individual variability in baseline measures and response to rehabilitation as well as general trends. Where suggested by the data and model checking processes, autoregressive models and non-normal link functions will be included to obtain more accurate parameter estimates and their associated standard errors. Addition of fixed effects into models will be included to investigate systematic differences associated with demographic factors (e.g. age, ethnicity, social deprivation status) and study sites where appropriate statistical power exists. Standard model building (e.g. modelling the effects of time [within-person fluctuation to within-person change over time], modelling the effects of predictors [time-invariant predictors to time-varying predictors in models of

For peer review only - http://bmjopen.bmj.com/site/about/guidelines.xhtml

within-person fluctuation and change]) and model checking (e.g. likelihood ratio tests and residual analyses) processes will be used throughout, with all analyses performed using the lme4 package in the statistical environment R.

The primary outcome is general health status measured via the EQ-5D-5L utility index and VAS. The EQ-5D-5L has been selected to differentiate smaller changes in health status and is less prone to ceiling effects (16). Based on NICE recommendations, quality-adjusted life-years will be calculated using tariffs specific to the EQ-5D-5L and will be mapped for reference-case analyses using the function developed by van Hout (17).

The costs associated with long-covid rehabilitation will be quantified through mean values for all outcomes presented for each study site with uncertainty estimated through boot-strapping procedures. Costs incurred at each site from the perspective of the NHS will be presented and will include estimated costs of the differing modes of delivery (digital, self-management and face to face). Unit costs will be obtained and calculated from Public Health Scotland's Cost Book for outpatient allied health professional clinics (R044X), summary of expenditure on community staff and supplies (R550), and community staff WTE and pay (SFR 8.1). ***Statistical Power:*** Estimates of statistical power were based on the primary outcomes and previous research of the minimum important difference for people with COPD. Multilevel mixed effects models were simulated in R using population parameters obtained from (16) and a correlation of 0.5 between repeated measures. With a two-sided alpha = 0.05, power to identify a common minimum important difference across the 4 study sites at both post-intervention and 3-month follow-up was estimated as 0.54, 0.74 and 0.87, for sample sizes of 60, 80 and 100, respectively.

III. Synthesis of process, outcomes, and cost consequence analyses addressing research question 8

Realist data synthesis will be undertaken. The findings from individual data sources (including participant outcome measures and data from interviews) will be presented in matrices that bring together key issues from the different analyses, to facilitate drawing overall conclusions about the mechanisms of change within each context, which components and mechanisms are most important, and how these might explain observed outcomes or unanticipated outcomes. A concurrent triangulation mixed methods design will be used (18), whereby both quantitative and qualitative data are considered simultaneously. Initially data from each source will be analysed separately (to reach separate conclusions) and then there will be cross-method analysis to reveal meta-inferences that will inform conclusions related to implementation models, context, mechanisms and outcomes (19).

WP2: "Dissemination for Implementation" workshops addressing research question 6. Two "Dissemination for Implementation" virtual workshops will be held: an interim one at month 12 and a final one at month 23. These online events will consist of information sharing and also an 'action planning' component for participants to reflect on how they can integrate study findings into their ongoing service delivery. These events will also act as 'data gathering' opportunities for the study, hence their inclusion as an objective: gathering data on potential opposition and barriers to implementation, how services make sense

For peer review only - http://bmjopen.bmj.com/site/about/guidelines.xhtml

of and use our study findings and apply these to their own services. We expect the key people who lead and manage community rehabilitation services to attend and to leave with an action plan, and will use a targeted approach to promoting the workshops among the intended audience.

The workshops will be delivered according to a theoretically driven structure detailing: actions; theorised mechanisms of change; and expected outputs (See additional documentation). We have successfully used this theory driven workshop structure following both a Department of Health funded national consensus study (20) and an NIHR funded implementation study relating to implementation of a pelvic organ prolapse intervention (21). That workshop resulted in the study findings being adopted at a national level and the recommendations of the study and learning from the implementation day being implemented locally.

Workshop discussions will be audio-recorded (with permission) and/or notes will be taken by research team members on how participants translate study findings in relation to their local circumstances, any opposition or barriers, what level of local action planning is reached or reasons for non-action planning. These data will be qualitatively analysed using the Framework Approach, with specific attention to understanding levels of opposition and action, the discussion of barriers and whether the study findings influence the development of solutions to these barriers, whether study identified 'mechanisms' of change are adopted into local action plans and service delivery options, and which models of delivery seem more favourable for wider adoption and implementation across NHS Health Boards in Scotland.

6.0 Project Management

The study will be sponsored by RGU. It will be overseen by a Study Steering Committee (SSC) comprising of a Chair (independent academic researcher), one further independent academic researcher, a rehabilitation expert, two consumer representatives and the co-chief investigators (CIs). The SSC will meet quarterly. The CIs, co-applicants, and RFs will meet monthly as a Project Management Group (PMG) to consider the overall progress of the study and deal with any arising problems. Two consumer representatives will join the PMG quarterly. In addition to this, smaller sub-groups of members of the PMG will meet as required to ensure that individual elements of the study are progressing. Finally, KC and ED as Co-CIs will meet weekly with the study RFs about the day to day management of study activity. A full risk management plan will be developed prior to study commencement. This will be reviewed quarterly, during our PMG meeting. The project team will adhere to General Data Protection Regulation (GDPR) standards of data protection and data storage.

7.0 Ethical issues

The study will collect data from patient completed outcome measures, anonymous data on long covid rehabilitation service usage, and qualitative data from staff and people who have received long covid rehabilitation.

For peer review only - http://bmjopen.bmj.com/site/about/guidelines.xhtml

The populations being studied are adults (aged > 18 years) and NHS rehabilitation professionals. Potential participants will be either experiencing symptoms of long-covid, or be delivering a rehabilitation service within the context of a pandemic. Both situational contexts highlight the importance of clear participant information sharing about the nature and expectation of study participation and clear signposting that participation is entirely voluntary and can be withdrawn at any point without reason. Considerable consideration has been given to the data collection procedures to ensure that it is not burdensome on individuals or services. We have worked in close collaboration with the included services and with service users to co-construct research tools and agree recruitment and participation methods that are appropriate and proportional.

One of the key ethical concerns in this study is maintaining patient privacy, data management and data security. All study data will be stored on password protected and encrypted university servers (Sharepoint). Data will be re-identifiable in that participants will be allocated a code and coded data will be processed and analysed. The code sheet containing participants' identifiable details will be stored separately and only accessed where necessary (e.g. sending invites for interviews) by nominated members of the study team.

We considered at length and sought expert advice regarding participants potentially reporting clinically worrying scores on the outcome measures, especially GAD-7, PHQ-9 and PCL-5. As the research team will not access participants' scores immediately, we are not in a position to intervene in a timely manner. Therefore, we have provided advice to participants about seeking help from their GP should they be concerned about their mental health after completing the measures (see participant information sheet), and we have also provided contact details for generic mental health support for all participants (also participant information sheet) as well as long-covid support. Focus group participants will also be provided with contact details for generic mental health support and long-covid support.

Having reviewed each of our study actions, we believe that risk of harm from this study is low. Notwithstanding this fact, the research team have established a study protocol to clarify communication and reporting channels if any element of the study methods proves to have significant unforeseen negative consequences. The protocol involves communicating initially with the relevant study site and forming an action plan to address the issue that has arisen. Where the unintended consequence could be relevant to other study sites, the PIs (KC, ED) will inform the pilot site leads of the situation and any suggested mitigating action that could be taken.

For peer review only - http://bmjopen.bmj.com/site/about/guidelines.xhtml

IRAS ID: 295672

8.0 References

(1) Barker-Davies et al. The Stanford Hall consensus statement for post-COVID-19 rehabilitation. *Br J Sports Med* 2020;54:949–959.

(2) Scottish Government. Framework for Supporting People through Recovery and Rehabilitation; during and after the COVID-19 Pandemic. The Scottish Government, August 2020. Available from: file:///C:/Users/shskc/Downloads/framework-supporting-people-through-recovery-rehabilitation-during-covid-19-pandemic%20(2).pdf [Accessed 2nd November 2020].

(3) Scottish Government. Scotland's Digital Heath & Care Strategy. The Scottish Government, April 2018. Available from: https://www.gov.scot/publications/scotlands-digital-health-care-strategy-enabling-connecting-empowering/ [Accessed 2nd November 2020].

(4) Long Covid Support [online] available from: https://www.longcovid.org/ [Accessed 2nd November 2020].

(5) Greenhalgh T et al. Management of post-acute covid-19 in primary care. *BMJ* 2020;370:m3026.

(6) Tenrforde MW et al. Symptom Duration and Risk Factors for Delayed Return to Usual Health Among Outpatients with COVID -19 in a Multistate Health Care Systems Network – United States, March – June 2020. *Morbidity and Mortality Weekly Report* 2020;69(30):993-998.

(7) Greenhalgh T et al. "Long Covid": evidence, recommendations and priority research questions. Written evidence (COV0050) House of Lords Enquiry 2020.

(8) Maxwell E. Living with Covid 19. A dynamic review of the evidence around ongoing Covid 19 symptoms (often called Long Covid). NIHR Centre for Engagement and Dissemination, September 2020. Available from: https://evidence.nihr.ac.uk/themedreview/living-with-covid19/ [Accessed 2nd November 2020.

(9) Sheehy LM. Considerations for Postacute Rehabilitation for Survivors of COVID-19. *JMIR Public Health Surveill* 2020;6(2): e19462.

(10) Andrenelli E et al. Systematic rapid living review on rehabilitation needs due to COVID-19: update to May 31st 2020. *Eur J Phys Rehabil Med* 2020; 56(4):508-514.

For peer review only - http://bmjopen.bmj.com/site/about/guidelines.xhtml

IRAS ID: 295672

(11) Goodwin V et al (2021). Rehabilitation to enable recovery from COVID-19: a rapid systematic review. *Physiother 2021 (in press)* https://doi.org/10.1016/j.physio.2021.01.007

(12) Guttenbruner C et al. Why rehabilitation must have priority during and after the COVID-19 pandemic: A position statement of the global rehabilitation alliance. *J Rehabil Med* 2020;52: jrm00081.

(13) Pawson R, Tilley N. Realistic evaluation: Sage; 1997.

(14) long-covid survey

(15) Framework analysis

(13) Pawson R, Tilley N. Realistic evaluation: Sage; 1997.

(14) long-covid survey

(15) Framework analysis

(16) Nolan 2015. The EQ-5D-5: health status questionnaire in COPD: Validity responsiveness and minimum important difference.

(17) van Hout B et al. Interim scoring for the EQ-5D-5L: mapping the EQ-5D-5L to EQ-5D-3L value sets. *Value Health*. 2012 Jul-Aug;15(5):708-15.

(18) Creswell JW. Research Design: Qualitative, quantitative and mixed methods approaches. 2009 London: Sage.

(19) Teddlie CB, Tashakkori A. Foundations of Mixed Methods Research: Integrating Quantitative and Qualitative Approaches in the Social and Behavioral Sciences. 2009 London: Sage.

(20) Duncan, E et al. Consensus on items and quantities of clinical equipment required to deal with a mass casualties big bang incident: a national Delphi study. *BMC Emergency Med* 2020; 14(1): 5.

(21) Maxwell, M et al. PROPEL: implementation of an evidence based pelvic floor muscle training intervention for women with pelvic organ prolapse: a realist evaluation and outcomes study protocol. *BMC Health Services Research* 2017; 17(1):1-10.

For peer review only - http://bmjopen.bmj.com/site/about/guidelines.xhtml

IRAS ID: 295672

Appendix 1: Study Plan

**Year1**												
**Month:**	**1**	**2**	**3**	**4**	**5**	**6**	**7**	**8**	**9**	**10**	**11**	**12**
	Site preparation & recruitment		Qualitative Round 1					Qualitative Round 2				
				Qualitative data processing & analysis								
			Quantitative data collection, processing & analysis									
				Steering Committee				Steering Committee				Steering Committee
												Workshop 1
	PMG	PMG	PMG	PMG	PMG	PMG	PMG	PMG	PMG	PMG	PMG	PMG
**Year 2**												
**Month:**	**1**	**2**	**3**	**4**	**5**	**6**	**7**	**8**	**9**	**10**	**11**	**12**
	Qualitative Round 3					Qualitative Round 4						
	Qualitative data processing & analysis											
	Quantitative data collection, processing & analysis							Realist synthesis				Reporting
												Outputs
				Steering Committee				Steering Committee				Steering Committee
											Workshop 2	
	PMG	PMG	PMG	PMG	PMG	PMG	PMG	PMG	PMG	PMG	PMG	PMG

PMG=Project Management Group

For peer review only

For peer review only - http://bmjopen.bmj.com/site/about/guidelines.xhtml

IRAS ID: 295672

Version Control

Date	Version	Changes
23.03.21	1.0	N/A
07.03.22	2.0	1. Added objective 3 & 4 to explore community dwelling adults & GPs perceptions & experiences of long-covid rehabilitation<br>2. Updates participants, recruitment, consent, data collection for these additional objectives<br>3. Updated numbering of remaining objectives

For peer review only

For peer review only - http://bmjopen.bmj.com/site/about/guidelines.xhtml

Consolidated criteria for reporting qualitative studies (COREQ): 32-item checklist

Please indicate in which section each item has been reported in your manuscript. If you do not feel an item applies to your manuscript, please enter N/A.

For further information about the COREQ guidelines, please see Tong *et al.*, 2017: https://doi.org/10.1093/intqhc/mzm042

No.	Item	Description	Section #
**Domain 1: Research team and reflexivity**			
Personal characteristics			
1.	Interviewer/facilitator	Which author/s conducted the interview or focus group?	
2.	Credentials	What were the researcher's credentials? *E.g. PhD, MD*	
3.	Occupation	What was their occupation at the time of the study?	
4.	Gender	Was the researcher male or female?	
5.	Experience and training	What experience or training did the researcher have?	
Relationship with participants			
6.	Relationship established	Was a relationship established prior to study commencement?	
7.	Participant knowledge of the interviewer	What did the participants know about the researcher? *E.g. Personal goals, reasons for doing the research*	
8.	Interviewer characteristics	What characteristics were reported about the interviewer/facilitator? *E.g. Bias, assumptions, reasons and interests in the research topic*	
**Domain 2: Study design**			
Theoretical framework			
9.	Methodological orientation and theory	What methodological orientation was stated to underpin the study? *E.g. grounded theory, discourse analysis, ethnography, phenomenology, content analysis*	
Participant selection			
10.	Sampling	How were participants selected? *E.g. purposive, convenience, consecutive, snowball*	
11.	Method of approach	How were participants approached? *E.g. face-to-face, telephone, mail, email*	
12.	Sample size	How many participants were in the study?	
13.	Non-participation	How many people refused to participate or dropped out? What were the reasons for this?	
Setting			
14.	Setting of data collection	Where was the data collected? *E.g. home, clinic, workplace*	
15.	Presence of non-participants	Was anyone else present besides the participants and researchers?	

For peer review only

16.	Description of sample	What are the important characteristics of the sample? *E.g. demographic data, date*	
Data collection			
17.	Interview guide	Were questions, prompts, guides provided by the authors? Was it pilot tested?	
18.	Repeat interviews	Were repeat interviews carried out? If yes, how many?	
19.	Audio/visual recording	Did the research use audio or visual recording to collect the data?	
20.	Field notes	Were field notes made during and/or after the interview or focus group?	
21.	Duration	What was the duration of the interviews or focus group?	
22.	Data saturation	Was data saturation discussed?	
23.	Transcripts returned	Were transcripts returned to participants for comment and/or correction?	
**Domain 3: analysis and findings**			
Data analysis			
24.	Number of data coders	How many data coders coded the data?	
25.	Description of the coding tree	Did authors provide a description of the coding tree?	
26.	Derivation of themes	Were themes identified in advance or derived from the data?	
27.	Software	What software, if applicable, was used to manage the data?	
28.	Participant checking	Did participants provide feedback on the findings?	
Reporting			
29.	Quotations presented	Were participant quotations presented to illustrate the themes / findings? Was each quotation identified? *E.g. Participant number*	
30.	Data and findings consistent	Was there consistency between the data presented and the findings?	
31.	Clarity of major themes	Were major themes clearly presented in the findings?	
32.	Clarity of minor themes	Is there a description of diverse cases or discussion of minor themes?	

When submitting your manuscript via the online submission form, please upload the completed checklist as a Figure/supplementary file.

If you would like this checklist to be included alongside your article, we ask that you upload the completed checklist to an online repository and include the guideline type, name of the repository, DOI and license in the *Data availability* section of your manuscript.

Developed from: Allison Tong, Peter Sainsbury, Jonathan Craig, Consolidated criteria for reporting qualitative research (COREQ): a 32-item checklist for interviews and focus groups, International Journal for Quality in Health Care, Volume 19, Issue 6, December 2007, Pages 349–357, https://doi.org/10.1093/intqhc/mzm042

LOng COvid Rehabilitation
In Scotland: an Evaluation

IRAS ID: 295672

Study Title: Evaluating long-COVID rehabilitation in Scotland

Topic Guide for People with Long COVID

Aim: To explore community dwelling adults' perception and experiences of long-COVID and of attempting to access physical rehabilitation services

The interviews have a general purpose to obtain feedback from people with experience of long-COVID. The interviews will function in an open way to facilitate discussion of issues that are important to the individuals concerned. Therefore, the topic guide is deliberately broad and more specific areas for questioning will be driven by the interests of those taking part

General introduction

The researcher will introduce the purpose of the interview. The interviewee will provide their demographics (sex, age, employment status).

Interview questions

- Can you tell me a bit about your experience of long-COVID?
  - To put at ease, get some general background information, find out main symptoms/duration/progression

- Can you tell me about your experience of accessing healthcare for support with long-COVID?
  - Explore if/what/when/what happened/what helped or not
  - Explore if offered/accessed/tried to access rehabilitation services (e.g., community rehabilitation/physio/OT/other AHPs) and what the outcome was – if not tried to access why not
  - Explore if any barriers to accessing rehabilitation services
  - Explore if think rehabilitation services could be helpful for them or others with long-covid
  - Explore if they consider themselves to have unmet needs regarding management of long-COVID

- Is there anything else you would like to tell me about your experience of long-COVID or accessing healthcare services that you think would be useful to our study?

RGU ABERDEEN nmahp-ru Nursing, Midwifery and Allied Health Professions Research Unit University of Dundee

For peer review only - http://bmjopen.bmj.com/site/about/guidelines.xhtml

LOng COvid Rehabilitation
In Scotland: an Evaluation

IRAS ID: 295672

Topic Guide for GPs

Aim: To explore GPs experiences of managing people with long-covid, in particular people who have symptoms that may benefit from rehabilitation from AHPs (e.g., physiotherapists, occupational therapists, dietitians, speech and language therapists), and their experiences of long-covid rehabilitation services in their area

The interviews have a general purpose to obtain feedback from those involved in managing people with long-covid. The interviews will function in an open way to facilitate discussion of issues that are important to the individuals concerned. Therefore, the topic guide is deliberately broad and more specific areas for questioning will be driven by the interests of those taking part

General introduction

The researcher will introduce the purpose of the interview. The GP will introduce their role, years' experience as GP, and general experience of long-COVID.

Interview questions

- Do you have many patients presenting with long-COVID?
    - Explore numbers/how being "diagnosed"/presenting problems
- How are you managing patients with long-COVID?
    - Explore whether individualised or whether any trends for what patients need
    - Explore self-management/onward referral (where to)
    - Explore if perceived need for rehabilitation (e.g., for reduced exercise tolerance, breathlessness, diet, swallowing etc.)
        - If yes next question
        - If no explore why not?
- What are your perceptions of the value of services such as community rehabilitation for people presenting with long-covid symptoms?
- What community rehabilitation services are available for long-COVID patients in your practice to be referred to?
    - Explore whether aware of CRTs or individual professions (e.g., physio/OT) who may be taking referrals
    - Are there any barriers to referring patients to community rehabilitation?
- Anything else you would like to say about managing patients with long-COVID that might be of use to this study?

RGU ABERDEEN nmahp-ru Nursing, Midwifery and Allied Health Professions Research Unit University of Dundee

For peer review only

BMJ Open

Exploring the perceptions and experiences of community rehabilitation for Long COVID from the perspectives of Scottish General Practitioners and people living with Long COVID: a qualitative study

Journal:	*BMJ Open*
Manuscript ID	bmjopen-2023-082830.R2
Article Type:	Original research
Date Submitted by the Author:	11-Apr-2024
Complete List of Authors:	Cooper, Kay; Robert Gordon University, School of Health Sciences<br>Duncan, Edward; University of Stirling, NMAHP-RU<br>Hart-Winks, Erin; Robert Gordon University, School of Health Sciences<br>Cowie, Julie; Glasgow Caledonian University, NMAHP Research Unit<br>Shim, Joanna; Robert Gordon University, School of Health Sciences<br>Stage, Emma; Robert Gordon University, School of Health Sciences<br>Tooman, Tricia; University of Stirling<br>Alexander, L; Robert Gordon University, School of Health Sciences<br>Love, Alison; Long COVID Scotland<br>Morris, Jacqui H.; University of Dundee, SNHS<br>Ormerod, Jane; Long COVID Scotland<br>Preston, Jenny; NHS Ayrshire and Arran, Occupational Therapy; University of Stirling Department of Nursing and Midwifery, NMAHP Research Unit<br>Swinton, Paul; Robert Gordon University,
**Primary Subject Heading**:	Rehabilitation medicine
Secondary Subject Heading:	General practice / Family practice, Health services research, Qualitative research
Keywords:	Post-Acute COVID-19 Syndrome, REHABILITATION MEDICINE, Primary Health Care, Patient-Centered Care, QUALITATIVE RESEARCH

SCHOLARONE™ Manuscripts

I, the Submitting Author has the right to grant and does grant on behalf of all authors of the Work (as defined in the below author licence), an exclusive licence and/or a non-exclusive licence for contributions from authors who are: i) UK Crown employees; ii) where BMJ has agreed a CC-BY licence shall apply, and/or iii) in accordance with the terms applicable for US Federal Government officers or employees acting as part of their official duties; on a worldwide, perpetual, irrevocable, royalty-free basis to BMJ Publishing Group Ltd ("BMJ") its licensees and where the relevant Journal is co-owned by BMJ to the co-owners of the Journal, to publish the Work in this journal and any other BMJ products and to exploit all rights, as set out in our licence.

The Submitting Author accepts and understands that any supply made under these terms is made by BMJ to the Submitting Author unless you are acting as an employee on behalf of your employer or a postgraduate student of an affiliated institution which is paying any applicable article publishing charge ("APC") for Open Access articles. Where the Submitting Author wishes to make the Work available on an Open Access basis (and intends to pay the relevant APC), the terms of reuse of such Open Access shall be governed by a Creative Commons licence – details of these licences and which Creative Commons licence will apply to this Work are set out in our licence referred to above.

Other than as permitted in any relevant BMJ Author's Self Archiving Policies, I confirm this Work has not been accepted for publication elsewhere, is not being considered for publication elsewhere and does not duplicate material already published. I confirm all authors consent to publication of this Work and authorise the granting of this licence.

For peer review only

TITLE PAGE

TITLE: Exploring the perceptions and experiences of community rehabilitation for Long COVID from the perspectives of Scottish General Practitioners' and people living with Long COVID: a qualitative study.

AUTHORS

Kay Cooper*A, School of Health Sciences, Robert Gordon University, k.cooper@rgu.ac.uk

Edward Duncan*, University of Stirling, edward.duncan@stir.ac.uk

Erin Hart-Winks, School of Health Sciences, Robert Gordon University, e.hart-winks@rgu.ac.uk

Julie Cowie, Glasgow Caledonian University, Julie.Cowie@gcu.ac.uk

Joanna Shim, Robert Gordon University, j.shim1@rgu.ac.uk

Emma Stage, Robert Gordon University, e.stage@rgu.ac.uk

Tricia Tooman, University of Stirling, t.r.tooman@stir.ac.uk

Lyndsay Alexander, Robert Gordon University, l.a.alexander@rgu.ac.uk

Alison Love, Long COVID Scotland, alison.despard@ymail.com

Jacqui Morris, University of Dundee J.Y.Morris@dundee.ac.uk

Jane Ormerod, Long COVID Scotland, longcovidscot@gmail.com

Jenny Preston, Douglas Grant Rehabilitation Centre, Ayrshire Central Hospital, Jenny.Preston@aapct.scot.nhs.uk

Paul Alan Swinton, Robert Gordon University, p.swinton@rgu.ac.uk

*Joint lead authors

[A]Corresponding author

ABSTRACT

Objectives: To explore the experience of accessing Long COVID community rehabilitation from the perspectives of people with Long COVID and General Practitioners (GPs).

Design: Qualitative descriptive study employing one-to-one semi-structured virtual interviews analysed using the framework method.

Setting: Four NHS Scotland territorial health boards.

Participants: Eleven people with Long COVID (1 male, 10 female; aged 40-65 [mean 53], and 13 GPs (5 male, 8 female).

Results: Four key themes were identified: I) The lived experience of Long COVID, describing the negative impact of Long COVID on participants' health and quality of life; II) The challenges of an emergent and complex chronic condition, including uncertainties related to diagnosis and management; III) Systemic challenges for Long COVID service delivery, including lack of clear pathways for access and referral, siloed services, limited resource and a perceived lack of holistic care, and IV) Perceptions and experiences of Long COVID and its management, including rehabilitation. In this theme, lack of knowledge by GPs and people with Long COVID on the potential role of community rehabilitation for Long COVID was identified. Having prior knowledge of rehabilitation or being a healthcare professional appeared to facilitate access to community rehabilitation. Finally, people with Long COVID wo had received rehabilitation had generally found it beneficial.

Conclusions: There are several patient, GP, and service-level barriers to accessing community rehabilitation for Long COVID. There is a need for greater understanding by the public, GPs, and other potential referrers of the role of community rehabilitation professionals in the management of Long COVID. There is also a need for community rehabilitation services to be well promoted and accessible to the people with Long COVID

For peer review only

for whom they may be appropriate. The findings of this study can be used by those (re) designing community rehabilitation services for people with Long COVID.

Strengths and limitations of this study:

- The issue of accessing Long COVID community rehabilitation was explored from the perspectives of both potential referrers to and recipients of community rehabilitation. One researcher conducted all interviews, ensuring consistency in their conduct.
- Data were analysed and interpreted by multiple researchers, including people with Long COVID.
- The study is limited by the small and predominantly female sample , largely drawn from health boards adopting a similar approach to Long COVID rehabilitation.

Keywords

Post-Acute COVID-19 Syndrome, Rehabilitation medicine, Primary Health Care, Patient-Centered Care, Qualitative Research

Word count: 5,968 (including participant quotes)

Funding statement

This work was supported by the Chief Scientist Office Scotland, grant number COV/LTE/20/29.

For peer review only

MANUSCRIPT

INTRODUCTION

Long COVID (LC) in adults is a multisystemic condition described as signs and symptoms that develop during or after an infection consistent with COVID-19, and continue for more than 12-weeks, which cannot be explained by an alternative diagnosis.[1] While global prevalence remains unclear, the World Health Organisation estimated that 34 million people may have experienced LC by 2022.[2] In the UK, 1.9 million people (2.9% of the population) had self-reported LC as of March 2023.[3]

Rehabilitation for people with LC is recommended in practice guidelines[1, 4] and research,[5-7] with UK guidelines recommending personalised and multidisciplinary services.[1] Rehabilitation can be defined as intervention/s aimed at optimising function and reducing disability.[8] By definition, rehabilitation is multidisciplinary and applied to a range of health conditions. Whilst it is increasingly recognised that the signs and symptoms of LC share commonalities with other chronic conditions, including fibromyalgia and Myalgic Encephalomyelitis (ME)/Chronic Fatigue Syndrome (CFS)[9] and that condition-specific rehabilitation services may further fragment already scarce resources,[10] different approaches to LC rehabilitation have emerged. In England, service provision for LC has involved the development of specialised clinics, usually comprising multidisciplinary teams of medical, allied health and psychology professionals.[11] In Scotland, LC rehabilitation is delivered in community (non-hospital) settings by generalist allied health professionals, with local variation in models of service delivery.[12]

People with LC have reported barriers to accessing healthcare and difficulties in navigating disjointed healthcare services.[12-15] GPs (synonymous with Family Physicians/Doctors) are the first point of contact for people with LC and have an important role in ensuring that people receive appropriate treatment[16] and referral to specialist services, including community rehabilitation, when needed.

For peer review only

There is a growing body of research on the lived experience of people with LC[15, 17, 18] including barriers to accessing healthcare[13, 14] Until recently, however,[15] none had focussed on community rehabilitation. There is also a growing body of research on the role of the GP in providing care to people wih LC, including the challenges of diagnosis, medical management, and difficulty accessing community services such as rehabilitation.[19, 20]

The current study aimed to add to the growing body of knowledge by exploring people with LC and GPs' perspectives of accessing LC community rehabilitation in the Scottish context, due to the approach to LC in this setting being distinct from other parts of the UK.[12] [8]In this study, community rehabilitation was defined as rehabilitation[10] delivered by any appropriate healthcare professional in a community setting; typically in clinics (outptaient/ambulatory) and people's homes.

This work was embedded within a larger realist evaluation of LC community rehabilitation in Scotland, which identified low numbers of people with LC receiving community rehabilitation services.[15] We sought approval for an amendment to the study (Ref: 21/WA/0118 A03) to understand potential reasons for these low numbers, in the context of the increasing number of people with LC, recommendations for providing community rehabilitation[1, 5, 7] and reports that services existed across Scotland.[12]

This study aimed to address two questions: i) what are the perceptions and experiences of people with LC on accessing rehabilitation for LC? and ii) what are GPs' perceptions and experiences of managing people with LC presenting with symptoms of LC that may be suitable for rehabilitation?

METHODS

Study Design

This was a qualitative descriptive study employing semi-structured interviews with a convenience sample of people with LC and GPs in four of the 14 Scottish territorial health boards. The study followed an a priori protocol (Supplementary File 1) and is reported in

For peer review only

keeping with the consolidated criteria for reporting qualitative research (COREQ) [Supplementary File 2].[21]

Participants

The four Scottish health boards were chosen for the realist evaluation study based on variation in population and accessibility (Scottish Government Urban Rural Classification 2020),[22] LC prevalence and rehabilitation service delivery models. Two health boards were offering an integrated LC rehabilitation service (i.e., integrated within existing community rehabilitation pathways), one had recently launched a dedicated LC community rehabilitation service, and one pre-existing dedicated LC service was closed to new referrals due to an increased referral rate combined with reduction in funding and therefore inability to staff the service (personal communication).

A convenience sample of people with LC residing in the four health boards were recruited via social media accounts (Facebook; X [formerly Twitter]) of the research team and their institutions and by Long COVID Scotland, a volunteer-led charity run by people with LC. Inclusion criteria for people with LC were: community-dwelling (i.e., not currently hospitalised); aged 18 or over; experiencing symptoms of LC (with or without a positive COVID-19 test), and experience of accessing or attempting to access healthcare services for possible rehabilitation. Those interested in the study contacted the research team, were sent detailed study information, and provided informed consent (audio recorded) prior to taking part.

A convenience sample of GPs were recruited by email invitation circulated to eligible GP practices by the NHS Research Scotland Primary Care Network coordinator. Inclusion criteria were: GP in a practice within one of the four eligible health boards, and experience of patients with probable LC who may be suitable for rehabilitation. Several recruitment reminders were issued. We aimed to recruit 12-20 people with LC and 8-20 GPs.

Data collection

For peer review only

Interview topic guides [Supplementary File 3] were developed. While not formally pilot tested, they were refined by the research team in consultation with people with lived experience of LC, and informed by initial findings from our realist evaluation study[15] and the wider literature in the field.

During the period June 2022 to January 2023 semi-structured online interviews (Microsoft Teams) and one telephone interview were conducted by one research assistant [EHW], who received training and supervision from KC and ED. Online interviews have been utilised for some time.[23] Whilst they became a necessity during the COVID-19 pandemic[24] their potential is increasingly recognised.[25] In this study they facilitated participation across a large geographical area and flexibility for participants, whilst avoiding unnecessary social contact for people with LC. No repeat interviews were undertaken. GPs were either in their workplace or homes when interviewed; people with LC were all at home. The female research assistant was a qualified nurse and had worked in critical care from the start of the coronavirus pandemic (March 2020) until December 2021. She had no prior relationship with the GP practices or participants who all understood that she was employed as a research assistant.

Interviews lasted 17 to 47 minutes (mean 23 ±7.8 minutes) and were audio recorded. No other individuals were present during the interviews. No field notes were taken. Interviews with people with LC were transcribed by an external transcription service. Due to the homogeneity of the GP interviews, these were not transcribed but instead were listened to on multiple occasions by two researchers, a method informed by the wider literature.[26,27] Neither transcripts nor audio files were returned to participants for comment or correction.

Data analysis

Data were analysed using the framework method (Gale et al. 2013),[28] which proposes a matrix-based format to facilitate the sharing and management of data as a team, and

For peer review only

is widely used in applied health research. Familiarisation with the data began by reading and re-reading the transcripts (People with LC) and listening/re-listening to the recordings (GPs), making analytical notes that informed the 'working analytical framework' for each participant group.[28] Although line-by-line coding is common in qualitative research, it is also possible to develop a framework without engaging in explicit coding;[29] due to the small scale and nature of the data we adopted the latter approach.

The framework method was used to construct matrices in Microsoft Excel, enabling the data to initially be summarised into broad categories. This was led by EHW, in close consultation with two experienced qualitative researchers (KC, ED). The charted data were then analysed by interpreting within and between participants to identify concepts, which were subsequently grouped into themes, and finally overarching themes consisting of data from both participant groups. This was an iterative process involving multiple researchers (KC, ED, JC, TT, JS, ES) and the wider study team, with subsequent refinement until there was consensus that the data had been comprehensively analysed.

As with our previous study[12] we did not seek participant feedback directly on the findings. However, we presented the findings in a webinar attended by health professionals and people with LC, who endorsed the findings, reporting that the analysis reflected their personal experiences.

Patient and public involvement

Two members of the public with lived experience of LC were core members of the study team (AL, JO). Both contributed throughout the study and were integral to its success, helping with design and identification of important issues to explore. They co-developed study materials and interview topic guides and contributed to analysis and interpretation of findings.

RESULTS

Participant characteristics

For peer review only

Twenty-five people with LC expressed an interest in taking part in the study. Of these 25, one did not meet the inclusion criteria for this study but were invited to take part in the realist evaluation. Thirteen people with LC decided against taking part (reasons unknown). Sixteen GPs expressed an interest, of whom three did not proceed to interview due to logistical challenges. Therefore, eleven people with LC (1 male, 10 female; aged 40-65 [mean 53]) and 13 GPs (5 male, 8 female) provided informed consent and took part in an interview. Figure 1 displays the numbers of participants recruited from each of the four health boards and Table 1 describes participants' demographics. No participants who consented withdrew from the study.

Findings

Framework analysis resulted in four overarching themes: i) The lived experience of Long COVID; ii) The challenges of an emergent and complex chronic condition; iii) Systemic challenges for Long COVID service delivery, and iv) Perceptions and experiences of Long COVID and its management, including rehabilitation. Table 2 identifies the data and participant groups that contributed to each of these themes. Throughout the manuscript participants are referred to by a unique, anonymous identifier (health board A-D, followed by PwLC [person with LC] or GP, followed by participant number).

Table 1 Participant demographics

**GPs**	**n (%)**	**People with Long COVID**	**n (%)**
**Sex**		**Sex**	
**Male**	5 (38)	**Male**	1 (9)
**Female**	8 (62)	**Female**	10 (91)
**Years experience General Practice**		**Employment status**	
		**Employed**	4 (36)
**1-10**	4 (31)	**Long-term sick leave**	4 (36)
**11-20**	4 (31)	**Left employment due to Long COVID**	3 (27)
**>20**	3 (23)		
**NR**	2 (15)		
		**Co-morbidities**	
		**Yes***	5 (45)
		**No**	6 (55)

Key: GP=General Practitioner; NR=Not reported;*co-morbidities included respiratory and thyroid conditions, and ME (Myalgic Encephalomyelitis)

I: The lived experience of Long COVID

For peer review only

People with LC described the negative impact of a range of physical and psychological symptoms on their quality of life and physical and social participation, including employment. Many described the impact as life changing and a source of great frustration. Fatigue was common, as was poor mental health, with symptoms of anxiety and depression described by some participants, with both pharmacological and psychological support being sought.

> *I was desperate, I just had no quality of life at all. I couldn't speak to my friends for coughing, couldn't look after my family because I had no energy, couldn't get out the house, I was housebound for months. It was just rubbish.* [APwLC05]

Employment was a common source of concern. Although some participants had attempted a phased return to work, there was generally a perceived lack of support for returning to work after a lengthy LC-related absence, with a need for vocational rehabilitation and practical support to alleviate financial anxiety identified. Presenteeism was also identified as an issue for some people with LC who were at work but not able to fully function due to their symptoms.

> *Fortunately, I've had it, been off, but then went back to work. I've not stayed off. Maybe that's a problem as well, I've not stayed off long term and listened to my body. I've went back and really threw myself back into my workplace and then suffered in my days off.* [CPwLC01]

II: The challenges of an emergent and complex chronic condition

Many participants had presented to primary care seeking confirmatory tests and onward referral due to their complex symptoms and experienced frustration when GPs were reluctant to diagnose LC. GPs, however, spoke of the challenges associated with providing a diagnosis in the absence of a diagnostic test, and the limited treatment options available to them.

For peer review only

Table 2: Overview of thematic analysis

Overarching Themes	Themes	Concepts
**I: The lived experience of Long COVID**	**People with LC**<br>Impact of LC on daily life	**People with LC**<br>Psychological impact of LC<br>Physical symptoms of LC<br>Impact of LC on quality of life<br>Impact of LC on ability to work
**II: The challenges of an emergent and complex chronic condition**	**GP**<br>Long COVID as a chronic condition	**GP**<br>Challenges and uncertainty associated with diagnosis<br>Complexities of LC and similarities with chronic conditions (ME/FM/CFS/post-viral)<br>System challenges associated with chronic conditions
	**People with LC**<br>Long COVID as a new and unknown condition	**People with LC**<br>Stigma associated with LC<br>Invisibility of LC<br>Lack of validation<br>Complex symptoms and lack of diagnostic/confirmatory tests<br>Lack of ownership by any given discipline & related difficulty getting referred to secondary care<br>Need for self-advocacy among people with LC
**III: Systemic challenges for Long COVID service delivery**	**GP**<br>Long COVID management in primary care	**GP**<br>Current pathways/lack of pathways<br>Resource issues<br>Training needs<br>Safety netting: need for tests and investigations
	**People with LC**<br>Barriers & facilitators to accessing healthcare support for LC	**People with LC**<br>Lack of referral pathways and access to GP and/or secondary care<br>Lack of available resources with long waiting lists & limited support<br>Need for joined up and person-centred care, especially follow-up on results of tests & investigations<br>Apparent inequities across health boards
**IV: Perceptions and experiences of Long COVID and its management, including rehabilitation**	**GP**<br>Perceptions of LC	**GP**<br>Knowledge & beliefs about LC prevalence<br>Perceptions of symptom presentation<br>Knowledge & beliefs about risk factors

For peer review only

	Knowledge & beliefs about subgroups affected by LC<br>Work-related issues and need for vocational rehabilitation<br>Psychological impact and need for peer support<br>Beliefs and values relating to LC
**People with LC**<br>Healthcare professionals' knowledge & attitudes	**People with LC**<br>Experiences of support available<br>Importance of validation from primary care<br>Perceptions of HCPs lack of knowledge and understanding of LC<br>Role of self-management in LC<br>Role of pharmacology in LC
**GP**<br>Long COVID Rehabilitation	**GP**<br>Role of medical staff in LC Rehabilitation<br>Role of allied health professionals in LC Rehabilitation<br>Rehabilitation needs
**People with LC**<br>Experience of LC services	**People with LC**<br>Experience of support from allied health professionals : education & symptom management<br>Perceptions of usefulness of online resources (mixed views of social media)<br>Views on private healthcare<br>Perceptions and experience of 3rd sector support<br>Perceptions of peer support<br>Perceptions and experience of occupational health support

Key: LC=Long COVID; GP=General Practitioners; ME= Myalgic Encephalomyelitis/Encephalopathy; FM=Fibromyalgia; CFS=chronic fatigue syndrome

For peer review only

For peer review only - http://bmjopen.bmj.com/site/about/guidelines.xhtml

I don't know if we will move away from handing out the diagnosis of Long COVID. Because to be honest, I don't often suggest it to the patient as a diagnosis because our options for management are so minimal. So, I tend to, if the patient thinks they've got it, work through that with them. [AGP01]

GPs drew similarities between LC and conditions such as chronic fatigue syndrome/ME and Fibromyalgia, particularly the uncertainties associated with diagnosis and management, conveying the need for a long term conditions service inclusive of LC.

I think we are talking about Long COVID now because everyone is looking at COVID, which is great. But I think it's not the only kind of post-[illness] *treatment type problem that we have nowhere to send people. And I think it's a problem with the health system.* [BGP01]

People with LC also recognised the uncertainties associated with LC. However, many felt they were not heard or believed by healthcare professionals and the wider public and expressed a desire for their symptoms to be validated. Participants reported living with an invisible illness, often attributed to a lack of understanding about how LC affects a person, and a feeling that they were being treated like a malingerer.

There's definitely been a lot more media which is very helpful…there was a middle part where people were quite scathing because in the first part I remember friends saying 'I don't know anybody else that's got Long COVID', and that in itself felt a judgement, but then we hit a middle part where loads of folk were getting COVID but they'd been vaccinated or their bodies just dealt with it differently and they weren't ill, so then there was a huge period of judgement came out, 'well, so-and-so had it and they're fine'. [BPwLC06]

Many People with LC felt their symptoms were not taken seriously enough to gain access to secondary care. Some participants' approach to this was to take control of their own health, learning to advocate for themselves and researching symptoms and self-management interventions online.

For peer review only

Oh, I've turned to Twitter…there is kind of a few people on Twitter that I follow that have been really good and kind of published research papers…I go back to the GP and ask about stuff, so I'm just having to kind of search for it myself…So you're not only having to deal with the illness you have to kind of then navigate kind of like, where am I going to get help from? [DPwLC02]

III: Systemic challenges for Long COVID service delivery

Healthcare system-related challenges included the lack of clear pathways for accessing services, siloed specialised services, limited resources (resulting in long waiting times), and the lack of holistic care, with limited resources identified as one of the main barriers. People with LC described these challenges as contributing to communication breakdowns, a lack of accountability over chronic conditions, and the need for self-advocacy amongst people with LC.

"I feel that if there'd been a more joined up approach, somebody would have been like "wait a minute, she can't be on that for four months on that dose …because my doctor at the time had a consult thing online. So all I would do is type in what my symptoms still were and the chemist would deliver drugs. I didn't physically talk to anybody. So, that was obviously a huge failing, and I think doctors really realised that should never have happened but they had such shortages and still have such shortages" [BPwLC05]

GPs reported a lack of pathways and processes for managing patients with LC, including identifying and tracking patients along the care trajectory and establishing consistent management approaches. The nature of the emergent condition made it challenging to identify appropriate services to refer patients on to. GPs reported that patients seldom met the criteria for existing rehabilitation programmes (e.g., cardiac/respiratory). They also reported that they often carried out tests and investigations to exclude other differential diagnoses as a means of 'safety-netting', but some were mindful that this could delay rehabilitation and recovery.

For peer review only

> *There is a risk that we hold up rehabilitative inputs until we fully investigated things and we are entirely assured that there's nothing going on. So, we should perhaps be blending things a little better.* [BGP02]

People with LC experienced difficulty accessing both primary and secondary care. GPs were aware of the significant pressure secondary care were facing which often resulted in long waiting times for specialties. As a result, patients often re-presented to primary care for ongoing issues leaving GPs with limited options.

> *I didn't have the energy to argue with the receptionist at the GP surgery, and that's the honest truth, I just didn't have it in me to phone and try and explain it all again. So, I lost nineteen pounds in four weeks because I just couldn't eat because I felt sick. But even that wasn't worth the battle I would have to get past a GP receptionist.* [APwLC05]

> *We were getting a bit frustrated referring patients to secondary care for help. There wasn't much coming through. They were already dealing with backlog enough and they're getting piled up with these other things happening. I understand their limitations, entirely…I don't think that any I have referred* [to respiratory or cardiovascular] *have been seen yet. …* [CGP03]

People with LC reported an awareness of variation in access to LC rehabilitation, and the apparent inequity compared to LC clinics in England. Some participants sought private healthcare and reported subsequent improvements in their condition. People with LC emphasised the need for a more joined-up service to improve communication and coordination of care. They wanted healthcare professionals to focus on the condition as a whole and not just specific symptoms.

> *I just feel sometimes having that one person, like I know a lot of people have a consultant that they go to and that's the person that they speak to, or the centre that they go to, for support. There's a group of people perhaps that they deal*

For peer review only

336 *with, but they get it. They're a familiar face and that makes sense. But you feel a*
337 *bit of a pariah, to be honest, with Long COVID.* [BPwLC06]

338 GPs also acknowledged the importance of a more holistic approach to LC care including
339 psychological and physical support. They suggested the need for integrated
340 multidisciplinary management providing support for a complex range of symptoms.

341 *If there was a secondary care service set up to see a certain number of patients*
342 *per day then presumably they would probably allow further, probably allow longer*
343 *appointments and would be more of a multi-disciplinary approach so that patients*
344 *would have a bit more time to kind of unpick everything that's going on because*
345 *there definitely is, probably a kind of you know, whole sort of bio-psycho-social*
346 *thing going on here [AGP02]*

347 IV: Perceptions and experiences of Long COVID and its management, including
348 rehabilitation

349 *GPs knowledge, attitudes, and perceptions of Long COVID*

350 In health boards where LC management was integrated into existing community services
351 with less clear pathways to rehabilitation (health boards A & B), there was a perceived
352 lack of demand by GPs for LC-specific services. GPs reported low numbers (1-6 per
353 week) presenting for support with symptoms of LC.

354 *Maybe 10-12* [Long COVID patients have presented] *in total. But I don't know*
355 *whether they're all coming to us. They might be just suffering in silence.* [CGP03]

356 Patients were commonly reported to be of working age and were perceived to be fit and
357 healthy prior to coronavirus infection. Some GPs also reported that females were more
358 commonly presenting with symptoms associated with LC than males.

359 Some GPs attributed the low patient numbers to natural resolution and the ability to self-
360 manage on the basis that patients were not reconsulting or requesting further sick lines.

For peer review only

"Most people are pretty sensible and they know, they are educated and I am finding that patients that are coming with long covid symptoms, or being quite certain that they have long covid they have educated themselves about it so, quite often actually they would come and they would sometimes know much more than the doctor about it because they have done their own research about it and they will likely know that there is nothing else" [BGP05]

However, some GPs and most people with LC believed that people were not presenting to primary care because they believed there was no support available.

A lot of patients don't necessarily consult because probably they are seeing things in the media and things, you know aware that there aren't particular treatments. So, they just think it's par for the course that they feel like that. [AGP02]

"I stopped contacting the GP because I just feel I'm wasting their time" [APwLC03]

People with LC recognised that GPs were under pressure and some attributed their reduced attendance at GP practices to feeling like they were an additional burden on the healthcare system. They spoke of withholding information related to their symptoms due to an awareness that GPs were time pressured, and expressed concerns associated with ensuring appointments were productive.

I know there are things that I haven't raised with a GP because I'm aware that they're time pressured, I've raised about ten symptoms already in my consultation with them and I know I've got another three sitting on my list, but I can't bring that into the situation…and I'm potentially sitting on stuff that I should have discussed [BPwLC06]

People with LC also reported concerns over GPs apparent lack of specialist LC knowledge.

For peer review only

But the last time I went to the GP they said, 'we find people with Long COVID know more about it than we do.' And I thought that doesn't really fill you with great enthusiasm. [DPwLC04]

Most GPs acknowledged that LC has impacted people living with the condition and conveyed a need for education associated with recovery and returning to work. They reported that patients felt pressured to return to work without appropriate support in place to assist workplace integration. The need for psychological support was also expressed by both GPs and People with LC. Peer support was identified as a useful resource for people with LC, where they can be supported by others experiencing similar symptoms.

It's just reassuring to know that you're not alone in this. Misery loves company, and it's good to know that there are other people who have this, because otherwise it would become kind of depressing. And it helps put things in perspective, that you know that as bad as you feel someone else is probably feeling worse. [DPwLC06]

A minority of GPs did not perceive the need for a specialised LC service. Some GPs referred to a scepticism among their colleagues about LC in general:

It's not only for people, it's also for the medical professionals to believe as well that this is a problem. I think there is still some scepticism among medical professionals as well, still, about this being accepted and treated. [CGP03]

One GP described themselves as being slightly cynical about LC and wary of the need for rehabilitation, suggesting that patients should *"wait it out"* and they will get better, likening LC and its impact to 'flu.

"I think it's exactly like flu. The same applies in flu. You get lots of people that get it. Most people are not terribly well with it, few people get flu without knowing they've had it. Some people recover quickly, some take a longer time to recover and some die" [AGP04]

For peer review only

Perceptions and experiences of rehabilitation and other Long COVID services

A range of perceptions regarding LC rehabilitation was held by both participant groups. Some people with LC lacked knowledge of the potential role of rehabilitation professionals in supporting people with LC. This view was shared by some GPs who reported a limited understanding of the role of rehabilitation in the management of LC.

> *Physio, I can't really see much of a role. But that could be my lack of knowledge about it because with the patients I have spoken to it's not really so much of a physical thing, it's not like a particular joint pain as such that they would benefit from a physio. It's more the kind of cognitive aspect, maybe an OT, but I don't know what they would add.* [BGP05]

Some People with LC had enough knowledge of rehabilitation to request referral or refer themselves to rehabilitation services where this was an option. In most cases these participants were on waiting lists and had been for some time. Although we did not record participants' job titles (for those in employment), several participants in this category disclosed during the interviews that they were healthcare professionals, with prior knowledge of, or colleagues working in, rehabilitation services. A third group of People with LC reported a need for LC rehabilitation services in their health board area, suggesting a lack of services and/or their promotion.

The three participants who had received rehabilitation for LC were generally positive about their experience, reporting benefits from specific professions (e.g., physiotherapy, speech and language therapy) and interventions (e.g., breathing exercises), with information and advice on LC and symptom management, particularly the use of pacing, being highly valued.

> *The biggest help was speaking to Speech and Language... she gave me lots of information that was very interesting, and lots about the biology of what's going on with my* [laryngeal] *spasms.* [DPwLC04]

For peer review only

> *Just having somebody to help you manage what that should look like, what is too much, because you can read about pacing, you can chat about it online with other people with Long COVID, but trying to get a model that fits for you as an individual is actually really hard without support.* [DPwLC06]

This contrasted with the views of People with LC of generic self-management booklets, which were commonly reported as lacking person-centredness.

> *When I did refer myself to the* [specialist] *team, I got a booklet, a massive booklet through the post, that says this that and the other. But it's such an individual, highly differentiated set of symptoms that any one person can have, just none of it was particularly relevant to me.* [DPwLC01]

One participant had sought informal rehabilitation from a personal trainer but felt that it was not helpful due to its intensity, feeling that the trainer did not have the requisite LC knowledge to support individuals effectively.

> *"Just with having this discussion it's like a wee light bulb moment that I'm having, that I'm thinking I've tried the PT* [personal training]*, it was too intense, threw the towel in."* [DPwLC01]

Despite the lack of knowledge of the potential role of rehabilitation demonstrated by some GPs, most reported that they would engage with a dedicated LC rehabilitation service, as it would provide an onward referral route for patients, particularly as they commonly reported being limited by secondary care referral criteria. Several GPs felt that a multidisciplinary team approach could be beneficial for their patients and could provide the validation that patients needed. Some also believed that earlier pulmonary rehabilitation could contribute to better functional recovery.

> *I think they feel quite isolated actually and I think it would be useful even if objectively…there's not a huge improvement. I think psychologically it would be really important for them. Someone to believe them, to see what's happening, and just thinking someone's looking out for them.* [BGP01]

For peer review only

466 People with LC reported accessing a range of other LC services and sources of support. For some, occupational health services were helpful in supporting return to work. Support from a charity was reported to be useful for some, whilst others found it too generic for their needs. Some participants found support from peers with similar experience of LC was helpful and provided validation.

Some people with LC accessed positive peer support via social media. However, others reported negative experiences and safety concerns regarding social media. The lack of monitoring in online forums contributed to some people feeling vulnerable to receiving incorrect information and negativity from others.

Many people with LC reported resorting to online information, with some taking this information to their GP consultation. Finally, as reported above, some turned to private healthcare in response to access issues and long waiting times, including psychological therapy, GP, medical specialties, and physiotherapy.

DISCUSSION

We explored the issue of access to community rehabilitation for LC from the perspectives of people with LC and GPs in four Scottish health boards. We identified several systemic challenges for LC service delivery which related to access, siloed services, limited resources, and a perceived lack of holistic care, causing frustration for both GPs and people with LC. Similar challenges have been reported in the international qualitative literature from the perspectives of people with LC[30-32] and healthcare professionals.[33] To our knowledge, this is the first study to focus on community rehabilitation in the UK. Although a minority of GPs expressed scepticism about LC and the need for rehabilitation and other services for this patient group, people with LC and most GPs agreed on the need for accessible, person-centred services and support, in keeping with previous research recommending collaborative support mechanisms[17] and improved care coordination for people with LC[31] and recent findings on accessing LC rehabilitation in Canada.[34]

For peer review only

Regarding community rehabilitation specifically, we found that: i) some people with LC and some GPs lacked knowledge on the potential role of community rehabilitation in the management of LC; ii) having prior knowledge of rehabilitation or being a healthcare professional appeared to facilitate access to community rehabilitation, and iii) people with LC who had received rehabilitation generally found it beneficial. Due to the lack of knowledge and difficulty accessing rehabilitation however, people with LC had accessed a range of other services and sources of support, with varying success.

The negative impact of LC on the health and quality of life of our study participants is in keeping with other studies[35] and demonstrates the influence of LC on a sample living in Scotland almost 3-years on from the start of the pandemic. The persisting prevalence and impact of LC on people's lives further emphasises the need for support and services such as community rehabilitation to be available, in keeping with recommendations and research findings.[1, 5, 7, 36, 37]

People with LC should have access to personalised and multidisciplinary rehabilitation[1] and such rehabilitation is reportedly available throughout Scotland[12] delivered by a variety of service models. This study highlights some potential reasons for the mismatch between recommendations, reported service availability and low numbers of people with LC accessing community rehabilitation.[15]

Lack of GP knowledge regarding community rehabilitation and its potential role in LC may, in part, be attributed to the nature of LC as a new condition that health professionals are still learning how to manage. Previous research has reported a lack of GP understanding of the role of rehabilitation professionals in the management of conditions commonly encountered in primary care; for example, physiotherapists role in osteoarthritis management.[38] Internationally, public understanding of the role of occupational therapy has been reported as limited.[39] Therefore, in the context of a new condition with an evolving evidence-base, it is perhaps not surprising that GPs and people with LC may have limited understanding of what rehabilitation professionals can offer to this patient population. The reluctance to promote the availability of services in

For peer review only

some areas, due to pre-existing resourcing and anticipated demand-capacity issues may also have limited access to LC rehabilitation.[15]

Issues with promotion of services, clarity of pathways and interdisciplinary communication between GPs and rehabilitation professionals have been reported previously.[38, 40] The findings of this study suggest that further improvements in communication and collaborative working[35] may be required to enhance access to community rehabilitation for people with LC. Indeed, the finding that prior knowledge of rehabilitation, and being a healthcare professional facilitated access to community rehabilitation is further evidence that successfully navigating the referral system is challenging.

The findings of people with LC who had managed to access community rehabilitation being satisfied with it, and GPs wanting a LC service to refer patients to are in keeping with previous research.[19] The challenge not only lies in availability of such services, but clearly in people with LC and GPs awareness of the benefits of these services, their active promotion, and clear and timely accessibility of rehabilitation services. There is therefore a need to overcome the systemic challenges to accessing timely rehabilitation reported in this study. Considering the ongoing nature of living with LC, these challenges are likely to continue beyond the time and resource constraints of Government funding provision for existing LC rehabilitation within rehabilitation services that were already historically underfunded and considered ‘Cinderella services’.[15]

Lack of capacity in the UKs rehabilitation services is not a new phenomenon[41] but it has arguably been further highlighted since COVID-19[42] Worldwide, there is a renewed focus on the importance of rehabilitation, and the size of the unmet need.[8, 43] Similar findings would arguably have emerged if this study had focussed on people with other chronic conditions suitable for community rehabilitation such as fibromyalgia, ME/CFS, cardiovascular disease and stroke. Whilst focussed on LC, this study provides additional evidence of the need for increasing capacity in community rehabilitation services.[42]

For peer review only

Strengths and limitations

This study explored the issue of accessing LC community rehabilitation in the Scottish context and included the perspectives of those referring and potentially being referred. One researcher conducted all interviews to ensure consistency and multiple researchers were involved in analysing and interpreting the data, including people with lived experience of LC. There are, however, some limitations.

The sample was one of convenience which limits generalization, and although we recruited to target for the GP sample, there was under-representation from the two health boards with dedicated LC services. Therefore, the data largely represents the views of GPs from health boards where LC services were integrated into existing community rehabilitation services; it is possible that GPs views of dedicated LC services may be different.

We recruited a mostly female sample of people with LC, and mostly from health boards with integrated or a halted dedicated LC service. Their views on accessing LC services may therefore have been biased. Although LC has been reported as more common in females,[44] the under-representation of males in this study, as with others in the field,[17] is a limitation that needs to be addressed in future research. Our sample size was small, but similar to previous studies exploring LC and its management.[17, 33, 36] Recruitment of people with LC was likely limited by our reliance on social media and one LC charity; however, both mechanisms had the potential to reach many people.

We cannot claim data saturation. However, we are confident that we achieved adequate data sufficiency[41] for the findings to reflect some of the key issues within each participant group. The perspectives of men with LC and people accessing dedicated LC rehabilitation services require further exploration. [45]We conducted interviews online with one by telephone, which may have affected rapport and depth of interaction, but provided flexibility for participants and the research team. The GP data was not transcribed, which is commonly seen as a routine step in qualitative studies. Finally,

For peer review only

participants did not check the transcripts or findings, but healthcare professionals and people with LC did comment on the findings at an open workshop.

Implications for practice and research

There is a need for greater understanding by the public, GPs, and other potential referrers of the role of community rehabilitation professionals in the management of LC. There is an equally important need for community rehabilitation services to be well promoted and accessible to the people with LC for whom they may be appropriate. Long COVID is still a prevalent condition whose impact on individuals can be profound. The need for community rehabilitation for people with LC is likely to persist. Service providers should therefore consider availability and accessibility of LC rehabilitation and ensure adequate interprofessional communication and collaboration to enhance the experience for people with LC.

CONCLUSION

We have provided further understanding of the barriers to accessing LC community rehabilitation in the Scottish context by exploring the perceptions and experiences of key stakeholders in the referral process. These findings can be used by those (re)designing community rehabilitation services for people with LC and potentially for other long-term conditions. There remains a need for greater public and GP awareness of the role of rehabilitation professionals in LC.

AUTHOR CONTRIBUTIONS

KC, ED, JM, LA, JP, JO, AL, PS contributed to the study’s conception and design. EHW undertook data collection. ED, KC, EHW, TT, JC, JS, ES undertook analyses and drafted the first version of the manuscript. KC led future iterations of the manuscript. All authors read and commented on the manuscript and approved the final version of it. The corresponding author attests that all authors meet authorship criteria and that nobody meeting the criteria have been omitted. ED and KC have joint responsibility for the

For peer review only

research conduct of the study, had access to the data, and controlled the decision to publish.

COMPETING INTERESTS

All authors have completed the ICMJE uniform disclosure form at www.icmje.org/coi_disclosure.pdf and declare: all authors had financial support from the Chief Scientist Office Scotland (grant number COV/LTE/20/29) for the submitted work; ED has received research grants from NIHR and Scottish Government for the following studies: Caring for Long COVID in primary care and DBI COVID study respectively. JM has received funding for LOCOMOTION (Long COVID multidisciplinary consortium: optimising treatments and services across the NHS). No other relationships or activities that could appear to have influenced the submitted work are declared by any of the authors.

FUNDING STATEMENT

This work was supported by the Chief Scientist Office Scotland, grant number COV/LTE/20/29

DATA AVAILABILITY

All data produced in the present study are available upon reasonable request. Robert Gordon University holds the copyright for the full interview transcripts and may grant data sharing permission on request.

ETHICS APPROVAL

Ethical approval was granted from the Wales Research Ethics Committee 6 [21/WA/0118 A03], and each of the four Health Boards granted R&D management approval.

ACKNOWLEDGEMENTS

We are grateful to all our participants who gave of their time to take part in interviews. We would like to thank Amanda Cardy, Northeast coordinator NRS Primary Care Network

For peer review only

627 and local principal investigators Lynne Frew, Lynn Morrison, and Gail Thomson-Patel for
628 support with participant recruitment.

For peer review only

REFERENCES

1. NICE. COVID-19 rapid guideline: managing the longterm effects of COVID-19, 2022.
2. World Health Organization. With the international public health emergency ending, WHO/Europe launches its transition plan for COVID-19. 2023 [Available from: https://www.who.int/europe/news/item/12-06-2023-with-the-international-public-health-emergency-ending--who-europe-launches-its-transition-plan-for-covid-19 accessed 5 October 2023.
3. Office for National Statistics (ONS). Prevalence of ongoing symptoms following coronavirus (COVID-19) infection in the UK: 30 March 2023. 2023 [Available from: https://www.ons.gov.uk/peoplepopulationandcommunity/healthandsocialcare/conditionsanddiseases/bulletins/prevalenceofongoingsymptomsfollowingcoronaviruscovid19infectionintheuk/30march2023 accessed 5 October 2023.
4. World Health Organization. Clinical management of COVID-19: living guideline, 13 January 2023. Geneva: World Health Organization, 2023.
5. Heeney A, Connolly SP, Dillon R, et al. Post-COVID care delivery: The experience from an Irish tertiary centre's post-COVID clinic. *PLoS One* 2023;18(8):e0289245. doi: 10.1371/journal.pone.0289245 [published Online First: 20230811]
6. DeMars J, Brown DA, Angelidis I, et al. What is Safe Long COVID Rehabilitation? *J Occup Rehabil* 2023;33(2):227-30. doi: 10.1007/s10926-022-10075-2
7. Decary S, De Groote W, Arienti C, et al. Scoping review of rehabilitation care models for post COVID-19 condition. *Bull World Health Organ* 2022;100(11):676-88. doi: 10.2471/BLT.22.288105 [published Online First: 20221003]
8. World Health Organization. Rehabilitation. 2023 [Available from: https://www.who.int/news-room/fact-sheets/detail/rehabilitation accessed 28 September 2023.
9. Clauw DJ, Calabrese L. Rheumatology and Long COVID: lessons from the study of fibromyalgia. *Annals of the Rheumatic Diseases* 2024;83(2):136-38. doi: 10.1136/ard-2023-224250
10. Wade DT. Rehabilitation after COVID-19: an evidence-based approach. *Clinical Medicine* 2020;20(4):359-64. doi: https://doi.org/10.7861/clinmed.2020-0353
11. Dean E. What happens inside a long covid clinic? *BMJ* 2023;382:p1791. doi: 10.1136/bmj.p1791
12. Duncan E, Cooper K, Cowie J, et al. A national survey of community rehabilitation service provision for people with long Covid in Scotland. *F1000Res* 2020;9:1416. doi: 10.12688/f1000research.27894.2 [published Online First: 20201207]
13. Baz SA, Fang C, Carpentieri JD, et al. 'I don't know what to do or where to go'. Experiences of accessing healthcare support from the perspectives of people living with Long Covid and healthcare professionals: A qualitative study in Bradford, UK. *Health Expect* 2023;26(1):542-54. doi: 10.1111/hex.13687 [published Online First: 20221213]
14. Ladds E, Rushforth A, Wieringa S, et al. Persistent symptoms after Covid-19: qualitative study of 114 "long Covid" patients and draft quality principles for services. *BMC Health Serv Res* 2020;20(1):1144. doi: 10.1186/s12913-020-06001-y [published Online First: 20201220]
15. Duncan E, Alexander L, Cowie J, et al. Investigating Scottish Long COVID community rehabilitation service models from the perspectives of people living with Long COVID and healthcare professionals: a qualitative descriptive study. *BMJ Open* 2023;13(12):e078740. doi: 10.1136/bmjopen-2023-078740
16. Kingstone T, Taylor AK, O'Donnell CA, et al. Finding the 'right' GP: a qualitative study of the experiences of people with long-COVID. *BJGP Open* 2020;4(5) doi: 10.3399/bjgpopen20X101143 [published Online First: 20201215]
17. Thomas C, Faghy MA, Owen R, et al. Lived experience of patients with Long COVID: a qualitative study in the UK. *BMJ Open* 2023;13(4):e068481. doi: 10.1136/bmjopen-2022-068481 [published Online First: 20230426]

For peer review only

18. Skilbeck L, Spanton C, Paton M. Patients' lived experience and reflections on long COVID: an interpretive phenomenological analysis within an integrated adult primary care psychology NHS service. *J Patient Rep Outcomes* 2023;7(1):30. doi: 10.1186/s41687-023-00570-2 [published Online First: 20230320]

19. Brennan A, Broughan J, McCombe G, et al. Enhancing the management of long COVID in general practice: a scoping review. *BJGP Open* 2022;6(3) doi: 10.3399/BJGPO.2021.0178 [published Online First: 20220928]

20. Bachmeier BE, Holzle S, Gasser M, et al. How do German General Practitioners Manage Long-/Post-COVID? A Qualitative Study in Primary Care. *Viruses* 2023;15(4) doi: 10.3390/v15041016 [published Online First: 20230420]

21. Tong A, Sainsbury P, Craig J. Consolidated criteria for reporting qualitative research (COREQ): a 32-item checklist for interviews and focus groups. *Int J Qual Health Care* 2007;19(6):349-57. doi: 10.1093/intqhc/mzm042 [published Online First: 20070914]

22. Scottish Government. Scottish Government Urban Rural Classification 2020. 2022 [Available from: https://www.gov.scot/publications/scottish-government-urban-rural-classification-2020/ accessed 05 October 2023.

23. Krouwel M, Jolly K, Greenfield S. Comparing Skype (video calling) and in-person qualitative interview modes in a study of people with irritable bowel syndrome – an exploratory comparative analysis. *BMC Medical Research Methodology* 2019;19(1):219. doi: 10.1186/s12874-019-0867-9

24. Sah LK, Singh DR, Sah RK. Conducting Qualitative Interviews using Virtual Communication Tools amid COVID-19 Pandemic: A Learning Opportunity for Future Research. *JNMA J Nepal Med Assoc* 2020;58(232):1103-06. doi: 10.31729/jnma.5738 [published Online First: 20201231]

25. Keen S, Lomeli-Rodriguez M, Joffe H. From Challenge to Opportunity: Virtual Qualitative Research During COVID-19 and Beyond. *Int J Qual Methods* 2022;21:16094069221105075. doi: 10.1177/16094069221105075 [published Online First: 20220604]

26. Halcomb EJ, Davidson PM. Is verbatim transcription of interview data always necessary? *Appl Nurs Res* 2006;19(1):38-42. doi: 10.1016/j.apnr.2005.06.001

27. Hill Z, Tawiah-Agyemang C, Kirkwood B, et al. Are verbatim transcripts necessary in applied qualitative research: experiences from two community-based intervention trials in Ghana. *Emerg Themes Epidemiol* 2022;19(1):5. doi: 10.1186/s12982-022-00115-w [published Online First: 20220628]

28. Gale NK, Heath G, Cameron E, et al. Using the framework method for the analysis of qualitative data in multi-disciplinary health research. *BMC Med Res Methodol* 2013;13:117. doi: 10.1186/1471-2288-13-117 [published Online First: 20130918]

29. Goldsmith LJ. Using Framework Analysis in Applied Qualitative Research. 2021

30. Brehon K, Miciak M, Hung P, et al. "None of us are lying": an interpretive description of the search for legitimacy and the journey to access quality health services by individuals living with Long COVID. *BMC Health Serv Res* 2023;23(1):1396. doi: 10.1186/s12913-023-10288-y [published Online First: 20231212]

31. MacEwan SR, Rahurkar S, Tarver WL, et al. Patient Experiences Navigating Care Coordination For Long COVID: A Qualitative Study. *Journal of General Internal Medicine* 2024 doi: 10.1007/s11606-024-08622-z

32. Schmachtenberg T, Königs G, Dragaqina A, et al. “There is no one who helps you with it”: experiences of people with long COVID regarding medical care, therapeutic measures, and barriers in the German healthcare system: results of a qualitative study with four focus groups. *BMC Health Services Research* 2023;23(1):1160. doi: 10.1186/s12913-023-10170-x

33. Horlick S, Krysa JA, Brehon K, et al. Exploring Rehabilitation Provider Experiences of Providing Health Services for People Living with Long COVID in Alberta. *International Journal of Environmental Research and Public Health* 2023; 20(24).

For peer review only

34. Hung P, Brehon K, Miciak M, et al. "I Had to Know About It, I Had to Find It, I Had to Know How to Access it": Experiences of Access to Rehabilitation Services Among People Living with Long COVID. *Physiotherapy Canada* 2024;76(1):8-24. doi: 10.3138/ptc-2023-0021
35. Rofail D, Somersan-Karakaya S, Choi JY, et al. Thematic analysis to explore patients' experiences with long COVID-19: a conceptual model of symptoms and impacts on daily lives. *BMJ Open* 2024;14(1):e076992. doi: 10.1136/bmjopen-2023-076992 [published Online First: 20240117]
36. Al-Jabr H, Windle K, Clifton A, et al. Patient experiences of the Long COVID–Optimal Health Programme: a qualitative interview study in community settings. *BJGP Open* 2024:BJGPO.2023.0137. doi: 10.3399/bjgpo.2023.0137
37. Raunkiaer M, Joergensen DS, Rasmussen A, et al. Experiences of improvement of everyday life following a rehabilitation programme for people with long-term cognitive effects of COVID-19: Qualitative study. *J Clin Nurs* 2024;33(1):137-48. doi: 10.1111/jocn.16739 [published Online First: 20230524]
38. Okwera A BscPt M, May S Ma GDPFMP. Views of general practitioners toward physiotherapy management of osteoarthritis-a qualitative study. *Physiother Theory Pract* 2019;35(10):940-46. doi: 10.1080/09593985.2018.1459987 [published Online First: 20180416]
39. Rahja M, Laver K. What does the Australian public know about occupational therapy for older people? A population survey. *Aust Occup Ther J* 2019;66(4):511-18. doi: 10.1111/1440-1630.12578 [published Online First: 20190410]
40. Igwesi-Chidobe CN, Bishop A, Humphreys K, et al. Implementing patient direct access to musculoskeletal physiotherapy in primary care: views of patients, general practitioners, physiotherapists and clinical commissioners in England. *Physiotherapy* 2021;111:31-39. doi: 10.1016/j.physio.2020.07.002 [published Online First: 20200711]
41. Evidence N. Rehabilitation: how can services meet demand? 2021 [accessed 29 Feburary 2024.
42. Wade DT. The future of rehabilitation in the United Kingdom National Health Service: Using the COVID-19 crisis to promote change, increasing efficiency and effectiveness. *Clinical Rehabilitation* 2021;35(4):471-80. doi: 10.1177/0269215520971145
43. Kamenov K, Mills J-A, Chatterji S, et al. Needs and unmet needs for rehabilitation services: a scoping review. *Disability and Rehabilitation* 2019;41(10):1227-37. doi: 10.1080/09638288.2017.1422036
44. Sylvester SV, Rusu R, Chan B, et al. Sex differences in sequelae from COVID-19 infection and in long COVID syndrome: a review. *Current Medical Research and Opinion* 2022;38(8):1391-99. doi: 10.1080/03007995.2022.2081454
45. Malterud K, Siersma VD, Guassora AD. Sample Size in Qualitative Interview Studies: Guided by Information Power. *Qual Health Res* 2016;26(13):1753-60. doi: 10.1177/1049732315617444 [published Online First: 20160710]

For peer review only

Figure Legend:

Figure 1: Number of GPs and People with Long COVID recruited from each health board

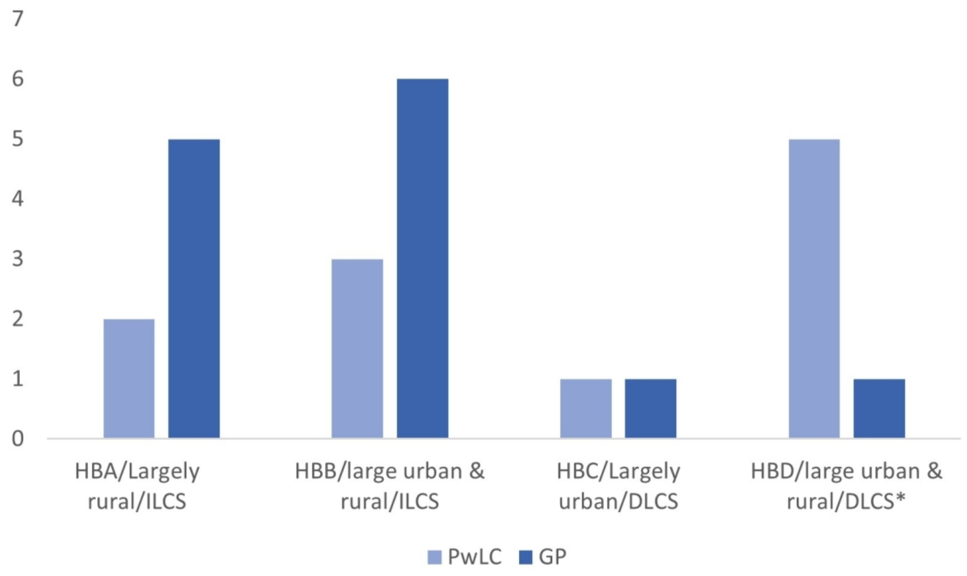

Figure 1: Number of GPs and People with Long COVID recruited from each health board

127x77mm (330 x 330 DPI)

IRAS ID: 295672

Protocol

1.0 Title

Evaluating long-covid rehabilitation in Scotland

2.0 Summary

Studies estimate that 10–35% of people with COVID-19 experience disabling clinical symptoms following the acute phase of their illness (long-covid). Given the prevalence of COVID-19 in Scotland, there is assumed to be a significant number of people experiencing long-covid, and they are starting to be referred to rehabilitation specialists. Community rehabilitation, delivered by physiotherapists, occupational therapists and other health professionals, is a well-established approach to enabling people to maximise their quality of life and recovery. However, community rehabilitation for people with long-covid is in its infancy. There are considerable differences in how community rehabilitation for people with long-covid is currently delivered across Scotland. We want to assess which models of community rehabilitation are most appropriate, in which circumstances. We will evaluate the delivery and outcomes of four different models of community rehabilitation for people with long-covid currently being delivered in Scotland. This will help us to identify which models are more suitable, for which patient groups and in which contexts. We will then hold online workshops with community rehabilitation managers, service leads and others from across Scotland. During the workshops, we will present our findings and support participants to develop evidence-based action plans to improve their local long-covid community rehabilitation services

3.0 Background

3.1 Introduction

This study is an in-depth exploration of models being used to deliver rehabilitation for longer-term effects of COVID-19 in Scotland. Understanding and evaluating how service models are developing, in parallel with evolving knowledge of the impact of COVID-19 on patients, will inform the development of new and responsive patient-centred models that can effectively meet patient and service need. This work is vital. As evidence emerges on the epidemiology and consequences of COVID-19, its impact on individuals and society, and potential burden on health services (1), strategies are required to address the long-term effects of COVID-19. The Scottish Government's Framework for Supporting People through Recovery and Rehabilitation during and after the COVID-19 Pandemic (2) states that "everyone with rehabilitation needs…will be able to access the care and support they need to live well, on their own terms." It also highlights the important role of digital solutions. This research will develop implementable recommendations for post-COVID-19 rehabilitation, congruent with the principles of the Scottish Rehabilitation Framework and Digital Health and Care Strategy (3), and that meet patients' needs.

For peer review only - http://bmjopen.bmj.com/site/about/guidelines.xhtml

IRAS ID: 295672

3.2: Long-covid

Long-term effects of COVID-19, or "long-covid" has received much recent media attention with action groups calling for more support (4). With no agreed definition, we will use that proposed by Greenhalgh et al (5) of symptoms extending 3-weeks beyond onset (post-acute COVID-19) and 12-weeks beyond onset (chronic COVID-19). Studies on long-term consequences of COVID-19 infection are ongoing; however, it is estimated that 10 – 35% of patients may experience post-acute COVID-19 (5-7). Based on Scottish Government figures for positive COVID-19 tests and survival to date (28th February 2021) this equates to between 19,367 and 67,784 people in Scotland with post-acute COVID-19, with around 2,000 remaining significantly unwell at 12-weeks, commonly due to organ damage (7). Long-covid also affects people with milder symptoms and those who were not hospitalised (6). Rehabilitation therefore needs to be accessible to those presenting in community settings as well as being discharged from hospital.

Reported symptoms vary widely and commonly include respiratory, cardiopulmonary, neurological, musculoskeletal and mental wellbeing sequelae, as well as fatigue and loss of taste and smell (5,6,8). As described above, presentation and severity are variable, and it is important to note that many patients with long-covid will have co-existing conditions that will impact on symptoms and require dual management. Rehabilitation therefore needs to be multidisciplinary, comprehensive, and tailored to patients' needs, in order to maximise function, quality of life and participation in society (9).

3.3: Long-covid rehabilitation

Rehabilitation for long-covid is in its infancy, reflected in a recent living systematic review update finding predominantly expert opinion, indicating that high-quality research is required (10). Research on rehabilitation for other respiratory conditions *may* be generalisable to the COVID-19 population (11), but this is yet to be determined. Several UK expert bodies and published expert opinion recommend a stepped, needs-based rehabilitation approach incorporating information provision, self-management support and specialist services as required. They also recommend that rehabilitation should be individualised and progressive and the use of digital solutions optimised. *How* rehabilitation can be optimally delivered is not known but vital to determine, given that rehabilitation services need to cope with additional COVID-19 demand whilst continuing to provide rehabilitation for other, often vulnerable, patient populations (12). By investigating different ***models of community rehabilitation*** currently implemented in Scotland to evaluate what works, where, with whom and how, we will develop recommendations on *how* rehabilitation can be best delivered across Scotland to meet the needs of patients and services, thereby developing new responsive models of rehabilitation.

4.0 Aims & research questions

The overarching aim of this research is to provide responsive evidence-based

For peer review only - http://bmjopen.bmj.com/site/about/guidelines.xhtml

recommendations to the NHS about how to most effectively deliver community rehabilitation for people with long-covid. We will do this by evaluating the implementation and impact of different emerging models of long-covid rehabilitation in different contexts in Scotland. We will address the following specific research questions:

1. How is rehabilitation for long-covid being delivered in 4 Scottish health boards?
2. What are the barriers and facilitators to implementing long-covid rehabilitation from managerial, rehabilitation staff, and patients' perspectives and experiences?
3. What are the perceptions and experiences of community-dwelling adults with long-covid on accessing rehabilitation for long-covid?
4. What are GPs perceptions and experiences of managing community dwelling adults presenting with symptoms of long-covid that may be suitable for rehabilitation?
5. What are the outcomes of different community rehabilitation models for people experiencing long-covid?
6. What are the costs associated with long-covid rehabilitation in the 4 selected cases?
7. In what ways are the selected long-covid community rehabilitation models successful (or not)?
8. How can we use the knowledge from 1-5 above to inform the development of responsive patient-centred models of rehabilitation that can be adapted to suit the needs of patients and services across Scotland?

5.0 Methodology & methods

We will conduct a mixed-methods case study realist evaluation (RE) of different models of community rehabilitation, in different contextual settings, for people experiencing long-covid in order to understand what works, for whom, in what circumstances and how. The study is informed by the RE framework (13), which is used to study the process and context of 'implementation' and to identify the 'mechanisms of action' that lead to successful (or unsuccessful) delivery. It will also be used to investigate how interventions are being implemented in each case study area. Understanding the local contexts and mechanisms of action will enable us to explain observed outcomes in the different models of community rehabilitation delivery for people with long-covid. Realist Evaluation is a highly appropriate method to use to study the delivery and impact of emerging models of care for long-covid. Realist evaluation emerged in response to the need for knowledge that extends beyond that obtained by controlled trial design (is the intervention effective?) to answering questions of what works for whom and in what circumstances. It is 'realist' in the sense that it does not deny the relevance of robust measurement of outcomes, but also seeks to take account of the context in which interventions are delivered and to identify the mechanisms that make programmes work (or not) to produce the observed outcomes. It is not the interventions themselves that work but the ability and willingness of participants to engage with them.

For peer review only - http://bmjopen.bmj.com/site/about/guidelines.xhtml

Setting

Rehabilitation services in four Scottish Health Boards: NHS Ayrshire & Arran, NHS Grampian, NHS Lanarkshire, NHS Tayside. The boards have been selected to represent a diversity of models of rehabilitation in terms of mode of delivery (digital/face-to-face/blended/self-management) and whether they provide a new long-covid service or integrate long-covid rehabilitation into existing services. This information was gained by a Scottish-wide survey of long-covid rehabilitation conducted by the study team (14). The selected NHS boards also represent a range of geographical areas in relation to urban/rural classification, to allow for transferability of findings across Scotland, and boards with sufficient covid-19 cases to make recruitment viable.

The study consists of 2 work packages (WP):

WP1: Case study RE to measure implementation and outcomes of different service models of community rehabilitation for people experiencing long-covid. Within WP1 we will conduct:

I. Process evaluation addressing research questions 1-4

Data will be collected at 4 time-points over a 12-month period across each case to understand local contexts and decisions regarding delivery of community rehabilitation for people with long-covid. Data collection rounds 1 to 4 will describe the long-covid models of community rehabilitation and track local decisions on how services are implementing and developing their long-covid rehabilitation service. Data collected during rounds 1 to 4 will elicit rationales for service delivery decision making and how implementation is supposed to work. Data collection will be obtained from online focus groups (with people who have received long-covid community rehabilitation), and online individual interviews (6-10 per case per round) with managers/service leads/consultants/ and rehabilitation staff with potential to deliver long-covid community rehabilitation. Data from Round 1 will specifically be used to elicit rationales regarding local decisions, identify 'folk theories' surrounding how community rehabilitation delivery is supposed to work, and identify hypothesised mechanisms of (successful) change/implementation. This will establish the locally hypothesised context-mechanism-outcome (CMO) configurations which will then be tested using data gathered in Rounds 2-4.

Participants

1. People who have received long-covid rehabilitation (patients), managers/service leads, and professionals delivering long-covid rehabilitation will be recruited. See table 1 for inclusion/exclusion criteria.
2. Community dwelling adults with long-covid (i.e., not currently receiving community rehabilitation) and GPs in each of the four health boards.

Recruitment

Patients will be sent a study pack in one of the following ways: (i) with their long-covid appointment if it is mailed out to them; in-person from their rehabilitation professional when they receive their first session (either in their home or in a community rehabilitation setting); by mail if their initial session is held virtually. The study pack will consist of: i) letter of invitation, ii) participant information sheet, iii) baseline measures. Patients will therefore be identified by administrative or clinical staff, and will opt-in to the study by completing the

For peer review only - http://bmjopen.bmj.com/site/about/guidelines.xhtml

IRAS ID: 295672

baseline measures (paper-based or online) and returning them to the research team. Participants will opt-in to taking part in a focus group, and if they wish to do so will provide contact details for the study team to contact them.

Table 1: Inclusion/exclusion criteria participants

	**Inclusion**	**Exclusion**
**Patients**	-Aged 18+<br>-COVID-19 diagnosis (with or without positive covid-19 test) >3-weeks previously<br>-Referred due to presenting with symptoms thought to be due to long-covid (including but not limited to: respiratory, fatigue, musculoskeletal, mental health)<br>-Suitable for rehabilitation (determined by rehabilitation staff)<br>-Able to understand written & spoken English<br>-Willing to take part in study	-Aged <18<br>-Requiring specialist (inpatient) management of specific complications<br>-Unable or unwilling to provide informed consent<br>-Other life-limiting illness with life expectancy <6-months e.g. disseminated malignancy
**Managers/service leads**	Managers/service leads of community rehabilitation services providing long-covid rehabilitation	Managers/service leads of inpatient services
**Rehabilitation professionals**	Healthcare professionals delivering long-covid rehabilitation including but not limited to, physiotherapists, occupational therapists, speech and language therapists, psychologists, medical doctors. Where relevant (i.e. involved in delivering long-covid rehabilitation) healthcare support workers will be included	-Student healthcare professionals<br>-Healthcare professionals providing in-patient rehabilitation
**Community dwelling adults with long-covid**	Aged 18+<br>Self-identify as having experience of long-covid and accessing/attempting to access healthcare services for possible rehabilitation	-Aged <18<br>-Unable or unwilling to provide informed consent<br>-Other life-limiting illness with life expectancy <6-months e.g., disseminated malignancy
**GPs**	GP based in any of the four participating health boards with experience of patients with probable long-covid who	

For peer review only - http://bmjopen.bmj.com/site/about/guidelines.xhtml

	may be suitable for rehabilitation	

Rehabilitation professionals and Managers/service leads will be recruited by email with a letter of invitation and participant information sheet attached. The email will come from a relevant gatekeeper e.g. team lead/manager for rehabilitation professionals; sector lead/allied health professions lead for service leads/managers. Participants will opt-in to the study by contacting the research team by email or telephone.

Community dwelling adults with long-covid will be recruited via social media using RGU School of health sciences and NMAHP research unit University of Stirling Facebook and Twitter accounts, via long-COVID Scotland action group, and The Alliance. Following these routes, we anticipate snowball sampling will occur.
GPs will be recruited by email invitation circulated on our behalf by the NRS Primary Care Network**.**
We plan to recruit these two groups of participants for one round of data collection only, anticipating that referral to long-COVID services will pick up in the coming months. However, should this assumption be incorrect, and referrals to services and this study remain low, we will include these two groups in subsequent rounds of data collection also.

Informed consent
Patients returning baseline measures will indicate their consent to taking part in the quantitative data collection by answering a question on the baseline measures paper or electronic form. Participants will self-select to take part in the focus group by leaving their name and contact details at the end of the survey or e-mailing the research team directly. The research fellow (RF) will then contact the participant by telephone or Microsoft Teams to discuss further and if relevant make arrangements for focus group participation. Consent to take part in the focus group will be provided verbally (audio recorded) during a separate telephone or Microsoft Teams call with the RF. A consent form with standard statements will be read to participants and they will have to agree to each statement in order to take part. Rehabilitation professionals and service leads/managers will provide verbal consent prior to commencing their interviews in the same manner.

Community-dwelling adults with long-covid will contact the research team using the details provided in invitation materials. Individuals will then be provided with study information and following discussion with the RF will provide verbal consent to be interviewed as described above.

GPs will likewise contact the research team following invitation emails sent by the NRS Primary Care Research Network, and will likewise provide verbal informed consent.

Data collection

Patients: One online focus group will be conducted in each case study site during each round of data collection (n=4 focus groups of 8-10 participants each). Patients will be purposively sampled from those opting-in to a focus

For peer review only - http://bmjopen.bmj.com/site/about/guidelines.xhtml

group, based on sampling criteria including age, gender, ethnicity, deprivation, and whether they completed rehabilitation or not. In the event that sufficient diversity is not achieved by this method, rehabilitation services will be asked to conduct additional targeted recruitment on our behalf. Focus groups will take place via Microsoft Teams and will each be conducted by 2 trained researchers using a topic guide. Focus groups will last no longer than 2-hours, will include adequate rest breaks, and will be recorded via Teams. To address issues of digital exclusion, a sample of participants who are interested in taking part but unable (or unwilling) to join an online focus group will be invited to provide their views via telephone interview or a short paper-based survey.

Rehabilitation professionals and service leads/managers will take part in a 1-1 interview, conducted via Teams, supported by a topic guide and recorded in the same manner as the focus groups. From those volunteering to take part we will purposively recruit 3-5 professionals and 2 managers per site to be theoretically representative of staff at each site. We anticipate that the same staff will take part in each of the 4 rounds of data collection, unless there are changes in staffing, in which case additional recruitment will be conducted.

Community dwelling adults and GPs will take part in a single 1-1 interview, conducted by Teams, supported by a topic guide and recorded in the same manner as the focus groups. We will aim to recruit 3-5 community dwelling adults and 2-5 GPs at each site.

Data processing

Following data collection, the MP3 recordings will be downloaded by the RF and saved on secure servers at Robert Gordon University (RGU) and Stirling University (UoS) only accessible to the research team. The transcripts will be transcribed by GDPR-compliant transcribers and stored on the same secure servers.

Data analysis

Data will be analysed to articulate the context and mechanisms of action and relate these to observed (quantitative and qualitative) outcomes (CMO configurations). Each data source will be analysed individually in the first instance to reach separate conclusions. Data from meetings and interviews will be analysed using the Framework Approach (15) aided by NVivo (V12) software. For all qualitative datasets, 10% of transcripts will be coded independently by two RFs and the resulting codes discussed, refined and agreed as a final coding frame to be systematically applied to all transcripts. Following familiarisation with the data, a thematic framework will be developed and applied across the data set. Data will then be tabulated and conceptual maps used to make links between themes. Data will be used to construct case summaries at the level of the different modes of delivery and across different sites. All data for each mode of delivery and for each delivery site will be collected together and consistencies/ inconsistencies searched for. The aim of analysis at this stage is to identify the core barriers and facilitators and mechanisms of action within each mode of delivery, the detailed explanations for them and interactions between them.

For peer review only - http://bmjopen.bmj.com/site/about/guidelines.xhtml

IRAS ID: 295672

II. Outcome evaluation and cost-consequence analysis addressing outcomes 5-7

Outcomes data will be collected before and after people receive community rehabilitation in each location/model of service delivery to establish whether improvements are observable. Recruitment of up to 100 people per case study site will be conducted, from month 3 to month 18 of the study, by the staff delivering community rehabilitation in each local site. It is anticipated that each staff member (approximately 4-6 per team) will collect data on between 15-20 people over the 16-month period. The following patient demographic and clinical data will be collected by each team member for each consenting patient:

- Age
- Sex
- Ethnicity
- Occupation
- Social deprivation status in deciles (gathered using home postcode entered into https://simd.scot/#/simd2020/BTTTFTT/14/-2.7960/55.9548/
- Key presenting problems (e.g. musculoskeletal, fatigue, breathlessness, mental health)
- Co-morbidities (e.g. diabetes, hypertension, COPD)
- Smoking status
- Height & Weight (to estimate BMI)
- Duration of rehabilitation (date of first appointment to discharge)
- Number and duration of sessions
- Number and type of professionals seen

Patient outcomes will be collected at baseline immediately prior to first appointment, at the end of the last session of rehabilitation, and at 3-month follow-up. Baseline measures will be provided to patients with their appointment letter or at their first rehabilitation session (in person or by post if session is virtual), and they can complete the paper copy and return to the study team in freepost envelope or they can complete the measures online (Jisc online surveys) as a web address will also be provided. Each participant will have a unique ID allocated by the site administration or clinical staff, which will be documented on the measures. Patients will be instructed to input this unique ID if they complete the measures online. Reminders will be sent by administration/clinical staff to follow-up individuals who do not submit completed measures. Regular communication between the study RFs and case study sites will facilitate reminders and sending discharge ad follow-up measures to participants.

Outcome measures

In keeping with the PHOSP study[1], we include the following outcomes:

[1] http://www.isrctn.com/ISRCTN10980107

For peer review only - http://bmjopen.bmj.com/site/about/guidelines.xhtml

Primary Outcome

Quality of Life: EQ-5D-5L utility index and visual analogue scale (VAS).

Secondary Outcomes

Mental Health

- Generalised Anxiety Disorder Assessment (GAD-7)
- Patient Health Questionnaire (PHQ-9)
- Post-Traumatic Stress Disorder Checklist for DSM-5 (PCL-5)

Clinical symptoms

- Dyspnoea12 Questionnaire
- FACIT – Fatigue Questionnaire

Generic

- Nottingham Extended Activities of Daily Living Scale
- General Practice Physical Activity Scale

Covid Specific

Post-COVID-19 Functional Status (PCFS)

Service-related data

In addition to the data collected from consenting patients, rehabilitation staff will collect the demographic and clinical data listed above (age, ethnicity, occupation etc.) for all long-covid referrals during the study period. This will allow us to determine the extent to which our sample is representative of the long-covid population in the four study sites.

Data analysis

Analysis of quantitative data will be address research aims: 3 (What are the outcomes of different community rehabilitation models for people experiencing long-covid?) and 4 (What are the costs associated with long-covid rehabilitation in the 4 selected cases?) Descriptive statistics will be tabulated for all outcomes at each time point, showing means and SDs. To describe representative changes in each outcome measured, generalized linear mixed models will be conducted. Mixed effects models will be used to account for the repeated measures nature of the data and appropriately address missing data without requirement for listwise deletion. Random intercepts and slopes will be included to quantify inter-individual variability in baseline measures and response to rehabilitation as well as general trends. Where suggested by the data and model checking processes, autoregressive models and non-normal link functions will be included to obtain more accurate parameter estimates and their associated standard errors. Addition of fixed effects into models will be included to investigate systematic differences associated with demographic factors (e.g. age, ethnicity, social deprivation status) and study sites where appropriate statistical power exists. Standard model building (e.g. modelling the effects of time [within-person fluctuation to within-person change over time], modelling the effects of predictors [time-invariant predictors to time-varying predictors in models of

For peer review only - http://bmjopen.bmj.com/site/about/guidelines.xhtml

within-person fluctuation and change]) and model checking (e.g. likelihood ratio tests and residual analyses) processes will be used throughout, with all analyses performed using the lme4 package in the statistical environment R.
The primary outcome is general health status measured via the EQ-5D-5L utility index and VAS. The EQ-5D-5L has been selected to differentiate smaller changes in health status and is less prone to ceiling effects (16). Based on NICE recommendations, quality-adjusted life-years will be calculated using tariffs specific to the EQ-5D-5L and will be mapped for reference-case analyses using the function developed by van Hout (17).
The costs associated with long-covid rehabilitation will be quantified through mean values for all outcomes presented for each study site with uncertainty estimated through boot-strapping procedures. Costs incurred at each site from the perspective of the NHS will be presented and will include estimated costs of the differing modes of delivery (digital, self-management and face to face). Unit costs will be obtained and calculated from Public Health Scotland's Cost Book for outpatient allied health professional clinics (R044X), summary of expenditure on community staff and supplies (R550), and community staff WTE and pay (SFR 8.1). ***Statistical Power:*** Estimates of statistical power were based on the primary outcomes and previous research of the minimum important difference for people with COPD. Multilevel mixed effects models were simulated in R using population parameters obtained from (16) and a correlation of 0.5 between repeated measures. With a two-sided alpha = 0.05, power to identify a common minimum important difference across the 4 study sites at both post-intervention and 3-month follow-up was estimated as 0.54, 0.74 and 0.87, for sample sizes of 60, 80 and 100, respectively.

III. Synthesis of process, outcomes, and cost consequence analyses addressing research question 8

Realist data synthesis will be undertaken. The findings from individual data sources (including participant outcome measures and data from interviews) will be presented in matrices that bring together key issues from the different analyses, to facilitate drawing overall conclusions about the mechanisms of change within each context, which components and mechanisms are most important, and how these might explain observed outcomes or unanticipated outcomes. A concurrent triangulation mixed methods design will be used (18), whereby both quantitative and qualitative data are considered simultaneously. Initially data from each source will be analysed separately (to reach separate conclusions) and then there will be cross-method analysis to reveal meta-inferences that will inform conclusions related to implementation models, context, mechanisms and outcomes (19).

WP2: "Dissemination for Implementation" workshops addressing research question 6. Two "Dissemination for Implementation" virtual workshops will be held: an interim one at month 12 and a final one at month 23. These online events will consist of information sharing and also an 'action planning' component for participants to reflect on how they can integrate study findings into their ongoing service delivery. These events will also act as 'data gathering' opportunities for the study, hence their inclusion as an objective: gathering data on potential opposition and barriers to implementation, how services make sense

For peer review only - http://bmjopen.bmj.com/site/about/guidelines.xhtml

of and use our study findings and apply these to their own services. We expect the key people who lead and manage community rehabilitation services to attend and to leave with an action plan, and will use a targeted approach to promoting the workshops among the intended audience.

The workshops will be delivered according to a theoretically driven structure detailing: actions; theorised mechanisms of change; and expected outputs (See additional documentation). We have successfully used this theory driven workshop structure following both a Department of Health funded national consensus study (20) and an NIHR funded implementation study relating to implementation of a pelvic organ prolapse intervention (21). That workshop resulted in the study findings being adopted at a national level and the recommendations of the study and learning from the implementation day being implemented locally.

Workshop discussions will be audio-recorded (with permission) and/or notes will be taken by research team members on how participants translate study findings in relation to their local circumstances, any opposition or barriers, what level of local action planning is reached or reasons for non-action planning. These data will be qualitatively analysed using the Framework Approach, with specific attention to understanding levels of opposition and action, the discussion of barriers and whether the study findings influence the development of solutions to these barriers, whether study identified 'mechanisms' of change are adopted into local action plans and service delivery options, and which models of delivery seem more favourable for wider adoption and implementation across NHS Health Boards in Scotland.

6.0 Project Management

The study will be sponsored by RGU. It will be overseen by a Study Steering Committee (SSC) comprising of a Chair (independent academic researcher), one further independent academic researcher, a rehabilitation expert, two consumer representatives and the co-chief investigators (CIs). The SSC will meet quarterly. The CIs, co-applicants, and RFs will meet monthly as a Project Management Group (PMG) to consider the overall progress of the study and deal with any arising problems. Two consumer representatives will join the PMG quarterly. In addition to this, smaller sub-groups of members of the PMG will meet as required to ensure that individual elements of the study are progressing. Finally, KC and ED as Co-CIs will meet weekly with the study RFs about the day to day management of study activity. A full risk management plan will be developed prior to study commencement. This will be reviewed quarterly, during our PMG meeting. The project team will adhere to General Data Protection Regulation (GDPR) standards of data protection and data storage.

7.0 Ethical issues

The study will collect data from patient completed outcome measures, anonymous data on long covid rehabilitation service usage, and qualitative data from staff and people who have received long covid rehabilitation.

For peer review only - http://bmjopen.bmj.com/site/about/guidelines.xhtml

IRAS ID: 295672

The populations being studied are adults (aged > 18 years) and NHS rehabilitation professionals. Potential participants will be either experiencing symptoms of long-covid, or be delivering a rehabilitation service within the context of a pandemic. Both situational contexts highlight the importance of clear participant information sharing about the nature and expectation of study participation and clear signposting that participation is entirely voluntary and can be withdrawn at any point without reason. Considerable consideration has been given to the data collection procedures to ensure that it is not burdensome on individuals or services. We have worked in close collaboration with the included services and with service users to co-construct research tools and agree recruitment and participation methods that are appropriate and proportional.

One of the key ethical concerns in this study is maintaining patient privacy, data management and data security. All study data will be stored on password protected and encrypted university servers (Sharepoint). Data will be re-identifiable in that participants will be allocated a code and coded data will be processed and analysed. The code sheet containing participants' identifiable details will be stored separately and only accessed where necessary (e.g. sending invites for interviews) by nominated members of the study team.

We considered at length and sought expert advice regarding participants potentially reporting clinically worrying scores on the outcome measures, especially GAD-7, PHQ-9 and PCL-5. As the research team will not access participants' scores immediately, we are not in a position to intervene in a timely manner. Therefore, we have provided advice to participants about seeking help from their GP should they be concerned about their mental health after completing the measures (see participant information sheet), and we have also provided contact details for generic mental health support for all participants (also participant information sheet) as well as long-covid support. Focus group participants will also be provided with contact details for generic mental health support and long-covid support.

Having reviewed each of our study actions, we believe that risk of harm from this study is low. Notwithstanding this fact, the research team have established a study protocol to clarify communication and reporting channels if any element of the study methods proves to have significant unforeseen negative consequences. The protocol involves communicating initially with the relevant study site and forming an action plan to address the issue that has arisen. Where the unintended consequence could be relevant to other study sites, the PIs (KC, ED) will inform the pilot site leads of the situation and any suggested mitigating action that could be taken.

For peer review only - http://bmjopen.bmj.com/site/about/guidelines.xhtml

8.0 References

(1) Barker-Davies et al. The Stanford Hall consensus statement for post-COVID-19 rehabilitation. *Br J Sports Med* 2020;54:949–959.

(2) Scottish Government. Framework for Supporting People through Recovery and Rehabilitation; during and after the COVID-19 Pandemic. The Scottish Government, August 2020. Available from: file:///C:/Users/shskc/Downloads/framework-supporting-people-through-recovery-rehabilitation-during-covid-19-pandemic%20(2).pdf [Accessed 2nd November 2020].

(3) Scottish Government. Scotland's Digital Heath & Care Strategy. The Scottish Government, April 2018. Available from: https://www.gov.scot/publications/scotlands-digital-health-care-strategy-enabling-connecting-empowering/ [Accessed 2nd November 2020].

(4) Long Covid Support [online] available from: https://www.longcovid.org/ [Accessed 2nd November 2020].

(5) Greenhalgh T et al. Management of post-acute covid-19 in primary care. *BMJ* 2020;370:m3026.

(6) Tenrforde MW et al. Symptom Duration and Risk Factors for Delayed Return to Usual Health Among Outpatients with COVID -19 in a Multistate Health Care Systems Network – United States, March – June 2020. *Morbidity and Mortality Weekly Report* 2020;69(30):993-998.

(7) Greenhalgh T et al. "Long Covid": evidence, recommendations and priority research questions. Written evidence (COV0050) House of Lords Enquiry 2020.

(8) Maxwell E. Living with Covid 19. A dynamic review of the evidence around ongoing Covid 19 symptoms (often called Long Covid). NIHR Centre for Engagement and Dissemination, September 2020. Available from: https://evidence.nihr.ac.uk/themedreview/living-with-covid19/ [Accessed 2nd November 2020.

(9) Sheehy LM. Considerations for Postacute Rehabilitation for Survivors of COVID-19. *JMIR Public Health Surveill* 2020;6(2): e19462.

(10) Andrenelli E et al. Systematic rapid living review on rehabilitation needs due to COVID-19: update to May 31st 2020. *Eur J Phys Rehabil Med* 2020; 56(4):508-514.

For peer review only - http://bmjopen.bmj.com/site/about/guidelines.xhtml

IRAS ID: 295672

(11) Goodwin V et al (2021). Rehabilitation to enable recovery from COVID-19: a rapid systematic review. *Physiother 2021 (in press)* https://doi.org/10.1016/j.physio.2021.01.007

(12) Guttenbruner C et al. Why rehabilitation must have priority during and after the COVID-19 pandemic: A position statement of the global rehabilitation alliance. *J Rehabil Med* 2020;52: jrm00081.

(13) Pawson R, Tilley N. Realistic evaluation: Sage; 1997.

(14) long-covid survey

(15) Framework analysis

(13) Pawson R, Tilley N. Realistic evaluation: Sage; 1997.

(14) long-covid survey

(15) Framework analysis

(16) Nolan 2015. The EQ-5D-5: health status questionnaire in COPD: Validity responsiveness and minimum important difference.

(17) van Hout B et al. Interim scoring for the EQ-5D-5L: mapping the EQ-5D-5L to EQ-5D-3L value sets. *Value Health*. 2012 Jul-Aug;15(5):708-15.

(18) Creswell JW. Research Design: Qualitative, quantitative and mixed methods approaches. 2009 London: Sage.

(19) Teddlie CB, Tashakkori A. Foundations of Mixed Methods Research: Integrating Quantitative and Qualitative Approaches in the Social and Behavioral Sciences. 2009 London: Sage.

(20) Duncan, E et al. Consensus on items and quantities of clinical equipment required to deal with a mass casualties big bang incident: a national Delphi study. *BMC Emergency Med* 2020; 14(1): 5.

(21) Maxwell, M et al. PROPEL: implementation of an evidence based pelvic floor muscle training intervention for women with pelvic organ prolapse: a realist evaluation and outcomes study protocol. *BMC Health Services Research* 2017; 17(1):1-10.

For peer review only - http://bmjopen.bmj.com/site/about/guidelines.xhtml

IRAS ID: 295672

Appendix 1: Study Plan

Year1												
**Month:**	**1**	**2**	**3**	**4**	**5**	**6**	**7**	**8**	**9**	**10**	**11**	**12**
	Site preparation & recruitment		Qualitative Round 1					Qualitative Round 2				
				Qualitative data processing & analysis								
			Quantitative data collection, processing & analysis									
				Steering Committee				Steering Committee				Steering Committee
												Workshop 1
	PMG	PMG	PMG	PMG	PMG	PMG	PMG	PMG	PMG	PMG	PMG	PMG
**Year 2**												
**Month:**	**1**	**2**	**3**	**4**	**5**	**6**	**7**	**8**	**9**	**10**	**11**	**12**
	Qualitative Round 3					Qualitative Round 4						
	Qualitative data processing & analysis											
	Quantitative data collection, processing & analysis							Realist synthesis				Reporting
												Outputs
				Steering Committee				Steering Committee				Steering Committee
											Workshop 2	
	PMG	PMG	PMG	PMG	PMG	PMG	PMG	PMG	PMG	PMG	PMG	PMG

PMG=Project Management Group

For peer review only - http://bmjopen.bmj.com/site/about/guidelines.xhtml

IRAS ID: 295672

Version Control

Date	Version	Changes
23.03.21	1.0	N/A
07.03.22	2.0	1. Added objective 3 & 4 to explore community dwelling adults & GPs perceptions & experiences of long-covid rehabilitation<br>2. Updates participants, recruitment, consent, data collection for these additional objectives<br>3. Updated numbering of remaining objectives

For peer review only

For peer review only - http://bmjopen.bmj.com/site/about/guidelines.xhtml

Consolidated criteria for reporting qualitative studies (COREQ): 32-item checklist

Please indicate in which section each item has been reported in your manuscript. If you do not feel an item applies to your manuscript, please enter N/A.

For further information about the COREQ guidelines, please see Tong *et al.*, 2017: https://doi.org/10.1093/intqhc/mzm042

No.	Item	Description	Section #
**Domain 1: Research team and reflexivity**			
Personal characteristics			
1.	Interviewer/facilitator	Which author/s conducted the interview or focus group?	
2.	Credentials	What were the researcher's credentials? *E.g. PhD, MD*	
3.	Occupation	What was their occupation at the time of the study?	
4.	Gender	Was the researcher male or female?	
5.	Experience and training	What experience or training did the researcher have?	
Relationship with participants			
6.	Relationship established	Was a relationship established prior to study commencement?	
7.	Participant knowledge of the interviewer	What did the participants know about the researcher? *E.g. Personal goals, reasons for doing the research*	
8.	Interviewer characteristics	What characteristics were reported about the interviewer/facilitator? *E.g. Bias, assumptions, reasons and interests in the research topic*	
**Domain 2: Study design**			
Theoretical framework			
9.	Methodological orientation and theory	What methodological orientation was stated to underpin the study? *E.g. grounded theory, discourse analysis, ethnography, phenomenology, content analysis*	
Participant selection			
10.	Sampling	How were participants selected? *E.g. purposive, convenience, consecutive, snowball*	
11.	Method of approach	How were participants approached? *E.g. face-to-face, telephone, mail, email*	
12.	Sample size	How many participants were in the study?	
13.	Non-participation	How many people refused to participate or dropped out? What were the reasons for this?	
Setting			
14.	Setting of data collection	Where was the data collected? *E.g. home, clinic, workplace*	
15.	Presence of non-participants	Was anyone else present besides the participants and researchers?	

For peer review only

16.	Description of sample	What are the important characteristics of the sample? *E.g. demographic data, date*	
Data collection			
17.	Interview guide	Were questions, prompts, guides provided by the authors? Was it pilot tested?	
18.	Repeat interviews	Were repeat interviews carried out? If yes, how many?	
19.	Audio/visual recording	Did the research use audio or visual recording to collect the data?	
20.	Field notes	Were field notes made during and/or after the interview or focus group?	
21.	Duration	What was the duration of the interviews or focus group?	
22.	Data saturation	Was data saturation discussed?	
23.	Transcripts returned	Were transcripts returned to participants for comment and/or correction?	
**Domain 3: analysis and findings**			
Data analysis			
24.	Number of data coders	How many data coders coded the data?	
25.	Description of the coding tree	Did authors provide a description of the coding tree?	
26.	Derivation of themes	Were themes identified in advance or derived from the data?	
27.	Software	What software, if applicable, was used to manage the data?	
28.	Participant checking	Did participants provide feedback on the findings?	
Reporting			
29.	Quotations presented	Were participant quotations presented to illustrate the themes / findings? Was each quotation identified? *E.g. Participant number*	
30.	Data and findings consistent	Was there consistency between the data presented and the findings?	
31.	Clarity of major themes	Were major themes clearly presented in the findings?	
32.	Clarity of minor themes	Is there a description of diverse cases or discussion of minor themes?	

When submitting your manuscript via the online submission form, please upload the completed checklist as a Figure/supplementary file.

If you would like this checklist to be included alongside your article, we ask that you upload the completed checklist to an online repository and include the guideline type, name of the repository, DOI and license in the *Data availability* section of your manuscript.

Developed from: Allison Tong, Peter Sainsbury, Jonathan Craig, Consolidated criteria for reporting qualitative research (COREQ): a 32-item checklist for interviews and focus groups, International Journal for Quality in Health Care, Volume 19, Issue 6, December 2007, Pages 349–357, https://doi.org/10.1093/intqhc/mzm042

LOng COvid Rehabilitation
In Scotland: an Evaluation

IRAS ID: 295672

Study Title: Evaluating long-COVID rehabilitation in Scotland

Topic Guide for People with Long COVID

Aim: To explore community dwelling adults' perception and experiences of long-COVID and of attempting to access physical rehabilitation services

The interviews have a general purpose to obtain feedback from people with experience of long-COVID. The interviews will function in an open way to facilitate discussion of issues that are important to the individuals concerned. Therefore, the topic guide is deliberately broad and more specific areas for questioning will be driven by the interests of those taking part

General introduction

The researcher will introduce the purpose of the interview. The interviewee will provide their demographics (sex, age, employment status).

Interview questions

- Can you tell me a bit about your experience of long-COVID?
  - To put at ease, get some general background information, find out main symptoms/duration/progression

- Can you tell me about your experience of accessing healthcare for support with long-COVID?
  - Explore if/what/when/what happened/what helped or not
  - Explore if offered/accessed/tried to access rehabilitation services (e.g., community rehabilitation/physio/OT/other AHPs) and what the outcome was – if not tried to access why not
  - Explore if any barriers to accessing rehabilitation services
  - Explore if think rehabilitation services could be helpful for them or others with long-covid
  - Explore if they consider themselves to have unmet needs regarding management of long-COVID

- Is there anything else you would like to tell me about your experience of long-COVID or accessing healthcare services that you think would be useful to our study?

LOng COvid Rehabilitation
In Scotland: an Evaluation

IRAS ID: 295672

Topic Guide for GPs

Aim: To explore GPs experiences of managing people with long-covid, in particular people who have symptoms that may benefit from rehabilitation from AHPs (e.g., physiotherapists, occupational therapists, dietitians, speech and language therapists), and their experiences of long-covid rehabilitation services in their area

The interviews have a general purpose to obtain feedback from those involved in managing people with long-covid. The interviews will function in an open way to facilitate discussion of issues that are important to the individuals concerned. Therefore, the topic guide is deliberately broad and more specific areas for questioning will be driven by the interests of those taking part

General introduction

The researcher will introduce the purpose of the interview. The GP will introduce their role, years' experience as GP, and general experience of long-COVID.

Interview questions

- Do you have many patients presenting with long-COVID?
    - Explore numbers/how being "diagnosed"/presenting problems
- How are you managing patients with long-COVID?
    - Explore whether individualised or whether any trends for what patients need
    - Explore self-management/onward referral (where to)
    - Explore if perceived need for rehabilitation (e.g., for reduced exercise tolerance, breathlessness, diet, swallowing etc.)
        - If yes next question
        - If no explore why not?
- What are your perceptions of the value of services such as community rehabilitation for people presenting with long-covid symptoms?
- What community rehabilitation services are available for long-COVID patients in your practice to be referred to?
    - Explore whether aware of CRTs or individual professions (e.g., physio/OT) who may be taking referrals
    - Are there any barriers to referring patients to community rehabilitation?
- Anything else you would like to say about managing patients with long-COVID that might be of use to this study?

RGU ABERDEEN nmahp-ru Nursing, Midwifery and Allied Health Professions Research Unit University of Dundee

For peer review only - http://bmjopen.bmj.com/site/about/guidelines.xhtml
